# SCD‐*plus* features and AD biomarkers in cognitively unimpaired samples: A meta‐analytic approach for nine cohort studies

**DOI:** 10.1002/alz.14307

**Published:** 2025-02-22

**Authors:** Elizabeth Kuhn, Hannah M. Klinger, Rebecca E. Amariglio, Michael Wagner, Frank Jessen, Emrah Düzel, Michael T. Heneka, Gael Chételat, Dorene M. Rentz, Reisa A. Sperling, Jarith L. Ebenau, Elke Butterbrod, Wiesje M. Van Der Flier, Sietske A. M. Sikkes, Charlotte E. Teunnissen, Argonde C. Van Harten, Elsmarieke M. Van De Giessen, Lorena Rami, Adria Tort, Gonzalo Sánchez Benavides, Katherine A. Gifford, Carol Van Hulle, Rachel F. Buckley, Alzheimer's Disease Neuroimaging Initiative, Australian Imaging Biomarkers and Lifestyle flagship study of ageing, A4 Study Team, DELCODE Study, Frederic Brosseron, Katharina Buerger, Christoph Laske, Robert Perneczky, Oliver Peters, Joseph Priller, Alfredo Ramirez, Anja Schneider, Annika Spottke, Stefan Teipel, Jens Wiltfang, Harvard Aging Brain Study

**Affiliations:** ^1^ German Center for Neurodegenerative Diseases (DZNE) Bonn Bonn Germany; ^2^ Department of Cognitive Disorders and Old Age Psychiatry University Hospital Bonn Bonn Germany; ^3^ Department of Neurology Massachusetts General Hospital Boston Massachusetts USA; ^4^ Center for Alzheimer Research and Treatment (CART) Brigham & Women's Hospital Boston Massachusetts USA; ^5^ Department of Psychiatry University of Cologne Medical Faculty Cologne Germany; ^6^ Excellence Cluster on Cellular Stress Responses in Aging‐Associated Diseases (CECAD) University of Cologne Cologne Germany; ^7^ German Center for Neurodegenerative Diseases (DZNE) Magdeburg Germany; ^8^ Institute of Cognitive Neurology and Dementia Research (IKND) Otto‐von‐Guericke University Universitätsplatz 2 Magdeburg Germany; ^9^ Luxembourg Centre for Systems Biomedicine (LCSB) University of Luxembourg Belvaux Esch‐sur‐Alzette Luxembourg; ^10^ Normandie Univ, UNICAEN, INSERM, U1237 Physiopathology and Imaging of Neurological Disorders (PhIND) Neuropresage Team, Cyceron Caen cedex France; ^11^ Harvard Medical School Brigham and Women's Hospital Boston Massachusetts USA; ^12^ Alzheimer Center Amsterdam, Neurology Vrije Universiteit Amsterdam, Amsterdam UMC location VUmc Amsterdam The Netherlands; ^13^ Amsterdam Neuroscience, Neurodegeneration Amsterdam The Netherlands; ^14^ Department of Clinical, Neuro and Developmental Psychology Vrije Universiteit Amsterdam Amsterdam The Netherlands; ^15^ Department of Neurosurgery Elisabeth‐Tweesteden Hospital Tilburg The Netherlands; ^16^ Epidemiology and Data Science Vrije Universiteit Amsterdam, Amsterdam UMC location VUmc Amsterdam The Netherlands; ^17^ Neurochemistry Laboratory Department of Laboratory Medicine Amsterdam Neuroscience, Amsterdam University Medical Center Vrije Universiteit Amsterdam The Netherlands; ^18^ Department of Radiology and Nuclear Medicine Amsterdam University Medical Center Vrije Universiteit Amsterdam The Netherlands; ^19^ Hospital Clinic. Fundació Clinic August Pi i Sunyer Biomedical Research Institute (IDIBAPS) Barcelona Spain; ^20^ Barcelonaβeta Brain Research Center (BBRC) Pasqual Maragall Foundation Barcelona Spain; ^21^ Vanderbilt Memory and Alzheimer's Center Department of Neurology Vanderbilt University Medical Center Nashville Tennessee USA; ^22^ Department of Medicine School of Medicine and Public Health University of Wisconsin‐Madison Madison Wisconsin USA; ^23^ Wisconsin Alzheimer's Disease Research Center School of Medicine and Public Health University of Wisconsin‐Madison Madison Wisconsin USA; ^24^ Melbourne School of Psychological Sciences University of Melbourne Melbourne Australia

**Keywords:** Alzheimer's disease, amyloid pathology, cerebrospinal fluid, meta‐analysis, positron emission tomography, SCD‐Initiative, subjective cognitive decline, tau burden

## Abstract

**INTRODUCTION:**

Specific features of subjective cognitive decline (SCD‐*plus*) have been proposed to indicate an increased risk of preclinical Alzheimer's disease (AD). However, few studies have examined how these features relate to AD biomarkers in cognitively unimpaired (CU) older adults.

**METHODS:**

Meta‐analyses were performed using cross‐sectional data from nine cohorts (*n* = 7219, mean age (SD): 71.17 (5.9), 56.5% female) to determine associations of SCD‐*plus* features with positron emission tomography (PET)– or cerebrospinal fluid (CSF)–derived amyloid beta (Aβ) and tau biomarkers.

**RESULTS:**

Participants with preclinical AD (community‐based only) were more likely to fulfill SCD‐*plus* features. The presence of self‐reported memory decline, associated concern/worry, and a higher number of fulfilled features were all associated with high Aβ levels. Only the latter was associated with abnormal tau.

**DISCUSSION:**

Simultaneous endorsement of multiple SCD‐*plus* features is a robust indicator of abnormal AD biomarkers in CU older adults, whereas isolated SCD features seem only sensitive to elevated Aβ, supporting their value as early behavioral markers of preclinical AD.

**Highlights:**

- About two‐tenths of our sample had abnormal amyloid beta (Aβ) levels with evidence of subjective cognitive decline (SCD).
- Preclinical AD subsamples (community‐based) had a higher percentage of participants meeting SCD‐*plus* features. Self‐reported memory decline and concern/worry were the sole features associated with high Aβ, but not tau, burden.
- A higher number of fulfilled SCD‐*plus* features are linked to high Aβ and tau burden.
- Use of multiple SCD‐*plus* features may help identify early stages of biological AD.

## 1 BACKGROUND

Subjective cognitive decline (SCD) refers to the perception of a deterioration in cognitive abilities compared to a previous level of performance.^1^ Evidence suggests that approximately 33% of adults age 65 or older experience SCD without any objective deficits as measured by neuropsychological tests.^1^, ^2^ These individuals are at higher risk for future cognitive decline^3^, ^4^, ^5^ and Alzheimer's disease (AD) dementia,^6^, ^7^ and appear to have greater neurodegeneration in AD‐vulnerable regions,^8^, ^9^, ^10^ although not all will develop subsequent objective cognitive decline.

In this context, the SCD‐Initiative has proposed specific features of SCD (SCD‐*plus*) that increase the likelihood of being in a preclinical stage of AD in cognitively unimpaired (CU) older adults with SCD. These are the presence of a self‐reported memory decline (SMD) rather than decline in other cognitive domains, onset of the decline within the last 5 years and after age 60, presence of an associated concern/worry, feeling of worse performance than peers of the same age, confirmation of the decline by a study partner when possible, and more recently proposed: seeking medical help and persistence of the decline over time (listed in Table 1).^1^, ^11^ The presence of each of these features increases the risk of future cognitive decline and/or clinical progression to mild cognitive impairment (MCI) and dementia, and their co‐occurrence further increases these risks.^12^, ^13^, ^14^, ^15^ However, the specific association of these SCD‐*plus* features with AD biomarkers is not yet fully understood, as most previous studies have been limited to one feature^16^, ^17^, ^18^ and/or one biomarker.^19^, ^20^, ^21^, ^22^ Overall, some studies have suggested that self‐reported SMD is associated with lower amyloid beta (Aβ) cerebrospinal fluid (CSF) levels^20^, ^23^ or higher positron emission tomography (PET) Aβ cortical load^24^, ^25^ and possibly with an abnormal tau PET in the entorhinal cortex,^17^ although this was not found in all studies.^8^, ^16^, ^26^ For the other SCD‐*plus* features, associations with AD biomarkers were also inconsistent across studies^16^, ^20^, ^21^, ^27^ and require further validation in a larger number of participants across cohorts from different settings.

**TABLE 1:** Specific features of subjective cognitive decline (SCD‐*plus*) proposed by the SCD‐Initiative to be associated with an increased likelihood of preclinical Alzheimer's Disease in individuals cognitively unimpaired.

**SCD‐*plus* features proposed by the SCD‐Initiative** ^1^, ^11^	
Subjective decline in memory, rather than other domains of cognition	
Onset of SCD within the last 5 years	
Age at onset of SCD ≥60 years old	
Concerns (worries) associated with SCD	
Feeling of worse performance than others of the same age group	
If available: Confirmation of cognitive decline by a study partner	
**More recently proposed**:	
Persistence of the SCD over time	
Medical help seekers	

This is particularly important because the National Institute on Aging–Alzheimer's Association (NIA‐AA) research framework suggests that the presence of SCD, or subtle objective cognitive decline, combined with abnormal Aβ may correspond to clinical stage 2 of the biological AD continuum, whereas abnormal Aβ alone (without SCD) may correspond to clinical stage 1.^28^ Indeed, previous studies have shown that isolated abnormal Aβ levels in CU older adults are less predictive of future cognitive decline than when combined with abnormal tau levels (i.e., preclinical AD^29^, ^30^) or with the presence of SCD.^31^, ^32^, ^33^, ^34^, ^35^ This highlights the importance of (1) better understanding the frequency of endorsement of SCD features in individuals with preclinical AD pathological changes, and also of (2) identifying the features of SCD that are most sensitive to either Aβ and tau levels when the biomarker status is unknown, in order to improve clinical trial inclusion criteria. These are the two main aims of the present study, which uses participant‐level data from nine cohorts included in the *SCD‐plus Amyloid & Tau* working group within the SCD Professional Interest Area (Alzheimer's Association International Society to Advance Alzheimer's Research and Treatment ‐ ISTAART). We also examined whether the incremental endorsement of multiple SCD‐*plus* features would show associations similar to those found using a single feature.

## 2 METHODS

### 2.1 Study populations

We included participants from nine cohorts: Anti‐Amyloid Treatment in Asymptomatic Alzheimer Disease (A4^36^) Study, Alzheimer's Disease Neuroimaging Initiative (ADNI^37^, ^38^), Harvard Aging Brain Study (HABS^39^), Vanderbilt Memory and Aging Project (VMAP^40^), and Wisconsin Registry for Alzheimer's Prevention (WRAP^41^) in the United States; Australian Imaging, Biomarker & Lifestyle flagship study of ageing (AIBL^42^) in Australia, DZNE Longitudinal Cognitive Impairment and Dementia Study (DELCODE^43^) in Germany, Imagerie Multimodale de la maladie d'Alzheimer à un stade Précoce (IMAP+^8^, ^44^) in France, and Subjective Cognitive Impairment Cohort (SCIENCe^21^) in The Netherlands. Cohorts were included based on the following eligibility criteria: (1) participants were CU older adults (≥50 years) who did not meet criteria for MCI or dementia, (2) recruited from memory clinics or through public advertising in the community (SCD and CU subgroups, respectively), (3) assessed for at least two SCD‐*plus* features^1^, ^11^ (see SCD‐*plus* features section below), (4) had data available for at least Aβ PET or CSF and, when possible, also for tau PET or CSF. The profiles of the included cohorts are shown in Table 2. For the current analyses, a total of 7219 participants (*N* = 7058 with Aβ data, *N* = 2232 with tau data, and *N* = 2071 with both; Table S1) were included. For specific analyses (see Statistical analyses section below) we then divided the cohorts into 13 subgroups based on the biomarker modality used (PET or CSF) and the recruitment setting (community‐based CU older adults or SCD patients recruited from memory clinics).

**RESEARCH IN CONTEXT**

1. **Systematic review**: Associations between subjective cognitive decline (SCD) and amyloid beta (Aβ) or tau pathology burden are inconsistent across studies, highlighting the importance of better understanding of which specific features of SCD (SCD‐*plus*) are associated with Alzheimer's disease (AD) biomarkers.
2. **Interpretation**: Individuals with preclinical AD were more likely to fulfill at least one of the examined SCD‐*plus* features (specific to community‐based, non‐SCD‐*plus* enriched cohorts). Using participant‐level data, we observed significant associations between high Aβ (status and level) and self‐reported memory decline and associated concern/worry. Simultaneous fulfillment of multiple SCD‐*plus* features was a robust indicator of abnormal AD biomarker levels of both Aβ and tau, highlighting the relevance of increasing SCD‐*plus* severity in identifying individuals at risk for biological AD.
3. **Future directions**: Future studies are needed to identify additional SCD features (e.g., study partner–reported) that should also be considered, and to extend this research to markers of clinical progression.

**TABLE 2:** Profile of the cohort studies.

Cohort	A4	ADNI	AIBL	DELCODE	HABS	IMAP+	SCIENCe	VMAP	WRAP
**Design**	Multicentric (67 sites)	Multicentric (63 sites)	Multicentric (2 sites)	Multicentric (10 sites)	Monocentric	Monocentric	Monocentric	Monocentric	Monocentric
**Location**	USA, Canada, Australia, Japan	USA, Canada	Australia	Germany	USA	France	The Netherlands	USA	USA
**Starting year**	2014	2004	2009	2014	2014	2008	2014	2012	2001
**Registration**	NCT02008357	NCT00106899	NA	DRKS00007966	NIH‐P01AG036694	NCT01638949	NA	NCT05372159	NA
**Recruitment setting** ^a^	Public advertising	Public advertising	Public advertising	Public advertising and memory clinic consultation	Public advertising	Public advertising and memory clinic consultation	Memory clinic consultation	Public advertising	Public advertising
**Age**	65–85 years	≥55 years	≥60 years	≥60 years	≥65 years	≥50 years	≥45 years	≥50 years	≥40 years
**SMD**	X	X	X	X	X	X	X	X	X
**Worry**	X	X		X	X			X	X
**Peer**		X	X	X	X	X	X	X	
**Onset**				X		X		X	
**SCD‐Severity (range)**	0–2	0–3	0–2	0–4	0–3	0‐3	0—2	0‐4	0‐2
**Amyloid‐positivity**	FBP‐PET [SUVr ≥1.10, confirmed by visual read when <1.15]	FBP‐PET [SUVr ≥1.11] CSF Aβ_42_ [<977 pg/mL]	PiB‐PET [SUVr ≥1.4]	CSF ratio Aβ_42/40_ [<0.0806]	PiB‐PET [DVR ≥1.186]	FBP‐PET [SUVr≥1.24]	Amyloid‐PET^b^ [visual read] CSF Aβ_42_ [<813 pg/mL]	CSF Aβ_42_ [≤526 ng/L]	CSF ratio Aβ_42/40_ [<0.0046]
**Tau‐positivity**	FTP‐PET [SUVr>1.30]	FTP‐PET [SUVr >1.30] CSF ptau_181_ [>27 pg/mL]	NA	CSF ptau_181_ [>73.6596 pg/mL]	FTP‐PET [SUVr>1.26]	NA	CSF ptau_181_ [>52pg/mL]	CSF ptau_181_ [≥ 50.2ng/L]	CSF ptau_181_ [≥24.8pg/ml]

Abbreviations: A4, Anti‐Amyloid Treatment in Asymptomatic Alzheimer Disease Study^36^; ADNI, Alzheimer's Disease Neuroimaging Initiative^37^, ^38^; AIBL, Australian Imaging, Biomarker & Lifestyle flagship study of ageing^42^; CSF, cerebrospinal fluid; CU, cognitively unimpaired; DELCODE, DZNE Longitudinal Cognitive Impairment and Dementia Study^43^; DVR, distribution volume ratio; FBP, 18‐F‐Florbetapir; FTP, 18‐F‐Flortaucipir; HABS, Harvard Aging Brain Study^39^; IMAP+, Imagerie Multimodale de la maladie d'Alzheimer à un stade Précoce^8^, ^44^; PET, positron emission tomography; PiB, 11‐C‐Pittsburgh compound B; SCD, patients with subjective cognitive decline recruited from memory clinic; SCIENCe, Subjective Cognitive Impairment Cohort^21^; SMD, Self‐reported Memory Decline; SUVr, global standardized uptake value ratio; VMAP, Vanderbilt Memory and Aging Project^40^; WRAP, Wisconsin Registry for Alzheimer's Prevention.^41^

^a^ Within each cohort, participants were excluded when unstable or exclusionary medical or psychiatric problems.

^b^ Florbetapir‐, Flutemetamol‐, and Florbetaben‐PET.

### 2.2 SCD‐*plus* features

In each cohort, SCD‐*plus* features were assessed using either self‐report questionnaires (yes/no or Likert scale response) and/or with structured interviews.^3^, ^20^, ^21^, ^40^, ^41^, ^42^, ^45^, ^46^, ^47^, ^48^, ^49^, ^50^ Self‐reported SCD‐*plus* features included self‐reported SMD, associated concerns/worries, feeling worse performance than peers of the same age, and SCD onset in the last 5 years.^1^, ^11^ It should be noted that among the list of currently known SCD‐*plus* features (Table 1), medical help‐seeking could not be assessed directly due to a lack of relevant items, but was examined via the recruitment setting (community‐based vs memory clinic‐recruited). Persistence of SCD over time could not be assessed due to our cross‐sectional design, and study partner confirmation was not analyzed here because we focused only on self‐reports in the current meta‐analysis.

Table 2 provides information on the SCD‐*plus* features available within each cohort. All SCD‐*plus* features were dichotomized as present or absent according to the methods and assessments detailed in Tables S2–S3. In the main analyses, a gold‐standard approach is used, meaning that investigators from each cohort select the item that best reflects each SCD‐*plus* feature when multiple items are available (except for the VMAP and WRAP cohorts, where no standard exists and a multiple‐item approach was used).

The number of fulfilled SCD‐*plus* features was determined as the sum of all SCD‐*plus* features fulfilled, ranging from 0–2 to 0–4 across the cohorts. To facilitate comparability between cohorts, a mean score was computed, that is, the number of fulfilled features divided by the number of SCD‐*plus* features available in the cohort, hereafter called mean SCD‐severity score.

In additional analyses, a multiple‐item approach was used to test the consistency of results between different methodological approaches to determine SCD‐*plus* endorsement. In brief, 444 items were reviewed by six coders from the working group and classified according to whether or not they represented a specific SCD‐*plus* feature. The items selected by consensus correspond to those with 50% (3/6) coders’ agreement; that is, 220 self‐reported SMD items, 9 concern/worry items, 10 peer items, and 3 onset items. Unfortunately, some of these 242 selected items were part of questionnaires with a high proportion of missing data within cohorts. To increase the sample size in our analyses, four cohorts–ADNI, AIBL, HABS, and VMAP–finally exclude questionnaires/items with more than 10% missing data (see Table S2). Using this approach, participants had to meet more than 20% of the selected items to be considered as endorsing the SCD‐*plus* features (e.g., 1/5 to 4/19 items available).

### 2.3 Alzheimer's disease biomarkers

Aβ deposition was assessed by Aβ‐PET imaging in six cohorts (global standardized uptake value ratio [SUVr] or distribution volume ratio [DVR] extracted from 18‐F‐Florbetapir, 18‐F‐Florbetaben or 11‐C‐Pittsburgh Compound B [PiB]; *N* = 6026 participants) or by CSF levels in five cohorts (Aβ_42_ or Aβ_42/40_ ratio; *N* = 1032 participants). Tau pathology burden was assessed by 18‐F‐Flortaucipir‐PET in three cohorts (using mean SUVr in regions of interest within the temporal lobe, *N* = 1138 participants) and by CSF phosphorylated tau‐181 (p‐tau_181_) levels in five cohorts (*N* = 1094 participants). Two cohorts had both PET and CSF measures available (i.e., ADNI and SCIENCe), and are therefore divided into distinct groups of interest, with only one measure selected for each participant to avoid overlap in the study analyses. Aβ and tau positivity are determined using established cohort‐specific thresholds or visual reading^8^, ^21^, ^36^, ^37^, ^38^, ^39^, ^40^, ^41^, ^42^, ^43^, ^44^, ^51^, ^52^, ^53^, ^54^ to classify participants as Aβ negative (Aβ‐) or Aβ positive (Aβ+), and as tau negative (T–) or tau positive (T+), respectively (Table 2).

### 2.4 Statistical analyses

All analyses were performed with R 4.2.2 (R Foundation; https://cran.r‐project.org/bin/windows/base/old/4.2.2), and adjusted for multiple comparisons with a Bonferroni correction (α < 0.05 / 5 scores examined = 0.01).

Means and standard deviations (SDs), or sample sizes with frequencies (%), were used to describe the demographic and cognitive characteristics of each cohort according to the biomarker modality used and recruitment setting.

A two‐stage approach was used to conduct meta‐analyses using individual participant data (IPD). In the first stage (at the cohort level), each representative working group member assessed the frequency of each SCD‐*plus* feature endorsement stratified by high versus low Aβ, and, when available, high versus low tau levels and their combination according to the approaches defined in Section 2.2 (i.e., GOLD Standard and Multiple items; see Tables S2–S3). General linear models were then conducted to assess the cross‐sectional association between each SCD‐*plus* feature and Aβ and, when possible, tau in separate analyses. The same models were used to determine the association with the mean number of SCD‐*plus* features fulfilled (i.e., mean SCD‐severity score). The outcome of interest was primarily dichotomous high/low biomarker levels using logistic regression, and was repeated with continuous data using linear regression for validation purposes (i.e., to facilitate comparability between cohorts, z‐scores of PET‐SUVr, PET‐DVR, and CSF levels were used). All models were adjusted for age and sex. Finally, to determine whether the association with Aβ was independent of tau, and vice versa, we included each as an additional covariate in complementary sub‐analyses.

In a second stage, the summary statistics from these models were used to perform a two‐step meta‐analysis. First, meta‐proportions were calculated, using the *metafor* package via the *rma* function, to determine the proportion of participants with high Aβ and/or high tau levels (dichotomous), combined or not with the endorsement of at least one of the SCD‐*plus* feature examined (i.e., mean SCD‐severity score > 0). This allowed us to estimate the proportion of participants who might correspond to clinical stage 2 of the biological AD continuum^28^ in each cohort and across the entire sample, and also to determine whether the proportion of participants fulfilling at least one of the SCD‐*plus* features examined differed according to the presence or absence of preclinical AD (pathological changes). Second, the association of each SCD‐*plus* feature and mean SCD‐severity score with each outcome (Aβ and tau status or levels) was examined by extracting model estimates and standard errors from all cohorts to obtain pooled estimates. Associations were considered significant at the Bonferroni‐corrected threshold.^55^ Note that the estimates extracted from cohorts using Aβ‐CSF levels were reversed to match those from Aβ‐PET. The *I2* statistics were used to evaluate the degree of true heterogeneity between samples, with values of 25%, 50%, and 75% quantified as low, moderate, and high heterogeneity, respectively.^56^

In sensitivity analyses, the analyses were first performed adjusting for either Aβ or tau status. Second, stratified analyses were performed based on (1) recruitment setting (community‐based vs memory clinic‐recruited) and (2) biomarker modality used (PET vs CSF) to determine whether they were found in each subgroup. In addition, meta‐regressions were performed when subgroups included at least three cohorts to determine whether the strength of these associations was statistically different between these subgroups. Finally, analyses were replicated using the multiple‐item approach to test whether associations were found regardless of the method used to determine SCD‐*plus* feature endorsement.

## 3 RESULTS

Table 3 shows the baseline characteristics of participants in each cohort according to the modality used for Aβ and tau biomarkers and recruitment setting. Across nine cohorts, we included 7219 participants, mean age (SD) 71.17 (5.9) years, with a mean education level of 16.12 (2.99) years, of whom 4075 (56.5%) are women, 97.8% have available amyloid data including 1994 Aβ+ (28.3%), 30.9% have available tau data including 426 T+ (19.1%), and 28.7% have both available data including 255 Aβ+T+ (12.3%).

**TABLE 3:** Baseline characteristics of participants in the cohort studies

Cohort	Setting [Modality Aβ/T]	Sample size	Age in years, mean (sd)	Female, *N* (%)	Education in years, mean (sd)	Aβ+, *N* (%)	T+, *N* (%)	Aβ+T+, N(%)	*APOE* ε4 carriers, *N* (%)
**A4**	CU [PET/PET]	4493	71.29 (4.67)	2669 (59%)	16.58 (2.83)	1182/4492 (26.3%)	92/447 (20.6%)	82/446 (18.4%)	1554 (35%)
**ADNI**	CU [PET/PET]	431	73.04 (7.21)	174 (40%)	16.79 (2.34)	98/275 (35.6%)	53/431 (12.3%)	23/275 (8.4%)	142 (34%)
**ADNI**	CU [CSF/CSF]	268	74.71 (6.05)	126 (47%)	16.27 (2.67)	101/268 (37.7%)	69/268 (25.7%)	36/268 (13.4%)	67 (25%)
**AIBL**	CU [PET/NA]	768	72.26 (6.45)	436 (57%)	14.68 (3.10)	276/768 (35.9%)			172 (29%)
**DELCODE**	CU [CSF/ CSF]	138	67.64 (4.96)	74 (54%)	14.43 (2.67)	35/138 (25.4%)	10/138 (7.2%)	6/138 (4.3%)	37 (27%)
**DELCODE**	SCD [CSF/ CSF]	211	70.90 (5.84)	91 (43%)	14.88 (2.97)	83/211 (39.3%)	33/211 (15.6%)	26/211 (12.3%)	71 (34%)
**HABS**	CU [PET/PET]	352	72.07 (7.99)	210 (60%)	15.86 (2.96)	82/352 (23.3%)	48/260 (18.5%)	25/260 (9.6%)	96 (28%)
**IMAP+**	CU [PET/NA]	56	70.88 (5.98)	32 (57%)	12.14 (3.85)	13/56 (23.2%)			12 (21%)
**IMAP+**	SCD [PET/NA]	24	66.96 (7.90)	10 (42%)	12.88 (3.42)	7/24 (29.2%)			4 (17%)
**SCIENCe**	SCD [PET/CSF]	59	61.75 (7.72)	28 (48%)	13.10 (3.22)	12/59 (20.3%)	20/59 (33.9%)	9/59 (15.22%)	25/58 (42%)
**SCIENCe**	SCD [CSF/CSF]	98	61.18 (7.03)	42 (44%)	12.76 (3.00)	33/95 (34.7%)	36/95 (37.9%)	14/96 (14.6%)	39/93 (41%)
**VMAP**	CU [CSF/CSF]	82	72.01 (6.53)	25 (30%)	16.76 (2.43)	13/82 (15.9%)	24/82 (29.3%)	5/82 (6.1%)	24 (29.3%)
**WRAP**	CU [CSF/CSF]	239	65.57 (6.49)	158 (66%)	16.22 (2.46)	59/238 (24.8%)	41/239 (17.1%)	29/238 (12.2%)	86 (36%)
**Total**	**All**	**7219**	**71.17 (5.9)**	**4075/7219 (56.5%)**	**16.12 (2.99)**	**1994/7058 (28.3%)**	**426/2232 (19.1%)**	**255/2071 (12.3%)**	**2329/7213 (32.3%)**

Abbreviations: Aβ, amyloid; Aβ+, amyloid‐positivity; A4, Anti‐Amyloid Treatment in Asymptomatic Alzheimer Disease Study^36^; ADNI, Alzheimer's Disease Neuroimaging Initiative^37^, ^38^; AIBL, Australian Imaging, Biomarker & Lifestyle flagship study of ageing^42^; *APOE*, apolipoprotein E; CSF, cerebrospinal fluid; CU, cognitively unimpaired; DELCODE, DZNE Longitudinal Cognitive Impairment and Dementia Study^43^; HABS, Harvard Aging Brain Study^39^; IMAP+, Imagerie Multimodale de la maladie d'Alzheimer à un stade Précoce^8^, ^44^; *N*, sample size; PET, positron emission tomography; SCD, patients with subjective cognitive decline recruited from memory clinic; SCIENCe, Subjective Cognitive Impairment Cohort^21^; T, tau; T+, tau‐positivity; VMAP, Vanderbilt Memory and Aging Project^40^; WRAP, Wisconsin Registry for Alzheimer's Prevention.^41^

### 3.1 Frequency of SCD‐*plus* features endorsement by Aβ and tau status

**FIGURE 1 alz14307-fig-0001:**
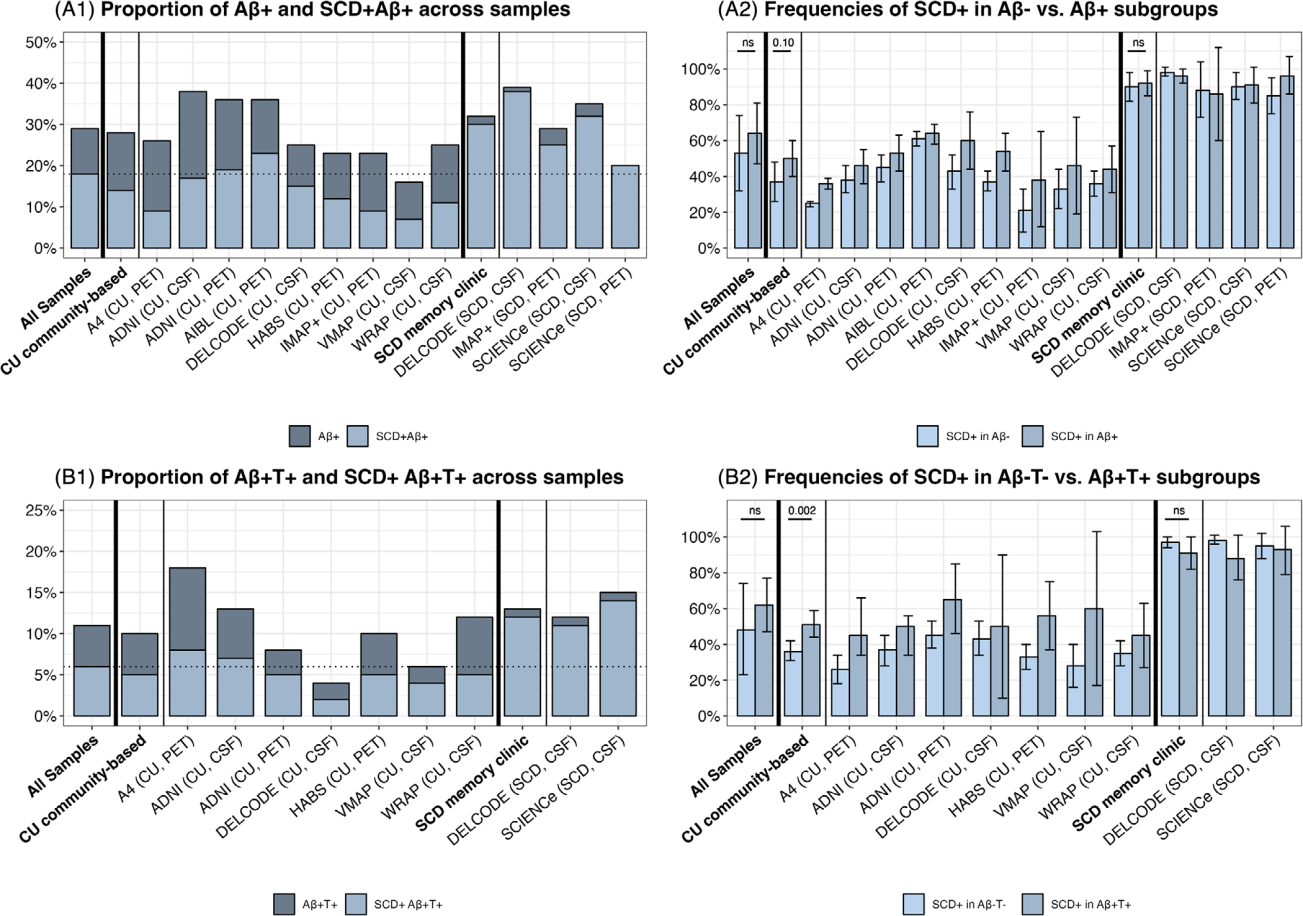
Proportion of participants with preclinical AD pathological changes who also endorsed at least one of the four SCD‐*plus* features examined (A1–B1, SCD+Aβ+[T+]), and the corresponding frequency of SCD endorsement among participants with/without evidence of AD pathology (A2–B2, SCD+ in Aβ+[T+] vs Aβ–[T–]). CSF, cerebrospinal fluid; Concern, Associated concern/worry about SCD; CU, cognitively unimpaired older adults recruited from the community; Onset, onset of the subjective cognitive decline within the past 5 years; Peer, feeling of worse performance than peers of the same age; PET, positron emission tomography; SCD, patients with subjective cognitive decline recruited from memory clinics; SMD, self‐reported subjective memory decline.

The frequencies of endorsement of at least one SCD‐*plus* feature combined with Aβ and/or tau status are shown in Figure 1, and detailed cohort information is provided in Table S4. In brief, 18% [13%–22%] of all participants have abnormal Aβ levels and a mean SCD‐severity score greater than 0 (Figure 1A1; SCD+Aβ+), and 6% [4%–8%] of participants across all samples also have abnormal tau levels (Figure 1B1; SCD+Aβ+T+), compared to 37% [29%–45%] and 27% [17%–37%] with mean SCD‐severity scores greater than 0 but normal Aβ levels (SCD+Aβ–) and tau levels (SCD+Aβ–T–), respectively. This results in a mean SCD severity score greater than 0 for 64% [47%–81%] and 62% [47%–77%] of Aβ+ and Aβ+T+ participants, compared to 53% [32%–74%] and 48% [23%–74%] of Aβ– and Aβ–T– participants, respectively. When compared to the frequency of SCD endorsement within Aβ– and Aβ–T– participants, these frequencies were significantly higher only in the CU community‐based subgroup for Aβ+T+ versus Aβ–T– participants (51% [44%–59%] vs 36% [31%–42%], Q_M _= 10.07, *p *= 0.002; Figure 1A2), but not for Aβ+ versus Aβ– participants (50% [40%–60%] vs 37% [26%–48%], Q_M _= 2.77, *p *= 0.10; Figure 1B2). There were no differences in the overall sample (all *p*’s > 0.36) in the SCD subgroup recruited from memory clinics (all *p*’s > 0.22, Table S4), or for individual SCD‐*plus* feature (all *p’*s > 0.18, detailed information shown in Figure S1 and S2).

All these analyses showed a high heterogeneity between samples, with frequencies of SCD+Aβ+(T+) or SCD endorsement within Aβ+(T+) always significantly higher in SCD recruited from memory clinics compared to CU community‐based subgroups (all *p’*s < 0.01; 9.92 ≤ Q_M_ ≤ 11.00 and 42.41 ≤ Q_M_ ≤ 45.65, respectively), and no differences between CSF and PET subgroups (all *p’*s > 0.35; 0.00 ≤ Q_M_ ≤ 0.49 and 0.22 ≤ Q_M_ ≤ 0.89, respectively; Table S4).

### 3.2 SCD‐*plus* features related to Alzheimer's disease biomarkers

**FIGURE 2:**
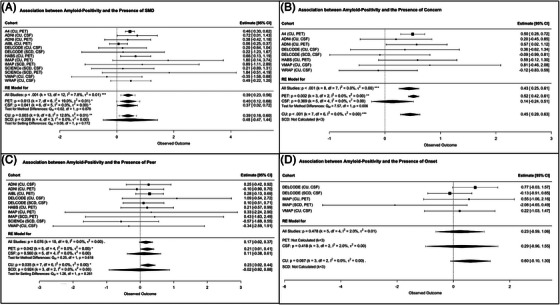
Association between amyloid‐positivity and self‐reported SCD‐*plus* features. (A) Self‐reported memory decline (SMD). (B) Associated concern/worry. (C) Feeling of worse performance than peers of the same age. (D) Onset of the subjective cognitive decline within the last 5 years. A4, Anti‐Amyloid Treatment in Asymptomatic Alzheimer Disease Study^36^; ADNI, Alzheimer's Disease Neuroimaging Initiative ^37^, ^38^; AIBL, Australian Imaging, Biomarker & Lifestyle Flagship Study of Ageing^42^; CI, confidence interval; CSF, cerebrospinal fluid; CU, cognitively unimpaired older adults recruited from the community; DELCODE, DZNE Longitudinal Cognitive Impairment and Dementia Study ^43^; HABS, Harvard Aging Brain Study ^39^; IMAP+, Imagerie Multimodale de la maladie d'Alzheimer à un stade Précoce ^8^, ^44^; PET, positron emission tomography; SCD, patients with subjective cognitive decline recruited from memory clinic; SCIENCe, Subjective Cognitive Impairment Cohort ^21^; VMAP, Vanderbilt Memory and Aging Project ^40^; WRAP, Wisconsin Registry for Alzheimer's Prevention.^41^

Regarding Aβ, the presence of SMD and associated concern/worry were both significantly associated with Aβ status (Figure 2). No significant associations were found for the feeling of worse performance than peers of the same age and the SCD onset within the last 5 years. A similar pattern of findings existed for continuous Aβ levels (SMD: k = 12, 0.22 [0.17–0.26], *p *< 0.001, I^2 ^= 0.0%; associated concern/worry: k = 8, 0.20 [0.05–0.34], *p *= 0.015, I^2 ^= 33.4%; Figure S3). Overall, analyses showed a low heterogeneity between samples.

**FIGURE 3:**
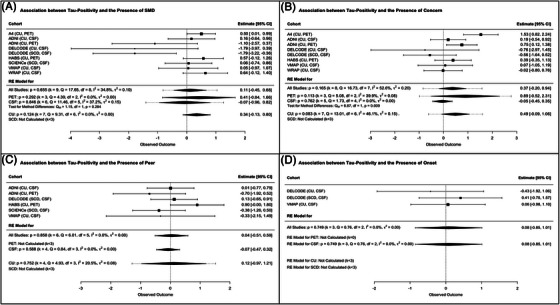
Association between tau‐positivity and self‐reported SCD‐*plus* features. (A) Self‐reported memory decline (SMD). (B) Associated concern/worry. (C) Feeling of worse performance than peers of the same age. (D) Onset of the subjective cognitive decline within the last 5 years. A4, Anti‐Amyloid Treatment in Asymptomatic Alzheimer Disease Study ^36^; ADNI, Alzheimer's Disease Neuroimaging Initiative ^37^, ^38^; AIBL, Australian Imaging, Biomarker & Lifestyle Flagship Study of Ageing ^42^; CI, confidence interval; CSF, cerebrospinal Fluid; CU, cognitively unimpaired older adults recruited from the community; DELCODE, DZNE Longitudinal Cognitive Impairment and Dementia Study^43^; HABS, Harvard Aging Brain Study^39^; IMAP+, Imagerie Multimodale de la maladie d'Alzheimer à un stade Précoce^8^, ^44^; PET, positron emission tomography; SCD, patients with subjective cognitive decline recruited from memory clinic; SCIENCe, Subjective Cognitive Impairment Cohort^21^; VMAP, Vanderbilt Memory and Aging Project^40^; WRAP, Wisconsin Registry for Alzheimer's Prevention.^41^

By contrast, the four SCD‐*plus* features were not associated with tau status (Figure 3) or with continuous tau levels (Figure S4). Overall, analyses showed low to moderate heterogeneity between samples.

### 3.3 Mean SCD‐severity score in relation to Alzheimer's disease biomarkers

**FIGURE 4:**
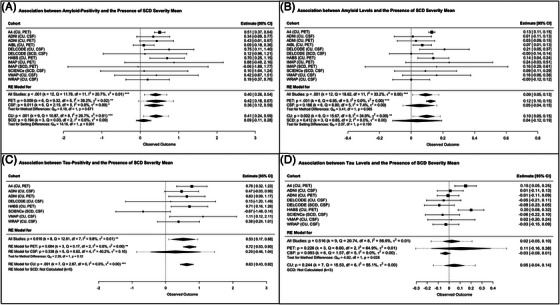
Association between the number of fulfilled SCD‐*plus* features (i.e., mean SCD‐severity score) and both amyloid‐ and tau‐pathology. (A) Association with amyloid‐positivity. (B) Association with amyloid‐levels. (C) Association with tau‐positivity. (D) Association with tau‐levels. A4, Anti‐Amyloid Treatment in Asymptomatic Alzheimer Disease Study ^36^; ADNI, Alzheimer's Disease Neuroimaging Initiative ^37^, ^38^; AIBL, Australian Imaging, Biomarker & Lifestyle Flagship Study of Ageing ^42^; CI, confidence interval; CSF, cerebrospinal Fluid; CU, cognitively unimpaired older adults recruited from the community; DELCODE, DZNE Longitudinal Cognitive Impairment and Dementia Study ^43^; HABS, Harvard Aging Brain Study ^39^; IMAP+, Imagerie Multimodale de la maladie d'Alzheimer à un stade Précoce ^8^, ^44^; PET, positron emission tomography; SCD, patients with subjective cognitive decline recruited from memory clinic; SCIENCe, Subjective Cognitive Impairment Cohort ^21^; VMAP, Vanderbilt Memory and Aging Project ^40^; WRAP, Wisconsin Registry for Alzheimer's Prevention.^41^ ^.^

Higher mean SCD‐severity score was significantly associated with both Aβ and tau status in separate models (Figure 4 A,C). For the tau model, the DELCODE SCD sample was removed due to extreme estimates more than 3 SD from the group effect. Similar associations were found with continuous Aβ levels, but not for continuous tau levels (Figure 4B,D). Overall, analyses suggested a low to moderate heterogeneity between samples.

### 3.4 Sensitivity analyses

#### 3.4.1 Covarying for biomarkers status

In Table S5, we report that adjustment for tau status in the Aβ models, or for Aβ status in the tau models, did not affect our results, although not all previous associations held after Bonferroni correction (e.g., associations with Aβ levels, 0.05 > *p* > 0.01). No new associations were found. These analyses showed a low to moderate between‐sample heterogeneity.

#### 3.4.2 Influence of recruitment setting

Most associations were replicated in the CU community‐based subgroup and survived Bonferroni correction (Figures 2 and 4, and Figures S3 and S4). We found one new association between higher continuous Aβ levels and the SCD onset within the last 5 years (Figure S3). Regarding the SCD memory clinic‐recruited subgroup, data were only available in more than two cohorts for the presence of a SMD, the feeling of worse performance than peers of the same age, and the mean SCD‐severity. No significant associations were found in this subgroup. The only difference in the strength of the associations between the CU and SCD subgroups was for the association between Aβ status and a higher mean SCD‐severity, which appeared to be specific to the CU subgroup here (*p *< 0.001, Figure 4). Overall, these analyses showed a low to moderate heterogeneity between samples.[Fig alz14307-fig-0001]

#### 3.4.3 Influence of biomarker modality

Our findings were largely replicated in both the PET and CSF subgroups, although most of the results in the CSF subgroup did not survive the Bonferroni correction (0.05 < *p *< 0.01, Figures 2, 3, 4, and Figures S3 and S4). There was only one difference in the strength of the associations between the PET and CSF subgroups, with the association between Aβ status and associated concern/worry specific to the PET subgroup. Overall, these analyses showed a low to high heterogeneity between samples.

#### 3.4.4 Influence of approaches used to determine SCD‐*plus* endorsement

The gold‐standard and the multiple‐item approaches showed the same pattern of effect, with only minor differences; that is, the association between an associated concern/worry and Aβ status or levels did not withstand Bonferroni correction; nor did the association between mean SCD‐severity score and tau status (0.05 > *p *> 0.01; Table S6).

## 4 DISCUSSION

The main finding of our study is that endorsement of (multiple) SCD‐*plus* features appears to be a robust indicator of abnormally elevated AD biomarker levels in CU older adults, with more frequent endorsement in participants with preclinical AD (community‐based only) and significant associations with Aβ and/or tau pathology burden. These features may thus represent early behavioral markers of preclinical AD (particularly the presence of SMD and associated concern/worry), with simultaneous endorsement of multiple SCD‐*plus* features suggesting a more advanced biological stage.

In the current study, 27%–30% (17%–36%) of participants had preclinical AD pathological changes as defined by high Aβ burden on PET or CSF (Aβ+ in different subsamples). When tau information was added in a smaller sample of 2069 participants, we found that 6%–11% (1%–14%) had both abnormal Aβ and tau levels. These percentages are similar to those reported in previous studies.^57^, ^58^, ^59^ Of interest, our study provides information on the frequency of CU older adults who could qualify for the clinical stage 2 of the biological AD continuum in our samples, with 18% (13%–22%) of participants endorsing (multiple) SCD‐*plus* features and having abnormal Aβ levels (SCD+Aβ+), whereas only 6% (4%–8%) of participants also have abnormal tau levels (SCD+Aβ+T+). Although this represents a small proportion of our sample (approximately one‐ to two‐tenths), it is important to note that the endorsement of one or another SCD‐*plus* feature was actually quite common among participants with preclinical AD, affecting two‐thirds of these participants (half of the community‐based participants and the majority of SCD patients recruited from memory clinics). This represents approximately 10%–15% more than in participants without evidence of preclinical AD, meaning that they were more likely to perceive changes in their cognition, although frequencies differed significantly only in community‐based cohorts who were not already enriched for endorsement of SCD‐*plus* features (significant for Aβ+T+ vs Aβ–T–; at trend level for Aβ+ vs Aβ–).

In fact, there was high variability in all these frequencies within each cohort, which could be partly explained by the recruitment setting; that is, the frequencies of endorsement are, as expected, significantly higher in SCD patients recruited from memory clinics (an SCD‐*plus* enriched population). However, this does not explain all the heterogeneity, which remains quite high in stratified samples. Additional factors have been associated previously with abnormal Aβ levels in SCD patients and could influence these frequencies, such as older age and carrier status of the apolipoprotein E (*APOE*) ε4 allele.^19^, ^60^ Sociodemographic and cultural differences in the understanding and expression of SCD among individuals (e.g., racial, ethnic, and gender diversity),^61^ as well as differences in the surveys used and the phrasing of the questions, may also increase the heterogeneity of SCD. Previous work has highlighted the complexity and importance of harmonizing data across SCD surveys.^62^ Although our approach focused on a meta‐analysis, future studies should examine the same question using data harmonization with pooled cohort data.

However, in the second part of our analyses, most of the heterogeneity was resolved using individual‐level data and general linear models with covariates. We showed that two of the four self‐reported SCD‐*plus* features (i.e., SMD and associated concern/worry) were significantly associated with a greater likelihood of high Aβ burden (both status and continuous levels). None of the individual SCD‐*plus* features were associated with tau. We found that the associations with Aβ were particularly robust, surviving Bonferroni correction and adjustment for tau status. These findings partially confirm the results of a recent meta‐analysis (which had a smaller sample size, and was limited to participants/patients with defined SCD),^19^ and are consistent with some previous studies showing higher SCD levels in those with high Aβ burden when compared to those with low Aβ burden,^24^, ^25^, ^51^, ^63^ and significant correlations between higher SCD levels (mostly using SMD questionnaires) and higher Aβ burden in CU older adults or SCD patients.^17^, ^64^, ^65^ These associations were not always significant across studies,^8^, ^16^, ^26^, ^66^, ^67^ but, as in our study, appeared to vary depending on the SCD‐*plus* feature examined,^16^, ^20^ the questionnaire used,^16^, ^68^ or the cognitive domains assessed.^69^ Furthermore, the lack of association with tau burden seems to be in line with previous studies showing no significant association with CSF p‐tau_181_ levels^18^, ^20^, ^70^ or tau‐PET,^71^ although studies using PET reported more nuanced results. One study suggested an association with self‐reported SMD,^17^ and others have shown specific associations with subjective reports in different cognitive domains^69^ or in regions not used here to define the PET positivity threshold such as frontal regions.^72^ Our findings suggest that including the presence of an SMD and associated concern/worry may help to enrich interventional trials for individuals with preclinical AD pathological changes.

Of interest, the mean SCD‐severity score was associated with high Aβ burden (status and levels) and also with abnormally elevated tau levels in this study. As before, the associations found were robust and maintained in all sensitivity analyses. These findings were consistent regardless of the method used to measure the endorsement of SCD‐*plus* features, and only marginal differences were found when comparing the strength of associations by biomarker modality used or recruitment setting, although these were significant only in the subset of community‐based cohorts and the PET subgroup. This suggests that both Aβ and tau may independently contribute to the perception of a cognitive decline, and that concurrent endorsement of multiple SCD‐*plus* features was a robust indicator of abnormal AD biomarkers in CU older adults (both Aβ and tau), whereas isolated SCD‐*plus* features were most sensitive for Aβ biomarkers. This may highlight the temporal sequence of the AD pathological progression. Previous studies have shown that CU older adults with high levels of Aβ and tau,^29^, ^30^, ^73^ or with high levels of Aβ and SCD,^31^, ^32^, ^33^, ^34^, ^35^ have a higher risk of future cognitive decline and may be at a more advanced stage of the disease, compared to those with high Aβ alone who may never experience cognitive decline during their lifetime.^74^ A higher mean SCD‐severity would thus reflect these later stages and, in combination with abnormal AD biomarkers, represent a clinical stage 2b of biological AD (quite rare in our sample, about 6% [4%–8%] of participants are SCD+Aβ+T+). This may explain why the co‐occurrence of SCD‐*plus* features further increased the risk of clinical progression to MCI or dementia in previous studies.^12^, ^15^ We can, therefore, hypothesize that individuals on the biological AD continuum who are already experiencing cognitive difficulties (especially if they fulfill multiple SCD‐*plus* features) are those most likely to experience short‐term objective cognitive decline. Although this needs to be confirmed in future studies, we believe that they should be the primary target population for interventional trials, whereas greater caution should be exercised in completely asymptomatic individuals (Aβ+[T+] without SCD or subtle cognitive impairment). In addition, if the number of assessments must be limited, we recommend prioritizing information on SMD and associated concerns/worries, as these factors appear to be more sensitive in detecting AD pathology.

In the present study, we used data collected from a large number of cohorts with participants recruited in different settings and with different biomarker modalities. Pooling findings from similar analyses across cohorts allows for the identification of patterns that may not be apparent when individual studies are considered in isolation. A limitation of our study is that most cohorts did not include all of the SCD‐*plus* features evaluated here, leading to a relatively low statistical power for certain features, such as the SCD onset within the last 5 years. Only three cohorts included SCD patients recruited from memory clinics, making it impossible to compare findings according to the recruitment setting for some of the SCD‐*plus* features examined (e.g., associated concern/worry). Furthermore, this study focused on self‐reported SCD‐*plus* features and had a cross‐sectional design, and so it did not examine SCD reported by study partner, or the associations with markers of clinical progression (e.g., cognitive decline, AD biomarkers changes). Finally, all cohorts were highly educated, from high‐income countries, and largely of European ancestry. Replication of these analyses in populations from other regions of the world (e.g., Asia, South America, Africa) may, therefore, provide additional information on the applicability of these SCD‐*plus* features worldwide.

In conclusion, our results showed that CU older adults with preclinical AD (pathological changes) were more likely to fulfill SCD‐*plus* features than those without evidence of AD pathology, although the overall combination of SCD with abnormal AD biomarker levels remains quite rare in our sample. Our results also showed that two of the four SCD‐*plus* features (i.e., SMD and an associated concern/worry) are sensitive to abnormal Aβ levels, whereas the simultaneous endorsement of multiple SCD‐*plus* features is a robust indicator of preclinical AD (i.e., abnormally elevated Aβ and tau levels). Thus it may represent an early behavioral marker of preclinical AD and help to discriminate CU older adults with clinical stage 2 of the biological AD continuum, who should likely be targeted for interventional trials. Further research is needed to better understand the potential utility of study partner–reported measures, as well as the longitudinal relationship between these SCD features and markers of clinical progression, particularly in the context of biological AD, and their replicability in more diverse populations.

## CONFLICT OF INTEREST STATEMENT

Research programs of Wiesje van der Flier (WF) have been funded by ZonMW, NWO, EU‐JPND, EU‐IHI, Alzheimer Nederland, Hersenstichting CardioVascular Onderzoek Nederland, Health∼Holland, Topsector Life Sciences & Health, stichting Dioraphte, Gieskes‐Strijbis fonds, stichting Equilibrio, Edwin Bouw fonds, Pasman stichting, stichting Alzheimer & Neuropsychiatrie Foundation, Philips, Biogen MA Inc, Novartis‐NL, Life‐MI, AVID, Roche BV, Fujifilm, Eisai, and Combinostics. W.F. holds the Pasman chair. W.F. is recipient of ABOARD, which is a public–private partnership receiving funding from ZonMW (#73305095007) and Health∼Holland, Topsector Life Sciences & Health (PPP‐allowance; #LSHM20106). W.F. has been an invited speaker at Biogen MA Inc, Danone, Eisai, WebMD Neurology (Medscape), NovoNordisk, Springer Healthcare, and European Brain Council. W.F. is consultant to Oxford Health Policy Forum CIC, Roche, Biogen MA Inc, and Eisai. W.F. is member of steering cie of NovoNordisk evoke/evoke+. W.F. participated in advisory boards of Biogen MA Inc, Roche, and Eli Lilly; all funding is paid to her institution. W.F. is member of the steering committee of PAVE and Think Brain Health. W.F. was associate editor of *Alzheimer, Research & Therapy* in 2020/2021. WF is associate editor at *Brain*. The authors declare no other relevant competing interests. Author disclosures are available in the supporting information.

## CONSENT STATEMENT

This study was approved by the Massachusetts General Brigham Human Research Committee, which is the institutional review board for the Massachusetts General Hospital and Brigham and Women's Hospital. All cohorts contributing summary data were approved by their respective institutional review boards, some are registered at http://clinicaltrials.gov, and all participants provided informed consent.

## Supporting information



Supporting Information

Supporting Information

Supporting Information

## DELCODE study collaborators

**Table:**

Name	Affiliations
**Frederic Brosseron**	German Center for Neurodegenerative Diseases (DZNE), Bonn, Venusberg‐Campus 1, 53127 Bonn, Germany
**Katharina Buerger**	German Center for Neurodegenerative Diseases (DZNE, Munich), Feodor‐Lynen‐Strasse 17, 81377 Munich, Germany Institute for Stroke and Dementia Research (ISD), University Hospital, LMU Munich, Feodor‐Lynen‐Strasse 17, 81377 Munich, Germany
**Christoph Laske**	German Center for Neurodegenerative Diseases (DZNE), Tübingen, Germany Section for Dementia Research, Hertie Institute for Clinical Brain Research and Department of Psychiatry and Psychotherapy, University of Tübingen, Tübingen, Germany
**Robert Perneczky**	German Center for Neurodegenerative Diseases (DZNE, Munich), Feodor‐Lynen‐Strasse 17, 81377 Munich, Germany Department of Psychiatry and Psychotherapy, University Hospital, LMU Munich, Munich, Germany Munich Cluster for Systems Neurology (SyNergy) Munich, Munich, Germany Ageing Epidemiology Research Unit (AGE), School of Public Health, Imperial College London, London, UK
**Oliver Peters**	German Center for Neurodegenerative Diseases (DZNE), Berlin, Germany Charité – Universitätsmedizin Berlin, corporate member of Freie Universität Berlin and Humboldt‐Universität zu Berlin‐Institute of Psychiatry and Psychotherapy
**Joseph Priller**	German Center for Neurodegenerative Diseases (DZNE), Berlin, Germany Department of Psychiatry and Psychotherapy, Charité, Charitéplatz 1, 10117 Berlin, Germany School of Medicine, Technical University of Munich; Department of Psychiatry and Psychotherapy, Munich, Germany University of Edinburgh and UK DRI, Edinburgh, UK
**Alfredo Ramirez**	German Center for Neurodegenerative Diseases (DZNE), Bonn, Venusberg‐ Campus 1/99, 53127, Bonn, Germany Department of Cognitive Disorders and Old Age Psychiatry, University Hospital Bonn, Venusberg‐Campus 1, 53127, Bonn, Germany Excellence Cluster on Cellular Stress Responses in Aging‐Associated Diseases (CECAD), University of Cologne, Joseph‐Stelzmann‐Straße 26, 50931, Köln, Germany Division of Neurogenetics and Molecular Psychiatry, Department of Psychiatry and Psychotherapy, Faculty of Medicine and University Hospital Cologne, University of Cologne, Kerpener Straße 62, 50937, Köln, Germany Department of Psychiatry & Glenn Biggs Institute for Alzheimer's and Neurodegenerative Diseases, 7703 Floyd Curl Drive, MC 7835, San Antonio, TX 78229‐3900, USA
**Anja Schneider**	German Center for Neurodegenerative Diseases (DZNE), Bonn, Venusberg‐Campus 1, 53127 Bonn, Germany Department for Cognitive Disorders and Old Age Psychiatry, University Hospital Bonn, Bonn, Germany
**Annika Spottke**	German Center for Neurodegenerative Diseases (DZNE), Bonn, Venusberg‐Campus 1, 53127 Bonn, Germany Department of Neurology, University of Bonn, Venusberg‐Campus 1, 53127 Bonn, Germany
**Stefan Teipel**	German Center for Neurodegenerative Diseases (DZNE), Rostock, Germany Department of Psychosomatic Medicine, Rostock University Medical Center, Gehlsheimer Str. 20, 18147 Rostock
**Jens Wiltfang**	German Center for Neurodegenerative Diseases (DZNE), Goettingen, Germany Department of Psychiatry and Psychotherapy, University Medical Center Goettingen, University of Goettingen, Von‐Siebold‐Str. 5, 37075 Goettingen Neurosciences and Signaling Group, Institute of Biomedicine (iBiMED), Department of Medical Sciences, University of Aveiro, Aveiro, Portugal
