# Supplementary material for: SCD‐plus features and AD biomarkers in cognitively unimpaired samples: A meta‐analytic approach for nine cohort studies
Source: Alzheimers Dement. 2025 Feb 22;21(5):e14307. doi: 10.1002/alz.14307 (PMC12079645; doi:10.1002/alz.14307)
Supplement: Supplementary file 1 — Supporting Information [file ALZ-21-e14307-s002.docx]

**Supplementary Materials**

**Table of contents**

[**Supplementary Table 1.** Sample size by SCD-plus features and Alzheimer’s disease biomarker (GOLD Standard approach) 2](#_Toc164356460)

[**Supplementary Table 2.** Details on SCD questionnaires available by cohorts 3](#_Toc164356461)

[**Supplementary Table 3.** Details on items used by cohorts and by methodological approach 4](#_Toc164356462)

[**Supplementary Table 4.** Percentages of participants with preclinical AD pathological changes who also endorsed at least one of the four SCD features examined (SCD+Aβ+ or SCD+Aβ+T+) and the corresponding frequency of SCD endorsement among participants with amyloid and/or tau status (SCD+ in participants Aβ+ or Aβ+T+) within each available cohort (GOLD standard approach, mean SCD-severity score >0). 9](#_Toc164356463)

[**Supplementary Table 5.** Association between SCD-plus features and both amyloid- and tau-biomarker after adjustment for T+ and Aβ+ respectively (GOLD Standard approach) 15](#_Toc164356464)

[**Supplementary Table 6.** Association between SCD-plus features and both amyloid- and tau-biomarker respectively (Multiple items approach) 17](#_Toc164356465)

[**Supplementary Figure 1.** Proportion of participants with abnormally elevated amyloid levels (Aβ+) combined with the endorsement of individual SCD-plus features (**A**), and corresponding proportion of participants endorsing SCD features in the presence/absence of amyloid pathology (**B**; GOLD Standard approach). 11](#_Toc164356466)

[**Supplementary Figure 2.** Proportion of participants with abnormally elevated amyloid and tau levels (Aβ+T+) combined with the endorsement of individual SCD-plus features (**A**), and corresponding proportion of participants endorsing SCD features in the presence/absence of AD pathology (**B**; GOLD Standard approach). 12](#_Toc164356467)

[**Supplementary Figure 3. Association between self-reported SCD-plus features and amyloid levels (GOLD Standard approach). A**, Subjective memory decline (SMD). **B**, Associated concern/worry. **C**, Feeling of worse performances than peers of the same age. **D**, Onset of the subjective cognitive decline within the last 5 years. 13](#_Toc164356468)

[**Supplementary Figure 4. Association between self-reported SCD-plus features and tau levels (GOLD Standard approach). A**, Subjective memory decline (SMD). **B**, Associated concern/worry. **C**, Feeling of worse performances than peers of the same age. **D**, Onset of the subjective cognitive decline within the last 5 years. 14](#_Toc164356469)

# Supplementary Table 1. Sample size by SCD-*plus* features and Alzheimer’s disease biomarker (GOLD Standard approach)

|  | | **A4** | **ADNI** | **AIBL** | **DELCODE** | **HABS** | **IMAP+** | **SCIENCe** | **VMAP** | **WRAP** | **Total** |
| --- | --- | --- | --- | --- | --- | --- | --- | --- | --- | --- | --- |
| **Total** | **Aβ** | N_CU-PET_=4492 | N_CU-PET_ =275  N_CU-CSF_ =268 | N_CU-PET_=768 | N_CU-CSF_ =138 N_SCD-CSF_=211 | N_CU-PET_ =352 | N_CU-PET_ =56 N_SCD-PET_ =24 | N_SCD-PET_ =59  N_SCD-CSF_ =95 | N_CU-CSF_ =82 | N_CU-CSF_ =238 | N=7058  (389 SCD) |
|  | **Tau** | N_CU-PET_=447 | N_CU-PET_ =431  N_CU-CSF_ =268 |  | N_CU-CSF_=138 N_SCD-CSF_=211 | N_CU-PET_ =260 |  | N_SCD-CSF_=156 | N_CU-CSF_ =82 | N_CU-CSF_ =239 | N=2232  (367 SCD) |
|  | **Both** | N_CU-PET_=446 | N_CU-PET_ =275  N_CU-CSF_ =268 |  | N_CU-CSF_=138 N_SCD-CSF_=211 | N_CU-PET_ =260 |  | N_SCD-Both_=153 | N_CU-CSF_ =82 | N_CU-CSF_ =238 | N=2071  (364 SCD) |
| **SMD** | **Aβ** | N_CU-PET_=4492 | N_CU-PET_ =273  N_CU-CSF_ =222 | N_CU-PET_=768 | N_CU-CSF_ =138 N_SCD-CSF_=211 | N_CU-PET_ =352 | N_CU-PET_ =56 N_SCD-PET_ =24 | N_SCD-PET_ =57  N_SCD-CSF_ =94 | N_CU-CSF_ =82 | N_CU-CSF_ =238 | N=7007  (386 SCD) |
|  | **Tau** | N_CU-PET_=447 | N_CU-PET_ =429  N_CU-CSF_ =222 |  | N_CU-CSF_=138 N_SCD-CSF_=211 | N_CU-PET_ =257 |  | N_SCD-CSF_=153 | N_CU-CSF_ =82 | N_CU-CSF_ =239 | N =2178 (364 SCD) |
|  | **Both** | N_CU-PET_=446 | N_CU-PET_ =273  N_CU-CSF_ =222 |  | N_CU-CSF_=138 N_SCD-CSF_=211 | N_CU-PET_ =257 |  | N_SCD-Both_=150 | N_CU-CSF_ =82 | N_CU-CSF_ =238 | N =2017 (361 SCD) |
| **Onset** | **Aβ** |  |  |  | N_CU-CSF_ =138 N_SCD-CSF_=211 |  | N_CU-PET_ =37 N_SCD-PET_ =21 |  | N_CU-CSF_ =82 |  | N =489 (232 SCD) |
|  | **Tau** |  |  |  | N_CU-CSF_ =138 N_SCD-CSF_=211 |  |  |  | N_CU-CSF_ =82 |  | N =431 (211 SCD) |
|  | **Both** |  |  |  | N_CU-CSF_ =138 N_SCD-CSF_=211 |  |  |  | N_CU-CSF_ =82 |  | N =431 (211 SCD) |
| **Peer** | **Aβ** |  | N_CU-PET_ =274  N_CU-CSF_ =268 | N_CU-PET_=763 | N_CU-CSF_ =138 N_SCD-CSF_=211 | N_CU-PET_ =350 | N_CU-PET_ =38 N_SCD-PET_=21 | N_SCD-PET_ =55  N_SCD-CSF_ =92 | N_CU-CSF_ =82 |  | N =2292 (379 SCD) |
|  | **Tau** |  | N_CU-PET_ =430  N_CU-CSF_ =268 |  | N_CU-CSF_ =138 N_SCD-CSF_=211 | N_CU-PET_ =259 |  | N_SCD-CSF_=148 | N_CU-CSF_ =82 |  | N =1536 (359 SCD) |
|  | **Both** |  | N_CU-PET_ =274  N_CU-CSF_ =268 |  | N_CU-CSF_ =138 N_SCD-CSF_=211 | N_CU-PET_ =259 |  | N_SCD-Both_=146 | N_CU-CSF_ =82 |  | N =1378 (357 SCD) |
| **Concern/Worry** | **Aβ** | N_CU-PET_=4489 | N_CU-PET_ =250  N_CU-CSF_ =166 |  | N_CU-CSF_ =135 N_SCD-CSF_=201 | N_CU-PET_ =352 |  |  | N_CU-CSF_ =82 | N_CU-CSF_ =238 | N =5913 (201 SCD) |
|  | **Tau** | N_CU-PET_=446 | N_CU-PET_ =390  N_CU-CSF_ =166 |  | N_CU-CSF_ =135 N_SCD-CSF_=201 | N_CU-PET_ =256 |  |  | N_CU-CSF_ =82 | N_CU-CSF_ =239 | N =1915 (201 SCD) |
|  | **Both** | N_CU-PET_=445 | N_CU-PET_=250  N_CU-CSF_ =166 |  | N_CU-CSF_ =135 N_SCD-CSF_=201 | N_CU-PET_ =256 |  |  | N_CU-CSF_ =82 | N_CU-CSF_ =238 | N =1773 (201 SCD) |

# Abbreviations: Aβ, amyloid; A4; Anti-Amyloid Treatment in Asymptomatic Alzheimer Disease [1, 2]; ADNI, Alzheimer’s Disease Neuroimaging Initiative [3, 4]; AIBL, Australian Imaging, Biomarker & Lifestyle Flagship Study of Ageing [5]; CSF, cerebrospinal fluid; CU, cognitively unimpaired older adults; DELCODE, DZNE Longitudinal Cognitive Impairment and Dementia Study [6]; HABS, Harvard Aging Brain Study [7]; IMAP+, Imagerie Multimodale de la maladie d'Alzheimer à un stade Précoce [8, 9]; PET, positron emission tomography; SCD, patients with subjective cognitive decline recruited from memory clinics; SCIENCe, Subjective Cognitive Impairment Cohort [10]; SMD, self-reported subjective memory decline; VMAP, Vanderbilt Memory and Aging Project [11]; WRAP, Wisconsin Registry for Alzheimer's Prevention [12].

# Supplementary Table 2. Details on SCD questionnaires available by cohorts

| **Cohort** | **A4** | **ADNI** | **AIBL** | **DELCODE** | **HABS** | **IMAP+** | **SCIENCe** | **VMAP** | **WRAP** |
| --- | --- | --- | --- | --- | --- | --- | --- | --- | --- |
| **Memory question** [5, 12] |  |  | 1 question |  |  | 3 questions |  |  | 2 questions |
| **Structured interview** [10, 13] |  |  |  | SCD-Interview | STIDA (7 questions) |  | 6 questions |  |  |
| **CCI** [14] |  | 20 items |  |  |  |  | 20 items |  |  |
| **CCQ** [11] |  |  |  |  |  |  |  | 57 items |  |
| **CDS** [15] |  |  |  |  |  | 39 items |  | 41 items |  |
| **CFI** [16] | 15 items |  |  |  |  |  |  |  |  |
| **Ecog** [17] |  | 39 items + 1 |  |  | 39 items + 1 |  |  | 39 items |  |
| **GDS-15** [18] |  | 1 item | 1 item |  | 1 item |  | 1 item |  |  |
| **MAC-Q** [19] |  |  | 6 items |  |  |  |  |  |  |
| **MFQ** [20] |  |  |  |  | 47 items |  |  | 33 items | 33 items |
| **SCF** [10] |  |  |  |  |  |  | 4 items |  |  |
| **Total number of items** | 15 | 60 | 8 | 4 | 94 | 42 | 31 | 184 | 36 |
| **Consensus items;** 6 coders classified items with regard to each SCD feature. The consensus chosen items correspond to items with 50% (3/6) coders agreement. | | | | | | | | | |
| **SMD** | 6 [1 used]* | 18 [8 used]* | 7 [1 used]* | 1 | 44 [5 used]* | 19 | 12 | 98 [5 used]* | 19 |
| **Onset** |  |  |  | 1 |  | 1 |  | 4 |  |
| **Peer** |  | 2 [1used]* | 1 | 1 | 1 | 1 | 2 | 2 |  |
| **Concern/Worry** | 1 | 1 |  | 1 | 3 |  |  | 2 | 1 |
| **Method use** | Gold Standard^†^ | Both available | Gold Standard^†^ | Gold Standard^†^ | Both available | Both available | Both available | Multiple Item^‡^ | Multiple Item^‡^ |

# * Although the questionnaires used in these cohorts contain several SMD/peer items, only part of them was used due to ease of access at the time of analysis [e.g., A4 CFI items] and/or more than 10% missing data [e.g., 27% for CCI in ADNI, 10-36% for MFQ and Ecog in HABS, 11% for MAC-Q in AIBL, for the different questionnaires available in VMAP]. ^†^ Gold Standard approach, members from each cohort can choose 1 item within the previous consensus chosen items that best reflects each SCD feature, an individual will be code as endorsing that features if they are affirmative to this specific item; ^‡^ Multiple item approach, an individual has to be affirmative on any 1 [1-5 items available], 2 [6-10 items available], 3 [11-15 items available], 4 [16-20 items available], or 9 [41-45 items available] items to be considered to be endorsing the SCD-*plus* features. When multiple item and gold standard where available, primary analyses were done with gold standard approach. Abbreviations: A4; Anti-Amyloid Treatment in Asymptomatic Alzheimer Disease [1, 2]; ADNI, Alzheimer’s Disease Neuroimaging Initiative [3, 4]; AIBL, Australian Imaging, Biomarker & Lifestyle Flagship Study of Ageing [5]; CCI, cognitive change index; CCQ, cognitive change questionnaire; CDS, cognitive difficulties scale; CFI, cognitive function instrument; DELCODE, DZNE Longitudinal Cognitive Impairment and Dementia Study [6]; Ecog, everyday cognition questionnaire; GDS-15, short-form geriatric depression scale; HABS, Harvard Aging Brain Study [7]; IMAP+, Imagerie Multimodale de la maladie d'Alzheimer à un stade Précoce [8, 9]; MAC-Q, memory complaint questionnaire; SCF, subjective cognitive functioning; SCIENCe, Subjective Cognitive Impairment Cohort [10]; SMD, self-reported subjective memory decline; STIDA, structured telephone interview for dementia assessment; VMAP, Vanderbilt Memory and Aging Project [11]; WRAP, Wisconsin Registry for Alzheimer's Prevention [12].

# Supplementary Table 3. Details on items used by cohorts and by methodological approach

| **Cohort** | **Items** |
| --- | --- |
| **A4**  [gold standard only] | **SMD [individuals who answer “yes” are considered to endorse SMD]**   1. [CFI-15, yes/no/maybe^†^] Compared to 1 year ago, do you feel that your memory has declined substantially?   **Associated concern/worry [individuals who answer “yes” are considered to endorse worries]**   1. [CFI-15, yes/no] In the past year, have you seen a doctor about memory concerns?   ^†^*[CFI-15, yes/no or yes/no/maybe response, only GOLD Standard used for data accessibility]* |
| **ADNI**  [gold standard and multiple items] | **SMD [individuals who answer “little worse" and "consistently much worse" to the selected gold standard item, or to at least 2 of the 8 items for the multiple item approach, are considered to endorse SMD]**   1. [Ecog-39^‡^] Compared to 10 years ago, has there been any change in Remembering a few shopping items without a list. 2. [Ecog-39^‡^] Compared to 10 years ago, has there been any change in Remembering things that happened recently (such as recent outings, events in the news). **[GOLD standard chooses]** 3. [Ecog-39^‡^] Compared to 10 years ago, has there been any change in Recalling conversations a few days later. 4. [Ecog-39^‡^] Compared to 10 years ago, has there been any change in Remembering where she/he has placed objects. 5. [Ecog-39^‡^] Compared to 10 years ago, has there been any change in Repeating stories and/or questions. 6. [Ecog-39^‡^] Compared to 10 years ago, has there been any change in Remembering the current date or day of the week. 7. [Ecog-39^‡^] Compared to 10 years ago, has there been any change in Remembering he/she has already told someone something. 8. [Ecog-39^‡^] Compared to 10 years ago, has there been any change in Remembering appointments, meetings, or engagements.   **Peer comparison [individuals who answer “yes” are considered to endorse Peer]**   1. [GDS-15, yes/no] Do you feel you have more problems with memory than most?   **Associated concern/worry [individuals who answer “little worse" and "consistently much worse" are considered to endorse worries]**   1. [Ecog-39^‡^] Are you concerned that you have a memory or other thinking problem?   ^‡^*[Ecog, 4-pts Likert scale from “Better or no change” to “consistently much worse” and “I Don’t know”]* |
| **AIBL**  [gold standard only] | **SMD [individuals who answer “yes” are considered to endorse SMD]**   1. [General question, yes/no] Do you have difficulties with your memory?   **Peer comparison [individuals who answer “yes” are considered to endorse Peer]**   1. [GDS-15, yes/no] Do you feel you have more problems with memory than most? |
| **DELCODE**  [gold standard only] | **SMD [individuals who answer “yes” are considered to endorse SMD]**   1. [SCD-Interview, yes/no] do you feel like your memory has become worse?   **Onset [individuals that notice a decline within the past five years in at least one of the five domains explored are considered to endorse Onset]**   1. [SCD-Interview] How long ago did you start to notice the decline? (Within the last six months, between six months and two years ago, between two and five years ago, more than five years ago)   **Peer comparison [individuals that notice worse performances in at least one of the five domains explored are considered to endorse Peer]**   1. [SCD-Interview, yes/no] Compared to other people of your age, would you say that your performance is worse?   **Associated concern/worry [individuals worried in at least one of the five domains explored re considered to endorse worries]**   1. [SCD-Interview, yes/no] Does this worry you?   *[SCD-Interview: All interviews were administered by the study physician and lasted approximately 5 minutes. The Interview obtained specific information about complaints in five cognitive domains (memory, language, planning, attention, others). For each domain the physician asked the patient if he/she has noticed any worsening in function (e.g., “do you feel like your memory has become worse”). If the participant answers this question with yes, the physician added more in-depth questions about the domain to assess SCD-plus features, i.e., specific questions about associated worries (“Does this worry you?”), the beginning (“How long ago did you start to notice the decline?” and, the performance in comparison to peers (“Compared to other people of your age, would you say that your performance is worse?”). In addition, the semi-structured interview was administered with a relative of the participant to obtain information on confirmation of the participant’s perceived decline per cognitive domain]* |
| **HABS**  [gold standard and multiple items] | **SMD [individuals who answer “yes" to the selected gold standard item, or to at least 1 of the 5 items for the multiple item approach, are considered to endorse SMD]**   1. [STIDA, yes/no] Have you recently experienced a change in your ability to remember things? **[GOLD standard chooses]** 2. [STIDA, yes/no] Do you have more trouble than usual remembering a short list of items, such as a shopping list? 3. [STIDA, yes/no] Do you have trouble remembering things from one second to the next? 4. [STIDA, yes/no] Do you have much more trouble than usual remembering recent events? 5. [STIDA, yes/no] Do you have more trouble than usual following a group conversation or plot in a TV program due to your memory?   **Peer comparison [individuals who answer “yes” are considered to endorse Peer]**   1. [GDS-15, yes/no] Do you feel you have more problems with memory than most?   **Associated concern/worry [individuals who answer “little worse" and "consistently much worse" to the selected gold standard item, or endorse at least 1 of the 3 items for the multiple item approach (score ≥5 for MFQ items), are considered to endorse worries]**   1. [Ecog-39, yes/no^‡^] Are you concerned that you have a memory or other thinking problem? **[GOLD standard chooses]** 2. [MFQ-47^§^] How often do these present as a problem for you? Appointments 3. [MFQ-47^§^] How often do these present as a problem for you? Things people tell you   ^§^*[MFQ, 7-pts Likert scale from “No change” to “Much worse”]* |
| **IMAP+**  [gold standard and multiple items] | **SMD [individuals who answer “often” or “very often” to the selected gold standard item, or to at least 4 of the 19 items for the multiple item approach, are considered to endorse SMD]**   1. [CDS-39^¶^] I have trouble recalling frequently used phone numbers. 2. [CDS-39^¶^] I put down things (glasses, keys, wallet, purse, papers), and have trouble finding them. 3. [CDS-39^¶^] I need a written list when I do errands to avoid forgetting things. 4. [CDS-39^¶^] I forget appointments, dates, or classes. **[GOLD standard chooses]** 5. [CDS-39^¶^] I forget to return phone calls. 6. [CDS-39^¶^] I forget errands I planned to do on my way home. 7. [CDS-39^¶^] I have trouble recalling the names of people I know. 8. [CDS-39^¶^] I fail to recognize people I know. 9. [CDS-39^¶^] I have trouble thinking of the names of objects. 10. [CDS-39^¶^] I forget the names of people soon after being introduced. 11. [CDS-39^¶^] I forget steps in recipes I know well and have to look them up. 12. [CDS-39^¶^] I forget to button or zip my clothing. 13. [CDS-39^¶^] I need to check or double-check whether I locked the door, turned off the stove, ... 14. [CDS-39^¶^] I need to have instructions repeated several times. 15. [CDS-39^¶^] I forget right away what people say to me. 16. [CDS-39^¶^] When walking or riding, I forget how I’ve gotten from one point to another. 17. [CDS-39^¶^] I forget to pay bills, record checks, or mail letters. 18. [CDS-39^¶^] My mind goes blank at times. 19. [General question, yes/no] Have you experienced a change in your memory recently?   **Onset [individuals who answer <5 years are considered to endorse Onset]**   1. [General question] How long have you been feeling this change (6 months/one year/two years/>2 years, precise)?   **Peer comparison [individuals who answer “yes” are considered to endorse Peer]**   1. [General question, yes/no] Do you feel that your memory functions less well than that of people of your age?   ^¶^*[CDS, 5-pts Likert scale from “never” to “very often”]* |
| **SCIENCe**  [gold standard and multiple items] | **SMD [individuals who have a score <0 to the selected gold standard item, or endorse at least 3 of the 12 items for the multiple item approach (score** *≥***3 for CCI items, “yes” for the interview), are considered to endorse SMD]**   1. [CCI-20^#^] Recalling information when I really try 2. [CCI-20^#^] Remembering names and faces of new people I meet 3. [CCI-20^#^] Remembering things that have happened recently 4. [CCI-20^#^] Recalling conversations a few days later 5. [CCI-20^#^] Remembering where things are usually kept 6. [CCI-20^#^] Remembering new information told to me 7. [CCI-20^#^] Remembering where I placed familiar objects 8. [CCI-20^#^] Remembering what I intended to do 9. [CCI-20^#^] Remembering names of family members and friends 10. [CCI-20^#^] Remembering without notes and reminders 11. [SCF^††^] In the past year, have changes occurred in your memory **[GOLD standard chooses]** 12. [Interview, yes/no] Do you have problems with memory?   **Peer comparison [individuals who answer “yes” to the selected gold standard item, or endorse at least 1 of the 2 items for the multiple item approach (score** *≥***3 for CCI item) are considered to endorse Peer]**   1. [GDS-15, yes/no] Do you feel you have more problems with memory than most? **[GOLD standard chooses]** 2. [CCI-20^#^] Remembering things compared to my age group   ^#^*[CCI, 5-pts Likert scale from “No change” to “Much worse”]*  ^††^*[SCF, 7-pts Likert scale from “very strong improvement” to “very strong decline”]* |
| **VMAP**  [multiple items only] | **SMD [individuals who endorse at least 20 on the 98 items (respond “yes” to CCQ items, “often” or “very often” to CDS items, “little worse" and "consistently much worse" to Ecog items, and/or have a score >3 for MFQ items) are considered to endorse SMD]**   1. [Ecog-39^‡^] Compared to 10 years ago, has there been any change in Remembering things that happened recently (such as recent outings, events in the news). 2. [CCQ, yes/no] Do you think you have problems with your memory? 3. [CCQ, yes/no] Overall, do you feel you can remember things as well as you used to? 4. [CCQ, yes/no] Has your memory changed? 5. [CCQ, yes/no] Has your memory changed significantly? 6. [CCQ, yes/no] Do you have more trouble remembering things that have happened recently?   **Onset [individuals who answer “yes” on CCQ or “much worse” on the MFQ are considered to endorse Onset]:**   1. [CCQ, yes/no] Do you have complaints about your memory in the last 2 years? 2. [MFQ-47§] How is your memory compared to the way it was one year ago 3. [MFQ-47§] How is your memory compared to the way it was five years ago 4. [MFQ-47§] How is your memory compared to the way it was ten years ago   **Peer comparison [individuals who answer “yes” to at least 1 of the 2 items are considered to endorse Peer]**   1. [CCQ, yes/no] Do you consider your own memory to be worse than others that are your same age? 2. [CCQ, yes/no] Do you feel that you have more memory problems than most?   **Associated concern/worry [individuals who endorse at least 1 of the 3 items (respond “yes” to CCQ items, and/or “often” or “very often” to CDS items) are considered to endorse worries]**   1. [CDS-41^¶^] I worry about my memory or ability to pay attention. 2. [CCQ, yes/no] If you have memory difficulties, are they concerning you?   ^¶^*[CDS, 5-pts Likert scale from “never” to “very often”]*  ^‡^*[Ecog, 4-pts Likert scale from “Better or no change” to “consistently much worse” and “I Don’t know”]*  ^§^*[MFQ, 7-pts Likert scale from “Major Problems” to “No Problems”]* |
| **WRAP**  [multiple items] | **SMD [individuals who endorse at least 4 of the 19 items (respond “yes” to general question, and/or have a score ≥3 for MFQ items) are considered to endorse SMD]**   1. [General question, yes/no] Do you have difficulties with your memory? 2. [General question, 7-pts] Overall, how would you rate your memory? 3. [MFQ-33^§^] How would you rate your memory in terms of the kinds of problems that you have? 4. [MFQ-33^§^] How is your memory compared to the way it was one year ago 5. [MFQ-33^§^] How is your memory compared to the way it was five years ago 6. [MFQ-33^§^] How is your memory compared to the way it was ten years ago 7. [MFQ-33^§^] How is your memory compared to the way it was twenty years ago 8. [MFQ-33^§^] How often do these present as a problem for you? Names 9. [MFQ-33^§^] How often do these present as a problem for you? Faces 10. [MFQ-33^§^] How often do these present as a problem for you? Appointments 11. [MFQ-33^§^] How often do these present as a problem for you? Where you put things 12. [MFQ-33^§^] How often do these present as a problem for you? Phone numbers you've just checked 13. [MFQ-33^§^] How often do these present as a problem for you? Phone numbers you use frequently 14. [MFQ-33^§^] How often do these present as a problem for you? Things people tell you 15. [MFQ-33^§^] How often do these present as a problem for you? Personal dates (birthdays) 16. [MFQ-33^§^] How often do these present as a problem for you? Words 17. [MFQ-33^§^] How often do these present as a problem for you? Going to the store and forgetting what you wanted to buy 18. [MFQ-33^§^] How often do these present as a problem for you? Beginning to do something and forgetting what you were doing 19. [MFQ-33^§^] How often do these present as a problem for you? Knowing whether you 've already told someone something   **Associated concern/worry [individuals who have a score ≥3 for the MFQ item are considered to endorse worries]**   1. [MFQ-33^§^] When you actually forget in these situations, how serious of a problem do you consider the memory failure to be?   ^§^*[MFQ, 7-pts Likert scale from “No change” to “Much worse”]* |

# Abbreviations: A4; Anti-Amyloid Treatment in Asymptomatic Alzheimer Disease [1, 2]; ADNI, Alzheimer’s Disease Neuroimaging Initiative [3, 4]; AIBL, Australian Imaging, Biomarker & Lifestyle Flagship Study of Ageing [5]; CCI, cognitive change index; CCQ, cognitive change questionnaire; CDS, cognitive difficulties scale; CFI, cognitive function instrument; DELCODE, DZNE Longitudinal Cognitive Impairment and Dementia Study [6]; Ecog, everyday cognition questionnaire; GDS-15, short-form geriatric depression scale; HABS, Harvard Aging Brain Study [7]; IMAP+, Imagerie Multimodale de la maladie d'Alzheimer à un stade Précoce [8, 9]; MAC-Q, memory complaint questionnaire; SCF, subjective cognitive functioning; SCIENCe, Subjective Cognitive Impairment Cohort [10]; SMD, self-reported subjective memory decline; STIDA, structured telephone interview for dementia assessment; STIDA, structured telephone interview for dementia assessment; VMAP, Vanderbilt Memory and Aging Project [11]; WRAP, Wisconsin Registry for Alzheimer's Prevention [12].

**Supplementary Table 4.** Percentages of participants with preclinical AD pathological changes who also endorsed at least one of the four SCD-*plus* features examined (SCD+Aβ+ or SCD+Aβ+T+) and the corresponding frequency of SCD endorsement among participants with amyloid and/or tau status (SCD+ in participants Aβ+ or Aβ+T+) within each available cohort (GOLD standard approach, mean SCD-severity score >0).

|  | **Setting** | **Mod. Aβ** | **Aβ+** | **SCD+Aβ+** | **SCD+Aβ-** | **SCD+ % in Aβ+** | **SCD+ % in Aβ-** | **SCD+ % in Aβ+ vs. Aβ-** |
| --- | --- | --- | --- | --- | --- | --- | --- | --- |
| **A4** | CU | PET | 0.26 [0.25, 0.28] | 0.09 [0.09, 0.10] | 0.18 [0.17, 0.20] | 0.36 [0.33, 0.39] | 0.25 [0.23, 0.26] |  |
| **ADNI** | CU | PET | 0.36 [0.30, 0.41] | 0.19 [0.14, 0.24] | 0.29 [0.23, 0.34] | 0.53 [0.43, 0.63] | 0.45 [0.37, 0.52] |  |
| **ADNI** | CU | CSF | 0.38 [0.32, 0.43] | 0.17 [0.13, 0.22] | 0.24 [0.19, 0.29] | 0.46 [0.36, 0.55] | 0.38 [0.31, 0.46] |  |
| **AIBL** | CU | PET | 0.36 [0.33, 0.39] | 0.23 [0.20, 0.26] | 0.39 [0.36, 0.43] | 0.64 [0.58, 0.69] | 0.61 [0.57, 0.65] |  |
| **DELCODE** | CU | CSF | 0.25 [0.18, 0.33] | 0.15 [0.09, 0.21] | 0.32 [0.24, 0.40] | 0.60 [0.44, 0.76] | 0.43 [0.33, 0.52] |  |
| **DELCODE** | SCD | CSF | 0.39 [0.33, 0.46] | 0.38 [0.31, 0.44] | 0.60 [0.53, 0.66] | 0.96 [0.92, 1.00] | 0.98 [0.96, 1.01] |  |
| **HABS** | CU | PET | 0.23 [0.19, 0.28] | 0.12 [0.09, 0.16] | 0.29 [0.24, 0.33] | 0.54 [0.43, 0.64] | 0.37 [0.32, 0.43] |  |
| **IMAP+** | CU | PET | 0.23 [0.12, 0.34 | 0.09 [0.01, 0.16] | 0.16 [0.06, 0.26] | 0.38 [0.12, 0.65] | 0.21 [0.09, 0.33] |  |
| **IMAP+** | SCD | PET | 0.29 [0.11, 0.47] | 0.25 [0.08, 0.42] | 0.62 [0.43, 0.82] | 0.86 [0.60, 1.12] | 0.88 [0.73, 1.04] |  |
| **SCIENCe** | SCD | PET | 0.20 [0.10, 0.31] | 0.20 [0.10, 0.31] | 0.68 [0.56, 0.80] | 0.96 [0.86, 1.07] | 0.85 [0.75, 0.95] |  |
| **SCIENCe** | SCD | CSF | 0.35 [0.25, 0.44] | 0.32 [0.22, 0.41] | 0.59 [0.49, 0.69] | 0.91 [0.81, 1.01] | 0.90 [0.83, 0.98] |  |
| **VMAP** | CU | CSF | 0.16 [0.08, 0.24] | 0.07 [0.02, 0.15] | 0.28 [0.18, 0.38] | 0.46 [0.19, 0.73] | 0.33 [0.22, 0.44] |  |
| **WRAP** | CU | CSF | 0.25 [0.19, 0.30] | 0.11 [0.07, 0.15] | 0.27 [0.22, 0.33] | 0.44 [0.31, 0.57] | 0.36 [0.29, 0.43] |  |
| **Total** | | | 0.29 [0.25, 0.33]  I^2^=84.3% | 0.18 [0.13, 0.22]  I^2^=93.5% | 0.37 [0.29, 0.45]  I^2^=97.0% | 0.64 [0.47, 0.81]  I^2^=98.3% | 0.53 [0.32, 0.74]  I^2^=99.6% | Q_M_=0.62, p=0.43 |
| **CU** | | | 0.28 [0.24, 0.32]  I^2^=86.8% | 0.14 [0.10, 0.18]  I^2^=91.7% | 0.27 [0.21, 0.33]  I^2^=95.0% | 0.50 [0.40, 0.60]  I^2^=91.0% | 0.37 [0.26, 0.48]  I^2^=97.2% | *Q_M_=2.77, p=0.10* |
| **SCD** | | | 0.32 [0.23, 0.41]  I^2^=68.7% | 0.30 [0.21, 0.38]  I^2^=65.6% | 0.61 [0.56, 0.66]  I^2^=0.0% | 0.92 [0.85, 0.99]  I^2^=0.0% | 0.90 [0.82, 0.98]  I^2^=73.6% | Q_M_=0.17, p=0.68 |
| **CU vs SCD** | | | Q_M_=0.37, p=0.54 | **Q_M_=11.00, p<0.001** |  | **Q_M_=45.65, p<0.001** | **Q_M_=59.74, p<0.001** |  |
| **CSF** | | | 0.30 [0.22, 0.37]  I^2^=84.7% | 0.20 [0.11, 0.28]  I^2^=92.8% | 0.38 [0.25, 0.51]  I^2^=95.2% | 0.68 [0.44, 0.91]  I^2^=96.7% | 0.54 [0.25, 0.84]  I^2^=99.2% | Q_M_=0.47, p=0.49 |
| **PET** | | | 0.28 [0.24, 0.33]  I^2^=85.3% | 0.16 [0.10, 0.22]  I^2^=93.5% | 0.36 [0.25, 0.46]  I^2^=97.3% | 0.60 [0.43, 0.78]  I^2^=96.8% | 0.52 [0.34, 0.69]  I^2^=98.6% | Q_M_=0.49, p=0.48 |
| **CSF vs PET** | | | Q_M_=0.44, p=0.51 | Q_M_=0.49, p=0.48 |  | Q_M_=0.22, p=0.64 | Q_M_=0.03, p=0.87 |  |
|  |  |  |  |  |  |  |  |  |
|  | **Setting** | **Mod. Aβ/T** | **Aβ+T+** | **SCD+Aβ+T+** | **SCD+Aβ-T-** | **SCD+% in Aβ+T+** | **SCD+% in Aβ-T-** | **SCD+ % in Aβ+T+ vs. Aβ-T-** |
| **A4** | CU | PET/PET | 0.18 [0.15, 0.22] | 0.08 [0.06, 0.11] | 0.06 [0.04, 0.08] | 0.45 [0.34, 0.56] | 0.26 [0.18, 0.34] |  |
| **ADNI** | CU | PET/PET | 0.08 [0.05, 0.12] | 0.05 [0.03, 0.08] | 0.18 [0.14, 0.23] | 0.65 [0.46, 0.85] | 0.45 [0.38, 0.53] |  |
| **ADNI** | CU | CSF/CSF | 0.13 [0.09, 0.18] | 0.07 [0.04, 0.10] | 0.27 [0.22, 0.32] | 0.50 [0.34, 0.56] | 0.37 [0.28, 0.45] |  |
| **AIBL** | CU |  |  |  |  |  |  |  |
| **DELCODE** | CU | CSF/CSF | 0.04 [0.01, 0.08] | 0.02 [-0.00, 0.05] | 0.31 [0.23, 0.39] | 0.50 [0.10, 0.90] | 0.43 [0.34, 0.53] |  |
| **DELCODE** | SCD | CSF/CSF | 0.12 [0.08, 0.17] | 0.11 [0.07, 0.15] | 0.56 [0.50, 0.63] | 0.88 [0.76, 1.01] | 0.98 [0.96, 1.01] |  |
| **HABS** | CU | PET/PET | 0.10 [0.06, 0.13] | 0.05 [0.03, 0.08] | 0.23 [0.18, 0.28] | 0.56 [0.37, 0.75] | 0.33 [0.26, 0.40] |  |
| **IMAP+** | CU |  |  |  |  |  |  |  |
| **IMAP+** | SCD |  |  |  |  |  |  |  |
| **SCIENCe** | SCD |  |  |  |  |  |  |  |
| **SCIENCe** | SCD | CSF/CSF | 0.15 [0.08, 0.22] | 0.14 [0.07, 0.21] | 0.40 [0.31, 0.50] | 0.93 [0.79,1.06] | 0.95 [0.88,1.02] |  |
| **VMAP** | CU | CSF/CSF | 0.06 [0.01, 0.11] | 0.04 [-0.00, 0.08] | 0.17 [0.09, 0.25] | 0.60 [0.17, 1.03] | 0.28 [0.16, 0.40] |  |
| **WRAP** | CU | CSF/CSF | 0.12 [0.08, 0.16] | 0.05 [0.03, 0.08] | 0.24 [0.19, 0.30] | 0.45 [0.27, 0.63] | 0.35 [0.28, 0.42] |  |
| **Total** | | | 0.11 [0.08, 0.14]  I^2^=80.2% | 0.06 [0.04, 0.08]  I^2^=67.4% | 0.27 [0.17, 0.37]  I^2^=97.2% | 0.62 [0.47, 0.77]  I^2^=84.9% | 0.48 [0.23, 0.74]  I^2^=99.2% | Q_M_=0.83, p=0.36 |
| **CU** | | | 0.10 [0.07, 0.14]  I^2^=85.5% | 0.05 [0.04, 0.07]  I^2^=54.2% | 0.21 [0.13, 0.29]  I^2^=95.1% | 0.51 [0.44, 0.59]  I^2^=0.0% | 0.36 [0.31, 0.42]  I^2^=61.9% | **Q_M_=10.07, p=0.002** |
| **SCD** | | | 0.13 [0.09, 0.17]  I^2^=0.0% | 0.12 [0.08, 0.15]  I^2^=0.0% | 0.49 [0.33, 0.64]  I^2^=85.4% | 0.91 [0.82, 1.00]  I^2^=0.0% | 0.97 [0.94, 1.00]  I^2^=0.0% | Q_M_=1.48, p=0.22 |
| **CU vs SCD** | | | Q_M_=0.25, p=0.62 | **Q_M_=9.92, p=0.002** |  | **Q_M_=42.41, p<0.001** | **Q_M_=360.69, p<0.001** |  |
| **CSF** | | | 0.10 [0.07, 0.14]  I^2^=74.1% | 0.06 [-0.02, 0.15]  I^2^=91.9% | 0.44 [0.19, 0.69]  I^2^=95.7% | 0.78 [0.41, 1.16]  I^2^=69.2% | 0.68 [0.14, 1.22]  I^2^=99.1% | Q_M_=0.10, p=0.75 |
| **PET** | | | 0.12 [0.06, 0.18]  I^2^=89.2% | 0.06 [0.05, 0.08]  I^2^=37.7% | 0.22 [0.13, 0.31]  I^2^=95.4% | 0.59 [0.43, 0.75]  I^2^=82.6% | 0.42 [0.21, 0.62]  I^2^=97.7% | Q_M_=1.70, p=0.19 |
| **CSF vs PET** | | | Q_M_=0.44, p=0.51 | Q_M_=0.00, p=0.99 |  | Q_M_=0.89, p=0.35 | Q_M_=0.79, p=0.37 |  |

Abbreviations: Aβ-, amyloid-negative; Aβ+ amyloid-positive; Concern, presence of an associated SCD concern/worry; CSF, cerebrospinal fluid; CU, cognitively unimpaired older adults recruited from the community; I^2^, heterogeneity; NA, not available; Onset, onset of the subjective cognitive decline within the past 5 years; Peer, feeling of worse performances than peers of the same age; PET, Positron emission tomography; Q_M_, test of moderators; SCD, patients with subjective cognitive decline recruited from memory clinics; SCD+, endorsement of SCD features; SMD, self-reported subjective memory decline; T-, tau-negative; T+, tau-positive

**Supplementary Figure 1.** Proportion of participants with abnormally elevated amyloid levels (Aβ+) combined with the endorsement of individual SCD-*plus* features (**A**), and corresponding proportion of participants endorsing SCD features in the presence/absence of amyloid pathology (**B**; GOLD Standard approach).


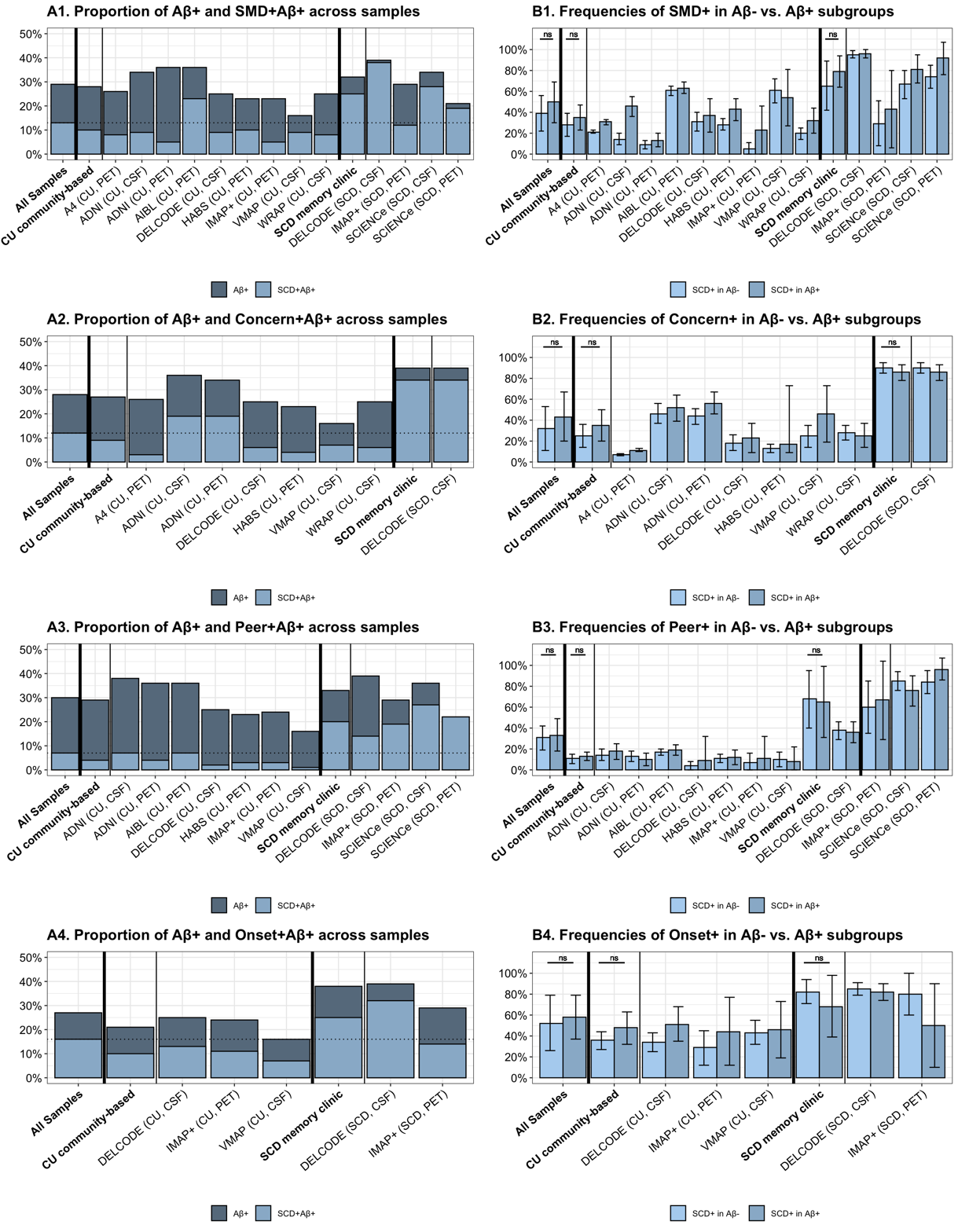


Abbreviations: Aβ-, amyloid-negative; Aβ+ amyloid-positive; Concern+, presence of an associated SCD concern/worry; CSF, cerebrospinal fluid; CU, cognitively unimpaired older adults recruited from the community; I^2^, heterogeneity; NA, not available; Onset+, onset of the subjective cognitive decline within the past 5 years; Peer+, feeling of worse performances than peers of the same age; ns, not significant p>0.10; PET, Positron emission tomography; Q_M_, test of moderators; SCD, patients with subjective cognitive decline recruited from memory clinics; SCD+, endorsement of SCD features; SMD+, presence of a self-reported subjective memory decline

**Supplementary Figure 2.** Proportion of participants with abnormally elevated amyloid and tau levels (Aβ+T+) combined with the endorsement of individual SCD-*plus* features (**A**), and corresponding proportion of participants endorsing SCD features in the presence/absence of AD pathology (**B**; GOLD Standard approach).


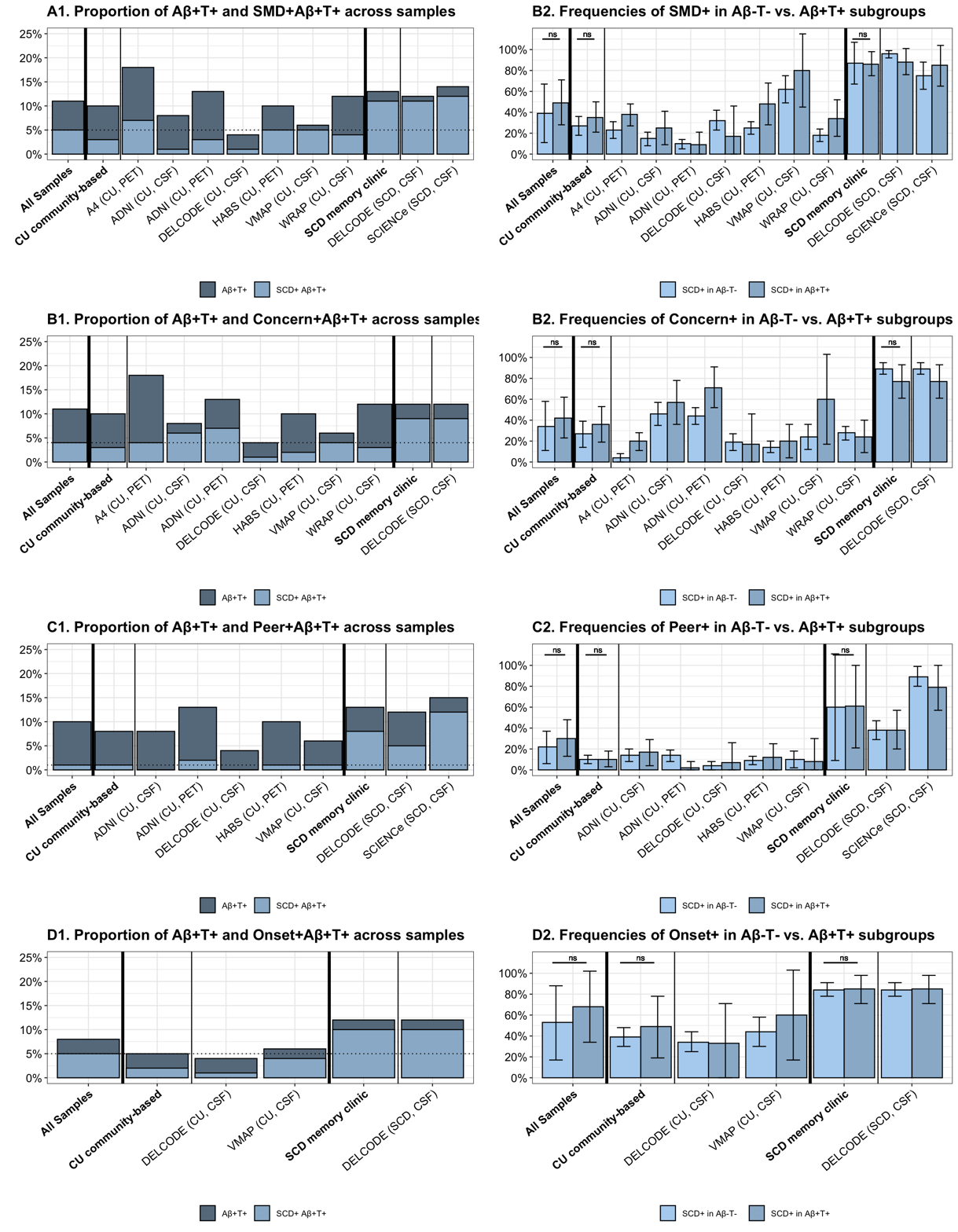


Abbreviations: Aβ-T-, amyloid- and tau-negative; Aβ+T+, amyloid- and tau-positive; Concern+, presence of an associated SCD concern/worry; CSF, cerebrospinal fluid; CU, cognitively unimpaired older adults recruited from the community; I^2^, heterogeneity; NA, not available; Onset+, onset of the subjective cognitive decline within the past 5 years; Peer+, feeling of worse performances than peers of the same age; ns, not significant p>0.10; PET, Positron emission tomography; Q_M_, test of moderators; SCD, patients with subjective cognitive decline recruited from memory clinics; SCD+, endorsement of SCD features; SMD+, presence of a self-reported subjective memory decline


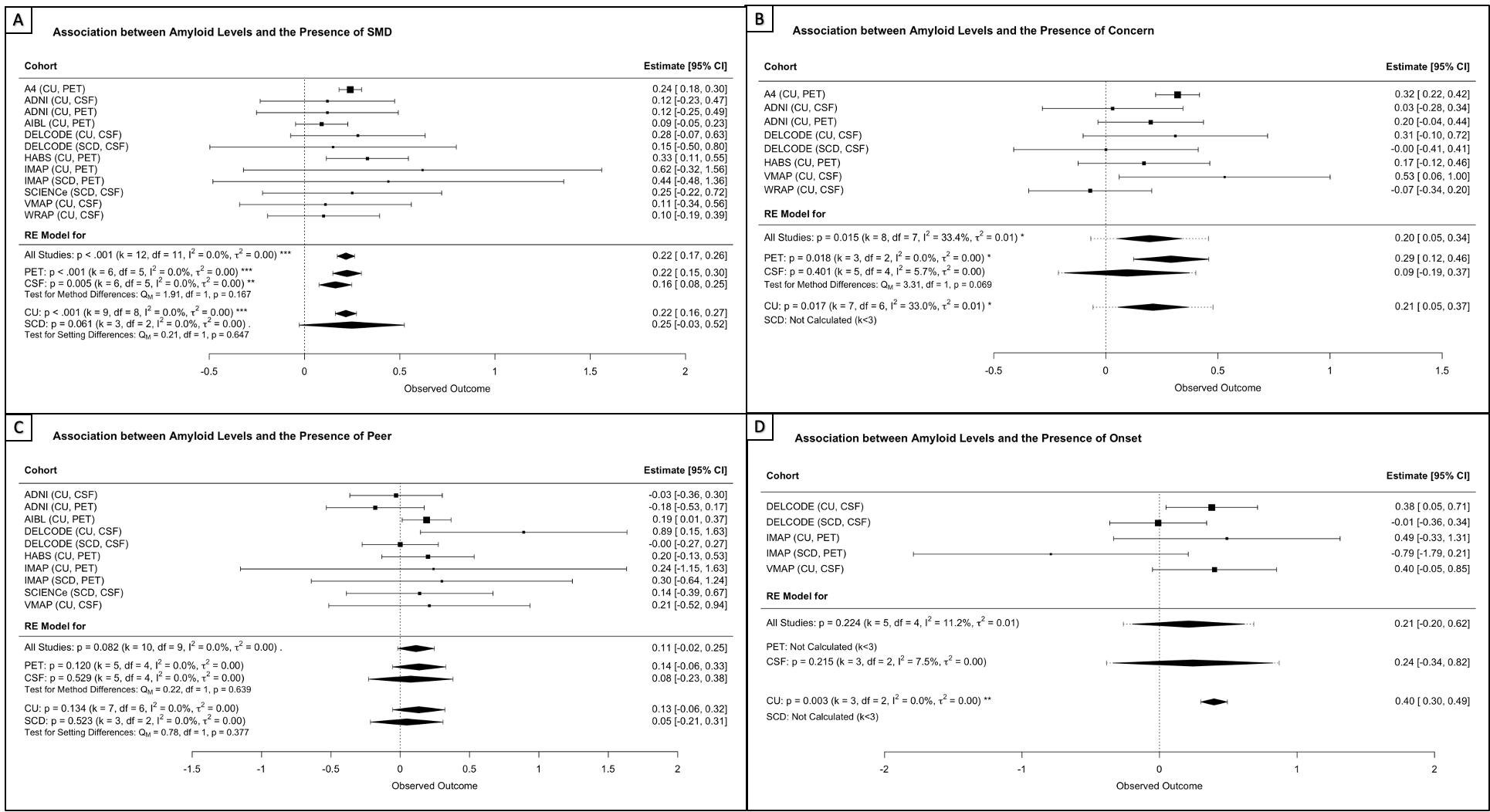


**Supplementary Figure 3. Association between self-reported SCD-*plus* features and amyloid levels (GOLD Standard approach). A**, Subjective memory decline (SMD). **B**, Associated concern/worry. **C**, Feeling of worse performances than peers of the same age. **D**, Onset of the subjective cognitive decline within the last 5 years.


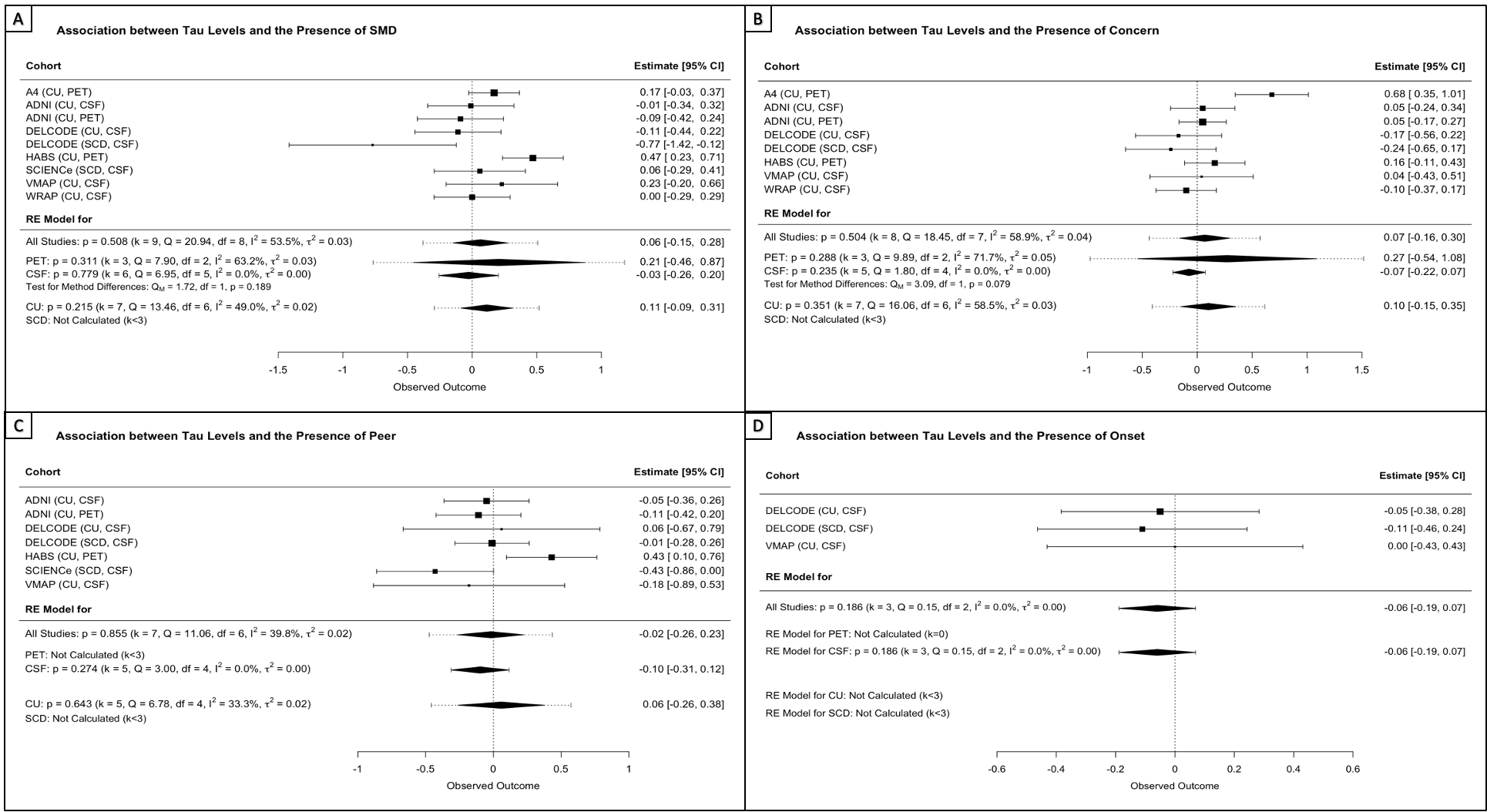


**Supplementary Figure 4. Association between self-reported SCD-*plus* features and tau levels (GOLD Standard approach). A**, Subjective memory decline (SMD). **B**, Associated concern/worry. **C**, Feeling of worse performances than peers of the same age. **D**, Onset of the subjective cognitive decline within the last 5 years.

**Supplementary Table 5.** Association between SCD-*plus* features and both amyloid- and tau-biomarker after adjustment for T+ and Aβ+ respectively (GOLD Standard approach)

|  | **Setting**  **[Modality Aβ/T]** | **Amyloid-positivity adj. tau-positivity** | **Amyloid-levels adj. tau-positivity** | **Tau-positivity adj. amyloid-positivity** | **Tau-levels adj. amyloid-positivity** |
| --- | --- | --- | --- | --- | --- |
| **SMD** | | | | | |
| **A4** | CU [PET/PET] | 0.17 [-0.34, 0.68] | 0.03 [-0.17, 0.23] | 0.49 [0.00, 0.98] | 0.15 [-0.05, 0.35] |
| **ADNI** | CU [PET/PET] | 0.48 [-0.32, 1.28] | 0.18 [-0.19, 0.55] | 1.12 [-2.69, 0.45] | -0.08 [-0.55, 0.39] |
| **ADNI** | CU [CSF/CSF] | 0.72 [-0.01, 1.45] | 0.12 [-0.23, 0.47] | 0.04 [-0.78, 0.86] | -0.05 [-0.38, 0.28] |
| **DELCODE** | CU [CSF/CSF] | 0.33 [-0.53, 1.19] | 0.35 [-0.00, 0.70] | -2.08 [-4.35, 0.19] | -0.13 [-0.46, 0.20] |
| **DELCODE** | SCD [CSF/CSF] | 1.06 [-0.59, 2.71] | 0.46 [-0.15, 1.07] | -2.33 [-3.94, -0.72] | -0.82 [-1.39, -0.25] |
| **HABS** | CU [PET/PET] | 0.25 [-0.44, 0.94] | 0.11 [-0.13, 0.35] | 0.39 [-0.34, 1.12] | 0.39 [0.15, 0.63] |
| **SCIENCe** | SCD [PET/CSF] | 1.95 [-0.64, 4.54] |  |  |  |
| **SCIENCe** | SCD [CSF/CSF] | 0.24 [-0.86, 1.34] | 0.26 [-0.21, 0.73] | 0.02 [-0.80, 0.84] | 0.01 [-0.34, 0.36] |
| **VMAP** | CU [CSF/CSF] | -0.36 [-1.59, 0.87] | 0.11 [-0.34, 0.56] | 0.05 [-0.97, 1.07] | 0.23 [-0.20, 0.66] |
| **WRAP** | CU [CSF/CSF] | 0.28 [-0.52, 1.08] | -0.02 [-0.29, 0.25] | 0.45 [-0.43, 1.33] | -0.11 [-0.36, 0.14] |
| **Total** | | 0.34 [0.11, 0.57]  **p=0.009**; I^2^=0.0% | 0.11 [0.02, 0.21]  **p=0.03**; I^2^=0.0% | 0.03 [-0.56, 0.62]  p=0.91; I^2^=29.7% | 0.01 [-0.21, 0.23]  p=0.91; I^2^=57.2% |
| **Concern** | | | | | |
| **A4** | CU [PET/PET] | 0.50 [-0.44, 1.44] | 0.38 [0.07, 0.69] | 1.45 [0.74, 2.16] | 0.62 [0.31, 0.93] |
| **ADNI** | CU [PET/PET] | 0.51 [-0.04, 1.06] | 0.15 [-0.09, 0.39] | 0.57 [-0.23, 1.37] | -0.05 [-0.36, 0.26] |
| **ADNI** | CU [CSF/CSF] | 0.18 [-0.47, 0.83] | 0.02 [-0.29, 0.33] | 0.15 [-0.58, 0.88] | 0.04 [-0.25, 0.33] |
| **DELCODE** | CU [CSF/CSF] | 0.42 [-0.56, 1.40] | 0.34 [-0.07, 0.75] | -0.88 [-3.13, 1.37] | -0.19 [-0.58, 0.20] |
| **DELCODE** | SCD [CSF/CSF] | 0.10 [-0.86, 1.06] | 0.08 [-0.31, 0.47] | -0.61 [-1.75, 0.53] | -0.23 [-0.60, 0.14] |
| **HABS** | CU [PET/PET] | 0.78 [-0.04, 1.60] | 0.23 [-0.06, 0.52] | 0.14 [-0.66, 0.94] | 0.03 [-0.22, 0.28] |
| **VMAP** | CU [CSF/CSF] | 0.81 [-0.46, 2.08] | 0.53 [0.04, 1.02] | 0.02 [-1.12, 1.16] | 0.02 [-0.47, 0.51] |
| **WRAP** | CU [CSF/CSF] | -0.16 [-0.94, 0.62] | -0.07 [-0.32, 0.18] | 0.03 [-0.85, 0.91] | -0.08 [-0.32, 0.16] |
| **Total** | | 0.36 [0.10, 0.62]  **p=0.01**; I^2^=0.0% | 0.16 [0.01, 0.32]  **p=0.04**; I^2^=10.4% | 0.29 [-0.27, 0.84]  p=0.26; I^2^=46.1% | 0.03 [-0.19, 0.25]  p=0.76; I^2^=56.3% |
| **Peer** | | | | | |
| **ADNI** | CU [PET/PET] | 0.00 [-0.82, 0.82] | -0.11 [-0.46, 0.24] | -1.47 [-3.53, 0.59] | -0.17 [-0.62, 0.28] |
| **ADNI** | CU [CSF/CSF] | 0.25 [-0.44, 0.94] | -0.03 [-0.36, 0.30] | -0.04 [-0.082 0.74] | -0.07 [-0.38, 0.24] |
| **DELCODE** | CU [CSF/CSF] | 1.15 [-0.46, 2.76] | 0.93 [0.20, 1.66] |  | -0.01 [-0.74, 0.72] |
| **DELCODE** | SCD [CSF/CSF] | 0.09 [-0.56, 0.74] | -0.02 [-0.27, 0.23] | 0.05 [-0.79, 0.89] | -0.03 [-0.28, 0.22] |
| **HABS** | CU [PET/PET] | -0.66 [-1.80, 0.48] | -0.20 [-0.55, 0.15] | 0.83 [-0.13, 1.79] | 0.36 [0.05, 0.67] |
| **SCIENCe** | SCD [CSF/CSF] | -0.53 [-1.67, 0.61] | 0.15 [-0.40, 0.70] | -0.42 [-1.34, 0.50] | -0.46 [-0.89, -0.03] |
| **VMAP** | CU [CSF/CSF] | -0.30 [-2.55, 1.95] | 0.21 [-0.53, 0.95] | 0.33 [-2.15, 1.49] | -0.18 [-0.89, 0.53] |
| **Total** | | 0.03 [-0.36, 0.41]  p=0.88; I^2^=0.0% | -0.01 [-0.23, 0.21]  p=0.92; I^2^=0.0% | -0.01 [-0.59, 0.58]  p=0.98; I^2^=0.0% | -0.04 [-0.278, 0.20]  p=0.67 ; I^2^=37.5% |
|  | **Setting**  **[Modality Aβ/T]** | **Amyloid-positivity adj. tau-positivity** | **Amyloid-levels adj. tau-positivity** | **Tau-positivity adj. amyloid-positivity** | **Tau-levels adj. amyloid-positivity** |
| **Onset** | | | | | |
| **DELCODE** | CU [CSF/CSF] | 0.82 [-0.00, 1.64] | 0.40 [0.07, 0.73] | -0.57 [-2.10, 0.96] | -0.10 [-0.43, 0.23] |
| **DELCODE** | SCD [CSF/CSF] | -0.21 [-1.01, 0.59] | -0.06 [-0.39, 0.27] | 0.57 [-0.65, 1.79] | -0.08 [-0.41, 0.25] |
| **VMAP** | CU [CSF/CSF] | 0.22 [-1.03, 1.47] | 0.40 [-0.05, 0.85] | 0.05 [-0.99, 1.09] | -0.01 [-0.44, 0.42] |
| **Total** | | 0.28 [-1.13, 1.69]  p=0.48; I^2^=15.7% | 0.23 [-0.45, 0.90]  p=0.29; I^2^=34.1% | 0.09 [-1.16, 1.34]  p=0.78; I^2^=0.0% | -0.07 [-0.18, 0.03]  p=0.10; I^2^=0.0% |
| **Mean SCD-severity** | | | | | |
| **A4** | CU [PET/PET] | 0.18 [-0.30, 0.66] | 0.06 [-0.02, 0.14] | 0.75 [0.29, 1.21] | 0.13 [0.05, 0.21] |
| **ADNI** | CU [PET/PET] | 0.41 [-0.04, 0.85] | 0.03 [-0.07, 0.13] | 0.42 [-0.09, 0.93] | -0.04 [-0.18, 0.10] |
| **ADNI** | CU [CSF/CSF] | 0.29 [-0.15, 0.71] | 0.01 [-0.11, 0.13] | 0.42 [-0.27, 1.10] | 0.00 [-0.12, 0.12] |
| **DELCODE** | CU [CSF/CSF] | 0.80 [0.14, 1.46] | 0.23 [0.07, 0.39] | -0.20 [-1.61, 1.22] | -0.07 [-0.23, 0.09] |
| **DELCODE** | SCD [CSF/CSF] | 0.12 [-0.96, 1.21] | 0.02 [-0.10, 0.14] | -2.49 [-3.78, -1.21] | -0.09 [-0.21, 0.03] |
| **HABS** | CU [PET/PET] | 0.39 [-0.18, 0.96] | 0.04 [-0.06, 0.14] | 0.55 [-0.03, 1.13] | 0.15 [0.05, 0.25] |
| **SCIENCe** | SCD [CSF/CSF] | 0.14 [-1.02, 1.31] | 0.10 [-0.10, 0.30] | -1.15 [-2.26, -0.04] | -0.10 [-0.30, 0.10] |
| **VMAP** | CU [CSF/CSF] | 0.42 [-0.67, 1.51] | 0.16 [-0.06, 0.38] | 1.09 [0.09, 2.09] | 0.02 [-0.20, 0.24] |
| **WRAP** | CU [CSF/CSF] | 0.04 [-0.60, 0.69] | -0.03 [-0.15, 0.09] | 0.32 [-0.40, 1.03] | -0.05 [-0.17, 0.07] |
| **Total** | | 0.32 [0.17, 0.48]  **p=0.001**; I^2^=0.0% | 0.05 [-0.00, 0.10]  **p=0.05**; I^2^=0.0% | 0.54 [0.31, 0.78]  **p=0.001**; I^2^=0.0% | 0.01 [-0.07, 0.08]  p=0.82; I^2^=57.8% |

Abbreviations: Aβ-, amyloid-negative; Aβ+ amyloid-positive; CSF, cerebrospinal fluid; CU, cognitively unimpaired older adults recruited from the community; NA, not available; Onset, onset of the subjective cognitive decline within the past 5 years; Peer, feeling of worse performances than peers of the same age; PET, Positron emission tomography; SCD, patients with subjective cognitive decline recruited from memory clinics; SMD, self-reported subjective memory decline; T-, tau-negative; T+, tau-positive.

**Supplementary Table 6.** Association between SCD-*plus* features and both amyloid- and tau-biomarker respectively (Multiple items approach)

|  | **Setting**  **[Modality Aβ/T]** | **Amyloid-positivity** | **Amyloid-levels** | **Tau-positivity** | **Tau-levels** |
| --- | --- | --- | --- | --- | --- |
| **SMD** | | | | | |
| **A4** | CU [PET/PET] | 0.46 [0.30, 0.62] | 0.24 [0.18, 0.30] | 0.50 [0.01, 0.99] | 0.17 [-0.03, 0.37] |
| **ADNI** | CU [PET/PET] | 0.75 [0.16, 1.34] | 0.21 [-0.08, 0.50] | 0.27 [-0.38, 0.92] | -0.01 [-0.28, 0.26] |
| **ADNI** | CU [CSF/CSF] | 0.53 [-0.06, 1.12] | 0.19 [-0.08, 0.46] | -0.13 [-0.84, 0.58] | -0.04 [-0.26, 0.18] |
| **AIBL** | CU [PET/NA] | 0.06 [-0.25, 0.37] | 0.09 [-0.05, 0.23] |  |  |
| **DELCODE** | CU [CSF/CSF] | 0.20 [-0.64, 1.04] | 0.28 [-0.07, 0.63] | -1.79 [-3.97, 0.39] | -0.11 [-0.44, 0.22] |
| **DELCODE** | SCD [CSF/CSF] | 0.22 [-1.23, 1.67] | 0.15 [-0.50, 0.80] | -1.79 [-3.22, 0.36] | -0.77 [-1.42, -0.12] |
| **HABS** | CU [PET/PET] | 0.47 [-0.06, 1.00] | 0.29 [0.09, 0.49] | 0.71 [-0.02, 1.44] | 0.28 [0.04, 0.52] |
| **IMAP+** | CU [PET/NA] | 2.00 [0.57, 3.43] | 0.63 [0.00, 1.26] |  |  |
| **IMAP+** | SCD [CSF/NA] | -0.11 [2.03, 1.81] | 0.36 [-0.54, 1.26] |  |  |
| **SCIENCe** | SCD [PET/CSF] | -0.57 [-1.65, 0.51] |  |  |  |
| **SCIENCe** | SCD [CSF/CSF] | 0.86 [-0.94, 2.66] | -0.07 [-0.56, 0.42] | 0.15 [-0.65, 0.95] | 0.05 [-0.30, 0.40] |
| **VMAP** | CU [CSF/CSF] | -0.35 [-1.58, 0.88] | 0.11 [-0.34, 0.56] | 0.05 [-0.97, 1.07] | 0.23 [-0.20, 0.66] |
| **WRAP** | CU [CSF/CSF] | 0.49 [-0.22, 1.20] | 0.10 [-0.19, 0.39] | 0.64 [-0.12, 1.40] | 0.00 [-0.29, 0.29] |
| **Total** |  | 0.38 [0.18, 0.58]  **p=0.001**; I^2^=11.4% | 0.22 [0.17, 0.26]  **p<0.001**; I^2^=0.0% | 0.25 [-0.18, 0.68]  p=0.21; I^2^=2.3% | 0.06 [-0.09, 0.21]  p=0.41; I^2^=15.4% |
| **Concern** | | | | | |
| **A4** | CU [PET/PET] | 0.50 [0.28, 0.72] | 0.32 [0.22, 0.42] | 1.53 [0.82, 2.24] | 0.68 [0.35, 1.01] |
| **ADNI** | CU [PET/PET] | 0.20 [-0.45, 0.85] | 0.03 [-0.28, 0.34] | 0.19 [-0.54, 0.92] | 0.05 [-0.24, 0.34] |
| **ADNI** | CU [CSF/CSF] | 0.57 [0.02, 1.12] | 0.20 [-0.04, 0.44] | 0.75 [0.12, 1.38] | 0.05 [-0.17, 0.27] |
| **DELCODE** | CU [CSF/CSF] | 0.36 [-0.62, 1.34] | 0.31 [-0.10, 0.72] | -0.76 [-2.97, 1.45] | -0.17 [-0.56, 0.22] |
| **DELCODE** | SCD [CSF/CSF] | -0.09 [-0.99, 0.81] | -0.00 [-0.41, 0.41] | -0.56 [-1.64, 0.52] | -0.24 [-0.65, 0.17] |
| **HABS** | CU [PET/PET] | -0.10 [-0.65, 0.45] | -0.01 [-0.23, 0.21] | -0.07 [-0.87, 0.73] | -0.10 [-0.37, 0.17] |
| **VMAP** | CU [CSF/CSF] | 0.81 [-0.46, 2.08] | 0.53 [0.06, 1.00] | 0.07 [-1.05, 1.19] | 0.04 [-0.43, 0.51] |
| **WRAP** | CU [CSF/CSF] | -0.12 [-0.83, 0.59] | -0.07 [-0.34, 0.20] | -0.02 [-0.80, 0.76] | -0.10 [-0.37, 0.17] |
| **Total** |  | 0.32 [0.07, 0.57]  **p=0.019**; I^2^=15.8% | 0.16 [-0.00, 0.32]  **p=0.05**; I^2^=47.6% | 0.30 [-0.29, 0.88]  p=0.27; I^2^=56.2% | 0.03 [-0.20, 0.27]  p=0.76; I^2^=60.4% |
| **Peer** | | | | | |
| **ADNI** | CU [PET/PET] | 0.25 [-0.42, 0.92] | -0.03 [-0.36, 0.30] | 0.01 [-0.77, 0.79] | -0.05 [-0.36, 0.26] |
| **ADNI** | CU [CSF/CSF] | -0.10 [-0.90, 0.70] | -0.18 [-0.53, 0.17] | -0.70 [-1.92, 0.52] | -0.11 [-0.42, 0.20] |
| **AIBL** | CU [PET/NA] | 0.28 [-0.13, 0.69] | 0.19 [0.01, 0.37] |  |  |
| **DELCODE** | CU [CSF/CSF] | 1.09 [-0.54, 2.72] | 0.89 [0.15, 1.63] |  | 0.06 [-0.67, 0.79] |
| **DELCODE** | SCD [CSF/CSF] | 0.10 [-0.51, 0.71] | -0.00 [-0.27, 0.27] | 0.13 [-0.65, 0.91] | -0.01 [-0.28, 0.26] |
| **HABS** | CU [PET/PET] | 0.21 [-0.57, 0.99] | 0.20 [-0.13, 0.53] | 0.90 [-0.00, 1.80] | 0.43 [0.10, 0.76] |
|  | **Setting**  **[Modality Aβ/T]** | **Amyloid-positivity** | **Amyloid-levels** | **Tau-positivity** | **Tau-levels** |
| **IMAP+** | CU [PET/NA] | 0.33 [-2.24, 2.90] | 0.24 [-1.15, 1.63] |  |  |
| **IMAP+** | SCD [PET/NA] | 0.43 [-1.63, 2.49] | 0.30 [-0.64, 1.24] |  |  |
| **SCIENCe** | SCD [CSF/CSF] | -0.66 [-1.84, 0.52] | 0.14 [-0.41, 0.69] | -0.28 [-1.20, 0.64] | -0.36 [-0.79, 0.07] |
| **VMAP** | CU [CSF/CSF] | -0.34 [-2.59, 1.91] | 0.21 [-0.52, 0.94] | -0.33 [-2.15, 1.49] | -0.18 [-0.89, 0.53] |
| **Total** |  | 0.17 [-0.03, 0.37]  p=0.084; I^2^=0.0% | 0.11 [-0.02, 0.25]  p=0.083; I^2^=0.0% | 0.07 [-0.47, 0.60]  p=0.77 ; I^2^=0.0% | -0.01 [-0.24, 0.22]  p=0.93; I^2^=33.4% |
| **Onset** | | | | | |
| **DELCODE** | CU [CSF/CSF] | 0.77 [-0.03, 1.57] | 0.38 [0.05, 0.71] | -0.43 [-1.92, 1.06] | -0.05 [-0.38, 0.28] |
| **DELCODE** | SCD [CSF/CSF] | -0.13 [-0.91, 0.65] | -0.01 [-0.36, 0.34] | 0.41 [-0.75, 1.57] | -0.11 [-0.46, 0.24] |
| **IMAP+** | CU [PET/NA] | 0.55 [-1.06, 2.16] | 0.49 [-0.33, 1.31] |  |  |
| **IMAP+** | SCD [PET/NA] | -2.08 [-4.65, 0.49] | -0.79 [-1.79, 0.21] |  |  |
| **VMAP** | CU [CSF/CSF] | 0.22 [-1.03, 1.47] | 0.40 [-0.05, 0.85] | 0.06 [-0.98, 1.10] | 0.00 [-0.43, 0.43] |
| **Total** |  | 0.23 [-0.59, 1.06]  p=0.48; I^2^=2.0% | 0.21 [-0.20, 0.62]  p=0.22; I^2^=11.2% | 0.08 [-0.85, 1.01]  p=0.75; I^2^=0.0% | -0.06 [-0.19, 0.07]  p=0.19; I^2^=0.0% |
| **Mean SCD-severity** | | | | | |
| **A4** | CU [PET/PET] | 0.51 [0.37, 0.64] | 0.13 [0.11, 0.15] | 0.78 [0.32, 1.23] | 0.15 [0.05, 0.25] |
| **ADNI** | CU [PET/PET] | 0.45 [0.04, 0.85] | -0.03 [-0.15, 0.09] | 0.35 [-0.11, 0.81] | 0.01 [-0.11, 0.13] |
| **ADNI** | CU [CSF/CSF] | 0.39 [-0.03, 0.81] | 0.05 [-0.07, 0.17] | 0.46 [-0.03, 0.94] | 0.00 [-0.10, 0.10] |
| **AIBL** | CU [PET/NA] | 0.09 [-0.18, 0.36] | 0.07 [0.01, 0.13] |  |  |
| **DELCODE** | CU [CSF/CSF] | 0.75 [0.11, 1.40] | 0.21 [0.05, 0.37] | 0.15 [-1.20 1.49] | -0.05 [-0.21, 0.11] |
| **DELCODE** | SCD [CSF/CSF] | 0.12 [-0.96, 1.21] | -0.00 [-0.14, 0.14] | -2.49 [-3.68, -1.29] | -0.09 [-0.23, 0.05] |
| **HABS** | CU [PET/PET] | 0.02 [-0.46, 0.50] | 0.10 [0.00, 0.20] | 0.73 [0.10, 1.36] | 0.11 [-0.01, 0.23] |
| **IMAP+** | CU [PET/NA] | -2.11 [-3.97, -0.26] | 0.28 [0.01, 0.55] |  |  |
| **IMAP+** | SCD [PET/NA] | 0.96 [-0.30, 2.21] | -0.01 [-0.44, 0.42] |  |  |
| **SCIENCe** | SCD [CSF/CSF] | -0.51 [-1.56, 0.54] | -0.01 [-0.21, 0.19] | -0.67 [-1.48, 0.14] | -0.06 [-0.22, 0.10] |
| **VMAP** | CU [CSF/CSF] | 0.42 [-0.67, 1.51] | 0.16 [-0.06, 0.38] | 1.11 [0.12, 2.11] | 0.02 [-0.20, 0.24] |
| **WRAP** | CU [CSF/CSF] | 0.19 [-0.37, 0.76] | -0.00 [-0.12, 0.12] | 0.38 [-0.24, 1.01] | -0.03 [-0.15, 0.09] |
| **Total** |  | 0.33 [0.16, 0.51]  **p=0.002**; I^2^=27.9% | 0.08 [0.03, 0.13]  **p=0.003**; I^2^=41.1% | 0.46 [0.11, 0.82]  **p=0.018**; I^2^=17.5% | 0.02 [-0.05, 0.08]  p=0.58; I^2^=39.1% |

Abbreviations: Aβ-, amyloid-negative; Aβ+ amyloid-positive; CSF, cerebrospinal fluid; CU, cognitively unimpaired older adults recruited from the community; NA, not available; Onset, onset of the subjective cognitive decline within the past 5 years; Peer, feeling of worse performances than peers of the same age; PET, Positron emission tomography; SCD, patients with subjective cognitive decline recruited from memory clinics; SMD, self-reported subjective memory decline; T-, tau-negative; T+, tau-positive.

**References**

1. Sperling, R.A., et al., *The A4 study: stopping AD before symptoms begin?* Sci Transl Med, 2014. **6**(228): p. 228fs13.

2. Sperling, R.A., et al., *Association of Factors With Elevated Amyloid Burden in Clinically Normal Older Individuals.* JAMA Neurol, 2020. **77**(6): p. 735-745.

3. Petersen, R.C., et al., *Alzheimer's Disease Neuroimaging Initiative (ADNI): clinical characterization.* Neurology, 2010. **74**(3): p. 201-9.

4. Aisen, P.S., et al., *Clinical core of the Alzheimer's disease neuroimaging initiative: Progress and plans.* Alzheimer's & Dementia, 2010. **6**(3): p. 239-246.

5. Ellis, K.A., et al., *The Australian Imaging, Biomarkers and Lifestyle (AIBL) study of aging: methodology and baseline characteristics of 1112 individuals recruited for a longitudinal study of Alzheimer's disease.* Int Psychogeriatr, 2009. **21**(4): p. 672-87.

6. Jessen, F., et al., *Design and first baseline data of the DZNE multicenter observational study on predementia Alzheimer's disease (DELCODE).* Alzheimers Res Ther, 2018. **10**(1): p. 15.

7. Dagley, A., et al., *Harvard Aging Brain Study: Dataset and accessibility.* Neuroimage, 2017. **144**(Pt B): p. 255-258.

8. Chételat, G., et al., *Three-dimensional surface mapping of hippocampal atrophy progression from MCI to AD and over normal aging as assessed using voxel-based morphometry.* Neuropsychologia, 2008. **46**(6): p. 1721-31.

9. Kuhn, E., et al., *Cross-sectional and longitudinal characterization of SCD patients recruited from the community versus from a memory clinic: subjective cognitive decline, psychoaffective factors, cognitive performances, and atrophy progression over time.* Alzheimer's Research & Therapy, 2019. **11**(1): p. 1-16.

10. Slot, R.E.R., et al., *Subjective Cognitive Impairment Cohort (SCIENCe): study design and first results.* Alzheimer's Research & Therapy, 2018. **10**(1): p. 76.

11. Jefferson, A.L., et al., *The Vanderbilt Memory & Aging Project: Study Design and Baseline Cohort Overview.* J Alzheimers Dis, 2016. **52**(2): p. 539-59.

12. Johnson, S.C., et al., *The Wisconsin Registry for Alzheimer's Prevention: A review of findings and current directions.* Alzheimers Dement (Amst), 2018. **10**: p. 130-142.

13. Miebach, L., et al., *Which features of subjective cognitive decline are related to amyloid pathology? Findings from the DELCODE study.* Alzheimer's Research & Therapy, 2019. **11**(1): p. 66.

14. Rattanabannakit, C., et al., *The Cognitive Change Index as a Measure of Self and Informant Perception of Cognitive Decline: Relation to Neuropsychological Tests.* Journal of Alzheimer's disease: JAD, 2016. **51**(4): p. 1145-1155.

15. McNair, D. and R. Kahn, *Self-assessment of cognitive deficits.* Assessment in geriatric psychopharmacology, 1983. **137**: p. 143.

16. Amariglio, R.E., et al., *Tracking early decline in cognitive function in older individuals at risk for Alzheimer's disease dementia: the Alzheimer's Disease Cooperative Study Cognitive Function Instrument.* JAMA neurology, 2015. **72**(4): p. 446-454.

17. Farias, S.T., et al., *The measurement of everyday cognition (ECog): scale development and psychometric properties.* Neuropsychology, 2008. **22**(4): p. 531-44.

18. Sheikh, J.I. and J.A. Yesavage, *Geriatric Depression Scale (GDS): recent evidence and development of a shorter version.* Clinical Gerontologist: The Journal of Aging and Mental Health, 1986.

19. Crook, T.H., E.P. Feher, and G.J. Larrabee, *Assessment of memory complaint in age-associated memory impairment: the MAC-Q.* Int Psychogeriatr, 1992. **4**(2): p. 165-76.

20. Gilewski, M.J., E.M. Zelinski, and K.W. Schaie, *The Memory Functioning Questionnaire for assessment of memory complaints in adulthood and old age.* Psychol Aging, 1990. **5**(4): p. 482-90.
