# Supplementary material for: SCD‐plus features and AD biomarkers in cognitively unimpaired samples: A meta‐analytic approach for nine cohort studies
Source: Alzheimers Dement. 2025 Feb 22;21(5):e14307. doi: 10.1002/alz.14307 (PMC12079645; doi:10.1002/alz.14307)
Supplement: Supplementary file 2 — Supporting Information [file ALZ-21-e14307-s001.docx]

# Appendix

## DELCODE study collaborators (to be indexed in Pubmed)

| **Name** | **Affiliations** |
| --- | --- |
| **Frederic Brosseron** | German Center for Neurodegenerative Diseases (DZNE), Bonn, Venusberg-Campus 1, 53127 Bonn, Germany |
| **Katharina Buerger** | 1. German Center for Neurodegenerative Diseases (DZNE, Munich), Feodor-Lynen-Strasse 17, 81377 Munich, Germany  2. Institute for Stroke and Dementia Research (ISD), University Hospital, LMU Munich, Feodor-Lynen-Strasse 17, 81377 Munich, Germany |
| **Christoph Laske** | 1. German Center for Neurodegenerative Diseases (DZNE), Tübingen, Germany  2. Section for Dementia Research, Hertie Institute for Clinical Brain Research and Department of Psychiatry and Psychotherapy, University of Tübingen, Tübingen, Germany |
| **Robert Perneczky** | 1. German Center for Neurodegenerative Diseases (DZNE, Munich), Feodor-Lynen-Strasse 17, 81377 Munich, Germany  2. Department of Psychiatry and Psychotherapy, University Hospital, LMU Munich, Munich, Germany  3. Munich Cluster for Systems Neurology (SyNergy) Munich, Munich, Germany  4. Ageing Epidemiology Research Unit (AGE), School of Public Health, Imperial College London, London, UK |
| **Oliver Peters** | 1. German Center for Neurodegenerative Diseases (DZNE), Berlin, Germany  2. Charité – Universitätsmedizin Berlin, corporate member of Freie Universität Berlin and Humboldt-Universität zu Berlin-Institute of Psychiatry and Psychotherapy |
| **Joseph Priller** | 1. German Center for Neurodegenerative Diseases (DZNE), Berlin, Germany  2. Department of Psychiatry and Psychotherapy, Charité, Charitéplatz 1, 10117 Berlin, Germany  3. School of Medicine, Technical University of Munich; Department of Psychiatry and Psychotherapy, Munich, Germany  4. University of Edinburgh and UK DRI, Edinburgh, UK |
| **Alfredo Ramirez** | 1. German Center for Neurodegenerative Diseases (DZNE), Bonn, Venusberg- Campus 1/99, 53127, Bonn, Germany  2. Department of Cognitive Disorders and Old Age Psychiatry, University Hospital Bonn, Venusberg-Campus 1, 53127, Bonn, Germany  3. Excellence Cluster on Cellular Stress Responses in Aging-Associated Diseases (CECAD), University of Cologne, Joseph-Stelzmann-Straße 26, 50931, Köln, Germany  4. Division of Neurogenetics and Molecular Psychiatry, Department of Psychiatry and Psychotherapy, Faculty of Medicine and University Hospital Cologne, University of Cologne, Kerpener Straße 62, 50937, Köln, Germany  5. Department of Psychiatry & Glenn Biggs Institute for Alzheimer’s and Neurodegenerative Diseases, 7703 Floyd Curl Drive, MC 7835, San Antonio, TX 78229-3900, USA |
| **Anja Schneider** | 1. German Center for Neurodegenerative Diseases (DZNE), Bonn, Venusberg-Campus 1, 53127 Bonn, Germany  2. Department for Cognitive Disorders and Old Age Psychiatry, University Hospital Bonn, Bonn, Germany |
| **Annika Spottke** | 1. German Center for Neurodegenerative Diseases (DZNE), Bonn, Venusberg-Campus 1, 53127 Bonn, Germany  2. Department of Neurology, University of Bonn, Venusberg-Campus 1, 53127 Bonn, Germany |
| **Stefan Teipel** | 1. German Center for Neurodegenerative Diseases (DZNE), Rostock, Germany  2. Department of Psychosomatic Medicine, Rostock University Medical Center, Gehlsheimer Str. 20, 18147 Rostock |
| **Jens Wiltfang** | 1. German Center for Neurodegenerative Diseases (DZNE), Goettingen, Germany  2. Department of Psychiatry and Psychotherapy, University Medical Center Goettingen, University of Goettingen, Von-Siebold-Str. 5, 37075 Goettingen  3. Neurosciences and Signaling Group, Institute of Biomedicine (iBiMED), Department of Medical Sciences, University of Aveiro, Aveiro, Portugal |

Others members of the DELCODE study group mentioned below.

## ADNI coinvestigators

| **Name, Degree** | **Location** | **Contribution** |
| --- | --- | --- |
| Bret Borowski, RTR | Mayo Clinic | ADNI; Leadership and Infrastructure - MRI Core Leaders and Key Personnel |
| Gloria Chiang, MD | Cornell University | Past Investigator; ADNI investigators |
| Jonathan Drake, MD | Rhode Island Hospital | ADNI investigators |
| Juliet Fockler, | University of California, San Francisco | ADNI; Leadership and Infrastructure - Administrative Core Northern California Institute for Research & Education (NCIRE / The Vererans Health Research Institute) |
| Karen Blank, MD | Hartford Hospital, Olin Neuropsychiatry Research Center | Past Investigator; ADNI investigators |
| Kelly M. Makino, BS | University of Rochester Medical Center | Past Investigator; ADNI investigators |
| Riham El Khouli, MD | University of Kentucky | ADNI investigators |
| Aaron Ritter, MD | Cleveland Clinic Lou Ruvo Center for Brain Health | ADNI investigators |
| Abigail O’Connelll, MS, APRN, FNP-C | Roper St. Francis Healthcare | ADNI investigators |
| Adam Fleisher, MD | University of Southern California | ADNI investigators |
| Adam P. Mecca, MD, PhD | Yale University School of Medicine | ADNI investigators |
| Adrienne Kormos, | NCIRE / The Vererans Health Research Institute | ADNI; Leadership and Infrastructure - Administrative Core Northern California Institute for Research & Education (NCIRE / The Vererans Health Research Institute) |
| Aimee Pierce, MD | University of California Irvine IMIND | ADNI investigators |
| aimie Ziolkowski, MA, BS, TLLP | University of Michigan | ADNI investigators |
| Akiva Mintz, MD, PhD | Columbia University Medical Center | ADNI investigators, past investigator of Cleveland Clinic Lou Ruvo Center for Brain Health |
| Alan Lerner, MD | Case Western Reserve University | ADNI investigators |
| Alexander Knaack, MS | University of California, Davis | ADNI; Leadership and Infrastructure - MRI Core Leaders and Key Personnel |
| Alireza Atri, MD, PhD | Banner Sun Health Research Institute | ADNI investigators |
| Allan I. Levey, MD, PhD | Emory University | ADNI investigators |
| Allison Perrin, PhD | Banner Alzheimer's Institute | ADNI investigators |
| Allyson Rosen, PhD | Stanford University | Past Investigator; ADNI investigators |
| Amanda Smith, MD | University of South Florida: USF Health Byrd Alzheimer’s Institute | ADNI investigators |
| Anaztasia Ulysse, BA | New York University | ADNI investigators |
| Andrew E. Budson, MD | Boston University | Past Investigator; ADNI investigators |
| Andrew J. Saykin, PsyD | Indiana University School of Medicine | ADNI; Leadership and Infrastructure - Executive Committee, Genetics Core Leaders and Key Personnel (Core-PI) |
| Andrew Kertesz, MD | St. Joseph’s Health Care | Past Investigator; ADNI investigators |
| Angela Oliver, RN, BSN, MSG | Washington University, St. Louis | Past Investigator; ADNI investigators |
| Angelica Garcia, BS | Barrow Neurological Institute | ADNI investigators |
| Ann Marie Hake, MD | Indiana University | Past Investigator; ADNI investigators |
| Anna Burke, PhD | Banner Alzheimer's Institute | Past Investigator; ADNI investigators |
| Antero Sarrael, MD | Nathan Kline Institute | ADNI investigators |
| Anton P. Porsteinsson, MD | University of Rochester Medical Center | ADNI investigators |
| Arline Faustin, MD | New York University | ADNI investigators |
| Arthur W. Toga, PhD | University of Southern California | ADNI; Leadership and Infrastructure - Executive Committee, Informatics Core Leaders and Key Personnel (Core PI) |
| Arthur Wiliams, BS | Roper St. Francis Healthcare | ADNI investigators |
| Arvin Forghanian-Arani, PhD | Mayo Clinic | ADNI; Leadership and Infrastructure - MRI Core Leaders and Key Personnel |
| Ashley Lamb, MA | Baylor College of Medicine | ADNI investigators |
| Athena Lee, PhD | Cornell University | ADNI investigators |
| Balebail Ashok Raj, MD | University of South Florida: USF Health Byrd Alzheimer’s Institute | Past Investigator; ADNI investigators |
| Barton Lane, MD | Stanford University | Past Investigator; ADNI investigators |
| Beatriz Yanez, RN | University of California Irvine IMIND | ADNI investigators |
| Beau Ances, MD, PhD, MSc | Washington University, St. Louis | ADNI investigators |
| Benita Mudge, BS | U.B.C. Clinic for AD & Related Disorders | ADNI investigators |
| Betty Lind, BS | Oregon Health & Science University | ADNI investigators |
| Bojana Stefanovic, PhD | Sunnybrook Health Sciences, Ontario | ADNI investigators |
| Bonnie S. Goldstein, MS, NP | University of Rochester Medical Center | Past Investigator; ADNI investigators |
| Borna Bonakdarpour, MD | Northwestern University | Past Investigator; ADNI investigators |
| Brandy R. Matthews, MD | Indiana University | Past Investigator; ADNI investigators |
| Brendan Kelley, MD | University of Texas Southwestern Medical School | ADNI investigators , past investigator of Ohio State University |
| Brian R. Ott, MD | Rhode Island Hospital | ADNI investigators |
| Brigid Reynolds, NP | Georgetown University Medical Center | ADNI investigators |
| Brittanie Muse, MSPH | Northwestern University | ADNI investigators |
| Brittany Sloan, BA | University of Southern California | ADNI; Leadership and Infrastructure - Clinical Core leaders and key personnel |
| Bruce L. Miller, MD | University of California, San Francisco | ADNI investigators |
| Bryan M. Spann, DO, PhD | Banner Sun Health Research Institute | ADNI investigators, past investigator of Oregon Health & Science University |
| Caileigh Zimmerman, MS | University of Southern California | ADNI; Leadership and Infrastructure - Clinical Core leaders and key personnel |
| Carole Ho | Denali Therapeutics | ADNI; Leadership and Infrastructure - ADNI External Advisory Board (ESAB) |
| Cat Conti, BA | NCIRE / The Vererans Health Research Institute | ADNI; Leadership and Infrastructure - Administrative Core Northern California Institute for Research & Education (NCIRE / The Vererans Health Research Institute), Resource Allocation Review Committee |
| Chad Ward, | Mayo Clinic | ADNI; Leadership and Infrastructure - MRI Core Leaders and Key Personnel |
| Charles Bernick, MD, MPH | Cleveland Clinic Lou Ruvo Center for Brain Health | Past Investigator; ADNI investigators |
| Charles D. Smith, MD | University of Kentucky | ADNI investigators |
| Charles DeCarli, MD | University of California, Davis | ADNI; Leadership and Infrastructure - MRI Core Leaders and Key Personnel, Investigators site Sacramento |
| Chengshi Jin, PhD | University of California, San Francisco | ADNI; Leadership and Infrastructure - Administrative Core Northern California Institute for Research & Education (NCIRE / The Vererans Health Research Institute) |
| Chet Mathis, MD | University of Pittsburgh | ADNI; Leadership and Infrastructure - PET Core Leaders and Key Personnel |
| Chiadi Onyike, MD | Johns Hopkins University | ADNI investigators |
| Chris (Chinthaka) Heyn, BSC, PhD, MD, FRCPC | Sunnybrook Health Sciences, Ontario | ADNI investigators |
| Chris Hosein, MEd | McGill Univ., Montreal-Jewish General Hospital | ADNI investigators |
| Christine M. Belden, PsyD | Banner Sun Health Research Institute | ADNI investigators |
| Christopher H. van Dyck, MD | Yale University School of Medicine | ADNI investigators |
| Christopher M. Clark, MD | University of Pennsylvania | Past Investigator; ADNI investigators |
| Christopher Schwarz, PhD | Mayo Clinic | ADNI; Leadership and Infrastructure - MRI Core Leaders and Key Personnel |
| Chuang-Kuo Wu, MD, PhD | Northwestern University | Past Investigator; ADNI investigators |
| Clifford R. Jack, Jr., MD | Mayo Clinic, Rochester | ADNI; Leadership and Infrastructure - Executive Committee, MRI Core Leaders and Key Personnel |
| Colleen S. Albers, RN | Mayo Clinic, Rochester | ADNI investigators |
| Connie Brand, RN | University of Rochester Medical Center | Past Investigator; ADNI investigators |
| Curtis Tatsuoka, PhD | Case Western Reserve University | ADNI investigators |
| Cynthia M. Carlsson, MD, MS | University of Wisconsin | ADNI investigators |
| Dallas Veitch, PhD | NCIRE / The Vererans Health Research Institute | ADNI; Leadership and Infrastructure - Administrative Core Northern California Institute for Research & Education (NCIRE / The Vererans Health Research Institute) |
| Dana Mathews, MD, PhD | University of Texas Southwestern Medical School | Past Investigator; ADNI investigators |
| Daniel H.S. Silverman, MD, PhD | University of California, Los Angeles | ADNI investigators |
| Daniel Marson, JD, PhD | University of California – San Diego | Past Investigator; ADNI investigators |
| Danielle Harvey, PhD | University of California, Davis | ADNI; Leadership and Infrastructure - Biostatistics Core Leaders and Key Personnel, MRI Core Leaders and Key Personnel |
| David A. Wolk, MD | University of Pennsylvania | ADNI investigators |
| David Clark, MD | University of California – San Diego | Past Investigator; ADNI investigators |
| David Geldmacher, MD | University of Alabama - Birmingham | ADNI investigators |
| David Hart, MD | Albany Medical College | ADNI investigators |
| David Jones, MD | Mayo Clinic | ADNI; Leadership and Infrastructure - MRI Core Leaders and Key Personnel |
| David Knopman, MD | Mayo Clinic, Rochester | ADNI investigators |
| David Perry, MD | University of California, San Francisco | ADNI investigators |
| David Winkfield, BS | Washington University, St. Louis | ADNI investigators |
| Delwyn D. Miller, PharmD, MD | University of Iowa College of Medicine | ADNI investigators |
| Derek Flenniken, | NCIRE / The Vererans Health Research Institute | ADNI; Leadership and Infrastructure - Administrative Core Northern California Institute for Research & Education (NCIRE / The Vererans Health Research Institute) |
| Devon Gessert, BS | University of Southern California | ADNI; Leadership and Infrastructure - Clinical Core leaders and key personnel |
| Diana Kerwin, MD | Northwestern University | Past Investigator; ADNI investigators |
| Diana Truran Sacrey, | NCIRE / The Vererans Health Research Institute | ADNI; Leadership and Infrastructure - Administrative Core Northern California Institute for Research & Education (NCIRE / The Vererans Health Research Institute) |
| Dick Drost, MD | St. Joseph’s Health Care | Past Investigator; ADNI investigators |
| Donna M. Simpson, CRNP, MPH | University of Pittsburgh | ADNI investigators |
| Donna Masterman, MD | Biogen | ADNI; Leadership and Infrastructure - ADNI External Advisory Board (ESAB) |
| Donna Munic, PhD | Cleveland Clinic Lou Ruvo Center for Brain Health | Past Investigator; ADNI investigators |
| Douglas W. Scharre, MD | Ohio State University | ADNI investigators |
| Dr Rob Bartha, PhD | Parkwood Institute | ADNI investigators |
| Duygu Tosun-Turgut, PhD | University of California, San Francisco | ADNI; Leadership and Infrastructure - MRI Core Leaders and Key Personnel |
| Dzintra Celmins, MD | Albany Medical College | ADNI investigators |
| Earl A. Zimmerman, MD | Albany Medical College | ADNI investigators |
| Edmond Teng, MD, PhD | University of California, Los Angeles | Past Investigator; ADNI investigators |
| Edward Zamrini, MD | Banner Sun Health Research Institute | Past Investigator; ADNI investigators |
| Effie Mitsis, PhD | Mount Sinai School of Medicine | Past Investigator; ADNI investigators |
| Eliezer Masliah, MD | NIA | ADNI; Leadership and Infrastructure - ADNI External Advisory Board (ESAB) |
| Elizabeth Finger, MD | St. Joseph’s Health Care | ADNI investigators |
| Elizabeth Shaffer, BS | University of Southern California | ADNI; Leadership and Infrastructure - Clinical Core leaders and key personnel |
| Elizabeth Sosa, PhD | University of California Irvine IMIND | ADNI investigators |
| Ellen Kim, BA | U.B.C. Clinic for AD & Related Disorders | ADNI investigators |
| Ellen Woo, PhD | University of California, Los Angeles | Past Investigator; ADNI investigators |
| Emily Rogalski, PhD | Northwestern University | ADNI investigators |
| Eric M. Reiman, MD | Banner Alzheimer’s Institute | ADNI; Leadership and Infrastructure - PET Core Leaders and Key Personnel |
| Erin Franklin, MS | Washington University St. Louis | ADNI; Leadership and Infrastructure - Neuropathology Core Leaders and Key Personnel |
| Erin Householder, MS | Washington University St. Louis | ADNI; Leadership and Infrastructure - Neuropathology Core Leaders and Key Personnel |
| Evan Fletcher, PhD | University of California, Davis | ADNI; Leadership and Infrastructure - MRI Core Leaders and Key Personnel, Investigators site Sacramento |
| Evaristus Nwulia, MD | Howard University | ADNI investigators |
| Flavius D. Raslau, MD | University of Kentucky | ADNI investigators |
| Francine Parfitt, MSH, CCRC | Mayo Clinic, Jacksonville | ADNI investigators |
| Gaby Thai, MD | University of California Irvine IMIND | ADNI investigators |
| Gad A. Marshall, MD | Sunnybrook Health Sciences, Ontario | ADNI investigators |
| Garrett Miller, MS | University of Southern California | ADNI; Leadership and Infrastructure - Clinical Core leaders and key personnel |
| Geoffrey Tremont, PhD | Rhode Island Hospital | ADNI investigators |
| George Bartzokis, MD | University of California, Los Angeles | Past Investigator; ADNI investigators |
| Georgia Stobbs-Cucchi, RN, CCRP | Washington University, St. Louis | Past Investigator; ADNI investigators |
| Ging-Yuek Robin Hsiung, MD, MHSc, FRCPC | U.B.C. Clinic for AD & Related Disorders | ADNI investigators |
| Godfrey Coker, MBA, MPH | University of Southern California | ADNI; Leadership and Infrastructure - Clinical Core leaders and key personnel |
| Godfrey D. Pearlson, MD | Hartford Hospital, Olin Neuropsychiatry Research Center | Past Investigator; ADNI investigators |
| Gregory A. Jicha, MD, PhD | University of Kentucky | ADNI investigators |
| Gustavo Jimenez, MBS | University of Southern California | ADNI; Leadership and Infrastructure - Clinical Core leaders and key personnel |
| Haley Bernhardt, BA, R. EEG T | Washington University St. Louis | ADNI; Leadership and Infrastructure - Neuropathology Core Leaders and Key Personnel |
| Hector González | University of California, San Diego | ADNI; Leadership and Infrastructure - ADNI External Advisory Board (ESAB) |
| Helen Vanderswag, RN | University of California – San Diego | ADNI investigators |
| Hillel Grossman, MD | Mount Sinai School of Medicine | ADNI investigators |
| Horacio Capote, MD | Brigham and Women's Hospital | ADNI investigators |
| Howard Chertkow, MD | McGill Univ., Montreal-Jewish General Hospital | ADNI investigators |
| Howard Feldman, MD, FRCPC | U.B.C. Clinic for AD & Related Disorders | Past Investigator; ADNI investigators |
| Howard J. Rosen, MD | University of California, San Francisco | ADNI investigators |
| Hristina Koleva, MD | University of Iowa College of Medicine | ADNI investigators |
| Hyungsub Shim, MD | University of Iowa College of Medicine | ADNI investigators |
| Ian Grant, MD | Northwestern University | ADNI investigators |
| Ian Malone, PhD | University College London | ADNI; Leadership and Infrastructure - MRI Core Leaders and Key Personnel |
| Ihab Hajjar, MD | Duke University Medical Center | ADNI investigators |
| Irina Rachinsky, MD | St. Joseph’s Health Care | ADNI investigators |
| Jacobo Mintzer, MD, MBA | Roper St. Francis Healthcare | ADNI investigators |
| Jaila Coleman, BA | Stanford University | ADNI investigators |
| James Brewer, MD, PhD | University of California – San Diego | ADNI investigators |
| James J. Lah, MD, PhD | Emory University | ADNI investigators |
| Jamika Singleton-Garvin, CCRP | New York University | Past Investigator; ADNI investigators |
| Janet S. Cellar, DNP, PMHCNS-BC | Duke University Medical Center | Past Investigator; ADNI investigators |
| Jared R. Brosch, MD | Indiana University | ADNI investigators |
| Jared Tinklenberg, MD | Stanford University | Past Investigator; ADNI investigators |
| Jason H. Karlawish, MD | University of Pennsylvania | ADNI investigators |
| Javed I. Khan, MD | Howard University | ADNI investigators |
| Javier Villanueva-Meyer, MD | Baylor College of Medicine | ADNI investigators |
| Jeff D. Williamson, MD, MHS | Wake Forest University Health Sciences | ADNI investigators |
| Jeff Gunter, PhD | Mayo Clinic | ADNI; Leadership and Infrastructure - MRI Core Leaders and Key Personnel |
| Jeffrey A. Kaye, MD, A | Oregon Health & Science University | Past Investigator; ADNI investigators |
| Jeffrey M. Burns, MD | University of Kansas, Medical Center | ADNI investigators |
| Jeffrey R. Petrella, MD | Duke University Medical Center | ADNI investigators |
| Jennifer Salazar, MBS | University of Southern California | ADNI; Leadership and Infrastructure - Clinical Core leaders and key personnel |
| Jeremy Pizzola, BA | University of Southern California | ADNI; Leadership and Infrastructure - Clinical Core leaders and key personnel |
| Jerome Yesavage, MD | Stanford University | ADNI investigators |
| Jesse Mez, MD, MS | Boston University | ADNI investigators |
| Jessica D. White, BA | Stanford University | Past Investigator; ADNI investigators |
| Jessica Poe, BS | Georgetown University Medical Center | ADNI investigators |
| Jiong Shi, MD, PhD | Barrow Neurological Institute | ADNI investigators |
| Jo Cleveland, MD | Wake Forest University Health Sciences | ADNI investigators |
| Joanne L. Lord, LPN, BA, CCRC | University of Michigan | Past Investigator; ADNI investigators |
| John Brockington, MD | University of California – San Diego | Past Investigator; ADNI investigators |
| John C. Morris, MD | Washington University St. Louis | ADNI; Leadership and Infrastructure - Executive Committee, ADNI External Advisory Board (ESAB), Investigator by site |
| John K. Hsiao, MD | NIH | ADNI; Leadership and Infrastructure - ADNI External Advisory Board (ESAB), NIA |
| John Neuhaus, PhD | University of California, San Francisco | ADNI; Leadership and Infrastructure - Administrative Core Northern California Institute for Research & Education (NCIRE / The Vererans Health Research Institute) |
| John Olichney, MD | University of California, Davis – Sacramento | ADNI investigators |
| John Q. Trojanowki, MD, PhD | Perelman School of Medicine, University of Pennsylvania | ADNI; Leadership and Infrastructure - Executive Committee, Biomarkers Core Leaders and Key Personnel (Co-PI) |
| John Rogers, MD | St. Joseph’s Health Care | Past Investigator; ADNI investigators |
| Jonathan D. Drake, MD | Dent Neurologic Institute | ADNI investigators |
| Jonathan Greenberg, BA | Mount Sinai School of Medicine | ADNI investigators |
| Jonathan Jackson, PhD | Massachusetts General Hospital | ADNI; Leadership and Infrastructure - ADNI External Advisory Board (ESAB) |
| Jordan Robson | Northwestern University | ADNI investigators |
| Joseph Masdeu, PhD | Houston Methodist Neurological Institute | ADNI investigators |
| Joseph Quinn, MD | Oregon Health & Science University | Past Investigator; ADNI investigators |
| Joseph S. Kass, MD, LD, FAAN | Baylor College of Medicine | ADNI investigators |
| Joy L. Taylor, PhD | Stanford University | ADNI investigators |
| Judith L. Heidebrink, MD, MS | University of Michigan | ADNI investigators |
| Julia Rao, PhD | New York University | ADNI investigators |
| Karen Anderson, RN | Hartford Hospital, Olin Neuropsychiatry Research Center | Past Investigator; ADNI investigators |
| Karen Crawford | University of Southern California | ADNI; Leadership and Infrastructure - Informatics Core Leaders and Key Personnel, MRI Core Leaders and Key Personnel |
| Karen Dagerman, MS | Oregon Health & Science University | ADNI investigators |
| Karen Ekstam Smith, RN | University of Iowa College of Medicine | ADNI investigators |
| Karen M. Castro, BA | New York University | ADNI investigators |
| Kathleen Johnson, NP | Georgetown University Medical Center | ADNI investigators |
| Kathleen Tingus, PhD | University of California, Los Angeles | Past Investigator; ADNI investigators |
| Kaycee M. Sink, MD, MAS | Wake Forest University Health Sciences | Past Investigator; ADNI investigators |
| Keith A. Johnson, MD | Brigham and Women's Hospital | ADNI investigators |
| Kejal Kantarci, MD | Mayo Clinic | ADNI; Leadership and Infrastructure - MRI Core Leaders and Key Personnel |
| Kelley Faber, MS, CCRC | NCRAD/Indiana University School of Medicine | ADNI; Leadership and Infrastructure - Genetics Core Leaders and Key Personnel |
| Kelly A. Clark | Banner Sun Health Research Institute | ADNI investigators |
| Kelly MCCann, BA | Georgetown University Medical Center | ADNI investigators |
| Kelly Nudelman, PhD | NCRAD/Indiana University School of Medicine | ADNI; Leadership and Infrastructure - Genetics Core Leaders and Key Personnel |
| Kewei Chen, PhD | Banner Alzheimer’s Institute | ADNI; Leadership and Infrastructure - PET Core Leaders and Key Personnel |
| Ki Won Nam, MD | University of Iowa College of Medicine | Past Investigator; ADNI investigators |
| Kim Martin, RN | University of Rochester Medical Center | ADNI investigators |
| Kim Poki-Walker, BA | Mayo Clinic, Jacksonville | ADNI investigators |
| Kris Johnson, RN | Mayo Clinic, Rochester | ADNI investigators |
| Kristi Wilmes, MS, CCRP | NCRAD/Indiana University School of Medicine | ADNI; Leadership and Infrastructure - Genetics Core Leaders and Key Personnel |
| Kristin Fargher, MD | University of South Florida: USF Health Byrd Alzheimer’s Institute | Past Investigator; ADNI investigators |
| Kristine Lipowski, MA | Northwestern University | Past Investigator; ADNI investigators |
| Kwangsik Nho, PhD | Indiana University School of Medicine | ADNI; Leadership and Infrastructure - Genetics Core Leaders and Key Personnel |
| Kyle Womack, MD | University of Texas Southwestern Medical School | ADNI investigators |
| Laura A. Flashman, PhD | Dartmouth-Hitchcock Medical Center | Past Investigator; ADNI investigators |
| Laura L. Boles Ponto, PhD | University of Iowa College of Medicine | ADNI investigators |
| Laurel Beckett, PhD | University of California, Davis | ADNI; Leadership and Infrastructure - Executive Committee, Biostatistics Core Leaders and Key Personnel |
| Laurie Ryan, PhD | NIA | ADNI; Leadership and Infrastructure - ADNI External Advisory Board (ESAB) |
| Lawrence S. Honig, MD, PhD | Columbia University Medical Center | ADNI investigators |
| Lee, PhD | Parkwood Institute | ADNI investigators |
| Leon Thal, MD | University of California, San Diego | Past investigator - ADNI; Leadership and Infrastructure - Initial Concept Planning & Development |
| Leonie Farrington, RN | Johns Hopkins University | ADNI investigators |
| Leslie M. Shaw, PhD | Perelman School of Medicine, University of Pennsylvania | ADNI; Leadership and Infrastructure - Executive Committee, Biomarkers Core Leaders and Key Personnel (Co-PI) |
| Li Shen, PhD | UPenn School of Medicine | ADNI; Leadership and Infrastructure - Genetics Core Leaders and Key Personnel |
| Liana G. Apostolova, MD | Indiana University School of Medicine | ADNI; Leadership and Infrastructure - Genetics Core Leaders and Key Personnel, Past investigator University of California, Los Angeles |
| Liberty Teodoro, RN | Oregon Health & Science University | ADNI investigators |
| Lindsey Hergesheimer, BS | University of Southern California | ADNI; Leadership and Infrastructure - Clinical Core leaders and key personnel |
| Lisa Barnes, PhD | Rush University | ADNI; Leadership and Infrastructure - ADNI External Advisory Board (ESAB) |
| Lisa C. Silbert, MD | Oregon Health & Science University | ADNI investigators |
| Lisa Ravdin, PhD | Dartmouth-Hitchcock Medical Center | Past Investigator; ADNI investigators |
| Lisa Taylor-Reinwald, BA, HTL | Washington University St. Louis | ADNI; Leadership and Infrastructure - Neuropathology Core Leaders and Key Personnel |
| Lisa Zbizek-Nulph, MS, CCRP | University of Michigan | ADNI investigators |
| Lon S. Schneider, MD | Oregon Health & Science University | ADNI investigators |
| Lori A. Daiello, Pharm.D, ScM | Dent Neurologic Institute | ADNI investigators |
| M. Saleem Ismail, MD | University of Rochester Medical Center | Past Investigator; ADNI investigators |
| M.-Marsel Mesulam, MD | Northwestern University | Past Investigator; ADNI investigators |
| Magdalena Korecka, PhD | Perelman School of Medicine, University of Pennsylvania | ADNI; Leadership and Infrastructure - Biomarkers Core Leaders and Key Personnel |
| Marc Seltzer, MD | Dartmouth-Hitchcock Medical Center | Past Investigator; ADNI investigators |
| Maria Carrillo, PhD | Alzheimer’s Association | ADNI; Leadership and Infrastructure - ADNI External Advisory Board (ESAB) |
| Maria Carroll, RN, MSN, GCNS-BC | Washington University, St. Louis | ADNI investigators |
| Maria Kataki, MD, PhD | Ohio State University | ADNI investigators |
| Maria T. Greig-Custo, MD | Wien Center | ADNI investigators |
| Marie Bernard, MD | NIA | ADNI; Leadership and Infrastructure - ADNI External Advisory Board (ESAB) |
| Marilyn Albert, PhD | Johns Hopkins University | ADNI investigators |
| Marissa Natelson Love, MD | University of Alabama - Birmingham | ADNI investigators |
| Mark A. Mintun, MD | Washington University, St. Louis | Past Investigator; ADNI investigators |
| Mark Choe, BS | NCIRE / The Vererans Health Research Institute | ADNI; Leadership and Infrastructure - MRI Core Leaders and Key Personnel |
| Martha G. MacAvoy, PhD | Yale University School of Medicine | ADNI investigators |
| Martin A. Goldstein, MD | Mount Sinai School of Medicine | ADNI investigators |
| Martin R. Farlow, MD | Indiana University | ADNI investigators |
| Martin Sadowski, MD, PhD | New York University | ADNI investigators |
| Marwan Sabbagh, MD | Cleveland Clinic Lou Ruvo Center for Brain Health | ADNI investigators , past investigator of Banner Sun Health Research Institute and Barrow Neurological Institute |
| Mary L. Creech, RN, MSW | Washington University, St. Louis | Past Investigator; ADNI investigators |
| Mary L. Hynes, RN, MPH | Dartmouth-Hitchcock Medical Center | Past Investigator; ADNI investigators |
| Mary Quiceno, MD | University of Texas Southwestern Medical School | Past Investigator; ADNI investigators |
| MaryAnn Oakley, MA | University of Pittsburgh | ADNI investigators |
| Matthew Senjem, MS | Mayo Clinic | ADNI; Leadership and Infrastructure - MRI Core Leaders and Key Personnel |
| Mauricio Becerra, MD | Oregon Health & Science University | ADNI investigators |
| Megan Witbracht, PhD | University of California Irvine IMIND | ADNI investigators |
| Melanie Keltz, RN | University of Rochester Medical Center | ADNI investigators |
| Melanie Shulman, MD | New York University | ADNI investigators |
| Melissa Lamar, PhD | Rush University Medical Center | ADNI investigators |
| Mia Yang, MD | Wake Forest University Health Sciences | Past Investigator; ADNI investigators |
| Michael Borrie, MB ChB T-Y | Parkwood Institute | ADNI investigators |
| Michael Donohue, PhD | University of Southern California | ADNI; Leadership and Infrastructure - Clinical Core leaders and key personnel, Biostatistics Core Leaders and Key Personnel |
| Michael Lin, MD | Dartmouth-Hitchcock Medical Center | Past Investigator; ADNI investigators |
| Michael Rafii, MD, PhD | University of Southern California | ADNI; Leadership and Infrastructure - Clinical Core leaders and key personnel |
| Michael W. Weiner, MD | University of California, San Francisco | ADNI I, GO, II, III; Leadership and Infrastructure - Principal Investigator, Executive Committee, Administrative Core Northern California Institute for Research & Education (NCIRE / The Vererans Health Research Institute), Initial Concept Planning & Development |
| Michal Figurski, PhD | Perelman School of Medicine, University of Pennsylvania | ADNI; Leadership and Infrastructure - Biomarkers Core Leaders and Key Personnel |
| Michele Assaly, MA | U.B.C. Clinic for AD & Related Disorders | Past Investigator; ADNI investigators |
| Michelle Rainka, PharmD, CCRP | Brigham and Women's Hospital | ADNI investigators |
| Mimi Dang, MD | Baylor College of Medicine | Past Investigator; ADNI investigators |
| Miriam Ashford, PhD | NCIRE / The Vererans Health Research Institute | ADNI; Leadership and Infrastructure - Administrative Core Northern California Institute for Research & Education (NCIRE / The Vererans Health Research Institute) |
| Mohammed O. Sheikh, MD | New York University | Past Investigator; ADNI investigators |
| Munir Chowdhury, MBBS, MS | Baylor College of Medicine | Past Investigator; ADNI investigators |
| Nancy Johnson, PhD | Northwestern University | Past Investigator; ADNI investigators |
| Nancy Kowalski, MS, RNC | University of Rochester Medical Center | ADNI investigators |
| Nathaniel Pacini, MA | Baylor College of Medicine | ADNI investigators |
| Neda Jahanshad, PhD | University of Southern California School of Medicine | ADNI; Leadership and Infrastructure - MRI Core Leaders and Key Personnel |
| Neil Kowall, MD | Boston University | Past Investigator; ADNI investigators |
| Neill R Graff-Radford, MBBCH, FRCP (London) | Mayo Clinic, Jacksonville | ADNI investigators |
| Nick C. Fox, MD | University College London | ADNI; Leadership and Infrastructure - MRI Core Leaders and Key Personnel |
| Nigel J. Cairns, PhD, FRCPath | Washington University St. Louis | Past Investigator; ADNI; Leadership and Infrastructure - Neuropathology Core Leaders and Key Personnel |
| Nina Silverberg, PhD | NIA | ADNI; Leadership and Infrastructure - ADNI External Advisory Board (ESAB) |
| Norman Relkin, MD, PhD | Cornell University | Past Investigator; ADNI investigators |
| Nunzio Pomara, MD | Nathan Kline Institute | ADNI investigators |
| Olga James, MD | Duke University Medical Center | ADNI investigators |
| Olusegun Adegoke, MSc | University of Southern California | ADNI; Leadership and Infrastructure - Clinical Core leaders and key personnel |
| Oscar L. Lopez, MD | University of Pittsburgh | ADNI investigators |
| Owen Carmichael, PhD | University of California, Davis – Sacramento | Past Investigator; ADNI investigators |
| Oyonumo E. Ntekim, MD, PhD | Howard University | ADNI investigators |
| P. Murali Doraiswamy, MBBS, FRCP | Duke University Medical Center | ADNI investigators |
| Parianne Fatica, BA, CCRC | Case Western Reserve University | ADNI investigators |
| Patricia Samuels | Rush University Medical Center | ADNI investigators |
| Paul A. Yushkevich, PhD | University of Pennsylvania | ADNI; Leadership and Infrastructure - MRI Core Leaders and Key Personnel |
| Paul Aisen, MD | University of Southern California | ADNI; Leadership and Infrastructure - ATRI PI and Director of Coordinating Center Clinical Core, Executive Commitee, Clinical Core leaders and key personnel |
| Paul Malloy, PhD | Butler Hospital Memory and Aging Program | ADNI investigators |
| Paul Newhouse, PhD | Vanderbilt University Medical Center | ADNI investigators |
| Paul Thompson, PhD | University of Southern California School of Medicine | ADNI; Leadership and Infrastructure - MRI Core Leaders and Key Personnel |
| Paula Ogrocki, PhD | Case Western Reserve University | ADNI investigators |
| Pauline Maillard, PhD | University of California, Davis – Sacramento | ADNI investigators |
| Payam Mahboubi, MPH | University of Southern California | ADNI; Leadership and Infrastructure - Clinical Core leaders and key personnel |
| Po H. Lu, PsyD | University of California, Los Angeles | Past Investigator; ADNI investigators |
| Pradeep Varma, MD | Yale University School of Medicine | ADNI investigators |
| Prashanthi Vemuri, PhD | Mayo Clinic | ADNI; Leadership and Infrastructure - MRI Core Leaders and Key Personnel |
| Rachel Crissey | Oregon Health & Science University | ADNI investigators |
| Rachel Nosheny, PhD | University of California, San Francisco | ADNI; Leadership and Infrastructure - Administrative Core Northern California Institute for Research & Education (NCIRE / The Vererans Health Research Institute) |
| Rachelle S. Doody, MD, PhD | Baylor College of Medicine | Past Investigator; ADNI investigators |
| Raina Carter, BA | Oregon Health & Science University | Past Investigator; ADNI investigators |
| Raj C. Shah, MD | Rush University Medical Center | ADNI investigators |
| Randall Griffith, PhD, ABPP | University of California – San Diego | Past Investigator; ADNI investigators |
| Ranjan Duara, MD | Wien Center | ADNI investigators |
| Rawan Tarawneh, MD | Ohio State University | ADNI investigators |
| Raymond Scott Turner, MD, PhD | Georgetown University Medical Center | ADNI investigators |
| Raymundo Hernando, MD | Nathan Kline Institute | ADNI investigators |
| Reisa A. Sperling, MD | Brigham and Women's Hospital | ADNI investigators |
| Rema Raman, PhD | University of Southern California | ADNI; Leadership and Infrastructure - Clinical Core leaders and key personnel |
| Richard E. Carson, PhD | Yale University School of Medicine | ADNI investigators |
| Richard J. Perrin, MD, PhD | Washington University St. Louis | ADNI; Leadership and Infrastructure - Executive Committee, ADNI External Advisory Board (ESAB), Neuropathology Core Leaders and Key Personnel |
| Robert A. Koeppe, PhD | University of Michigan | ADNI; Leadership and Infrastructure - PET Core Leaders and Key Personnel |
| Robert B. Santulli, MD | Dartmouth-Hitchcock Medical Center | Past Investigator; ADNI investigators |
| Robert C. Green, MD, MPH | Brigham and Women’s Hospital/Harvard Medical School | ADNI; Leadership and Infrastructure - Executive Committee, Data and Publications Committee |
| Robert Reid, PhD | Mayo Clinic | ADNI; Leadership and Infrastructure - MRI Core Leaders and Key Personnel |
| Robert Stern, PhD | Boston University | ADNI investigators |
| Ronald Killiany, PhD | Boston University | ADNI investigators |
| Ronald Petersen, MD, PhD | Mayo Clinic, Rochester | ADNI; Leadership and Infrastructure - ATRI PI and Director of Coordinating Center Clinical Core; Co-PI of clinical core, Executive Commitee, Clinical Core leaders and key personnel, Investigator of Mayo Clinic |
| Rosemarie Rodriguez, PhD | Wien Center | ADNI investigators |
| Rottislav Brichko, BS | Johns Hopkins University | ADNI investigators |
| Russell H. Swerdlow, MD | University of Kansas, Medical Center | ADNI investigators |
| Saba Wolday, MSc | Howard University | ADNI investigators |
| Salvador Borges-Neto, MD | University of Alabama - Birmingham | Past Investigator; ADNI investigators |
| Sandhitsu Das, PhD | University of Pennsylvania | ADNI; Leadership and Infrastructure - MRI Core Leaders and Key Personnel |
| Sandra Black, MD, FRCPC | Sunnybrook Health Sciences, Ontario | ADNI investigators |
| Sandra Weintraub, PhD | Northwestern University | Past Investigator; ADNI investigators |
| Sanjay Asthana, MD | University of Wisconsin | ADNI investigators |
| Sanjeev Vaishnavi, MD | University of Pennsylvania | ADNI investigators |
| Sara Dolen, BS | Oregon Health & Science University | Past Investigator; ADNI investigators |
| Sara S. Mason, RN | Mayo Clinic, Rochester | ADNI investigators |
| Sarah Kremen, MD | University of California, Los Angeles | ADNI investigators |
| Sarah Walter, MSc | University of Southern California | ADNI; Leadership and Infrastructure - Clinical Core leaders and key personnel |
| Scott Herring, RN, CCRC | Indiana University | ADNI investigators |
| Scott Neu, PhD | University of Southern California | ADNI; Leadership and Infrastructure - Informatics Core Leaders and Key Personnel |
| Scott Rudow, BS | Johns Hopkins University | ADNI investigators |
| Shannon Chen, BA | New York University | ADNI investigators |
| Shannon L. Risacher, PhD | Indiana University School of Medicine | ADNI; Leadership and Infrastructure - Genetics Core Leaders and Key Personnel |
| Sheeba Nadarajah, PhD | Howard University | ADNI investigators |
| Shelley Moore, BA | University of Southern California | ADNI; Leadership and Infrastructure - Clinical Core leaders and key personnel |
| Smita Kittur, MD | Neurological Care of CNY | Past Investigator; ADNI investigators |
| Sonia Pawluczyk, MD | Oregon Health & Science University | ADNI investigators |
| Sophia I. Thomopoulos, BS | University of Southern California School of Medicine | ADNI; Leadership and Infrastructure - MRI Core Leaders and Key Personnel |
| Stacy Schneider, APRN, BC, GNP | Washington University, St. Louis | Past Investigator; ADNI investigators |
| Stephanie Kielb, BS | Johns Hopkins University | Past Investigator; ADNI investigators |
| Stephanie Rossi Chen, BA. | NCIRE / The Vererans Health Research Institute | ADNI; Leadership and Infrastructure - MRI Core Leaders and Key Personnel |
| Stephanie Smith, BS | University of Southern California | ADNI; Leadership and Infrastructure - Clinical Core leaders and key personnel |
| Stephen Correia, PhD | Butler Hospital Memory and Aging Program | ADNI investigators |
| Stephen Pasternak, MD | St. Joseph’s Health Care | ADNI investigators |
| Stephen Salloway, MD, MS | Butler Hospital Memory and Aging Program | ADNI investigators |
| Sterling Johnson, PhD | University of Wisconsin | ADNI investigators |
| Steven Chao, MD, PhD | Stanford University | ADNI investigators |
| Steven E. Arnold, MD | University of Pennsylvania | Past Investigator; ADNI investigators |
| Steven Potkin, PhD | Long Beach VA Neuropsychiatric Research Program | ADNI investigators |
| Susan K. Schultz, MD | University of Iowa College of Medicine | Past Investigator; ADNI investigators |
| Susan Landau, PhD | University of California, Berkeley | ADNI; Leadership and Infrastructure - PET Core Leaders and Key Personnel |
| Susan P. Good, APRN | Yale University School of Medicine | ADNI investigators |
| Susan Rountree, MD | Baylor College of Medicine | Past Investigator; ADNI investigators |
| Susan Vaitekunis, MD | McGill Univ., Montreal-Jewish General Hospital | ADNI investigators |
| Suzanne Craft, PhD | Wake Forest University Health Sciences | ADNI investigators |
| Talia M. Nir, PhD | University of Southern California School of Medicine | ADNI; Leadership and Infrastructure - MRI Core Leaders and Key Personnel |
| Tatiana M. Foroud, PhD | NCRAD/Indiana University School of Medicine | ADNI; Leadership and Infrastructure - Genetics Core Leaders and Key Personnel |
| Taylor Clanton, MPH | University of Southern California | ADNI; Leadership and Infrastructure - Clinical Core leaders and key personnel |
| Terence Z. Wong, MD | University of Alabama - Birmingham | ADNI investigators |
| Thomas O. Obisesan, MD, MPH | Howard University | ADNI investigators |
| Thomas Wisniewski, MD | New York University | ADNI investigators |
| Tom Montine, MD, PhD | University of Washington (Chair) | ADNI; Leadership and Infrastructure - Resource Allocation Review Committee |
| Trung Nguyen, MD | University of Texas Southwestern Medical School | ADNI investigators |
| Valory Pavlik, PhD | Baylor College of Medicine | ADNI investigators |
| Vernice Bates, MD | Dent Neurologic Institute | ADNI investigators |
| Vesna Sossi, PhD | U.B.C. Clinic for AD & Related Disorders | ADNI investigators |
| Victoria Shibley, MS | Baylor College of Medicine | Past Investigator; ADNI investigators |
| William Jagust, MD | University of California, Berkeley | ADNI; Leadership and Infrastructure - Executive Committee, PET Core Leaders and Key Personnel |
| William M. Brooks, PhD | University of Kansas, Medical Center | ADNI investigators |
| William Potter, MD | National Institute of Mental Health | ADNI; Leadership and Infrastructure - ADNI External Advisory Board (ESAB) |
| Yaakov Stern, PhD | Columbia University Medical Center | ADNI investigators |
| Yuliana Cabrera, BS | University of Southern California | ADNI; Leadership and Infrastructure - Clinical Core leaders and key personnel |
| Zaven Khachaturian, PhD | Prevent Alzheimer’s Disease 2020 | ADNI; Leadership and Infrastructure - ADNI External Advisory Board (ESAB Chair), Initial Concept Planning & Development |

## A4 study team

Reisa Sperling, MD (Harvard Medical School, Brigham and Women’s Hospital, Massachusetts General Hospital, Leadership Team); Paul Aisen, MD (University of Southern California, Alzheimer’s Therapeutic Research Institute, Leadership Team); Roy Yaari, MD (Eli Lilly and Company, Leadership Team); Cheryl A. Brown, RPh, PMP (Eli Lilly and Company, Leadership Team); John R. Sims, MD (Eli Lilly and Company, Leadership Team); Keith Johnson, MD (PET Imaging, Team Leader); Clifford Jack Jr., MD (MRI – Mayo Clinic, Team Leader); James B. Brewer, MD, PhD (ADCS Imaging, Team Leader); Jason Karlawish, MD (Ethics Committee, Team Leader); Joshua D. Grill, PhD (Ethics Committee, Team Leader) Marybeth Howlett, MEM (AVID, Team Leader); Paul Maruff, PhD (Cogstate, Team Leader); Kenneth Marek, MD (Invicro, Team Leader); John Seibyl, MD (Invicro, Team Leader); Mark Mintun, MD (Eli Lilly and Company, Team Leader); Karen Holdridge, MPH (Eli Lilly and Company, Team Leader); Isabella Velona, MS (Eli Lilly and Company, Team Leader); Alison Belsha, BS (University of Southern California, Alzheimer’s Therapeutic Research Institute, Team Leader); Jeremy Pizzola (University of Southern California, Alzheimer’s Therapeutic Research Institute, Team Leader); Robert Rissman, PhD (University of Southern California, Alzheimer’s Therapeutic Research Institute, Team Leader); Cecily Jenkins, PhD (University of Southern California, Alzheimer’s Therapeutic Research Institute, Team Leader); Michael Donohue, PhD (University of Southern California, Alzheimer’s Therapeutic Research Institute, Team Leader); Rema Raman, PhD (University of Southern California, Alzheimer’s Therapeutic Research Institute, Team Leader); Gustavo Jimenez-Maggiora, MBA (University of Southern California, Alzheimer’s Therapeutic Research Institute, Team Leader); Mike Rafii, MD, PhD (University of Southern California, Alzheimer’s Therapeutic Research Institute, Team Leader); Aaron Schultz, PhD (Brigham and Women’s Hospital, Massachusetts General Hospital, Harvard Medical School); Dorene Rentz, PsyD (Brigham and Women’s Hospital, Massachusetts General Hospital, Harvard Medical School); Kate Papp, PhD (Brigham and Women’s Hospital, Massachusetts General Hospital, Harvard Medical School); Beth Mormino, PhD (Brigham and Women’s Hospital, Massachusetts General Hospital, Harvard Medical School); Rebecca Amariglio, PhD (Brigham and Women’s Hospital, Massachusetts General Hospital, Harvard Medical School); Gad Marshall, MD (Brigham and Women’s Hospital, Massachusetts General Hospital, Harvard Medical School); Dylan Kirn (Brigham and Women’s Hospital, Massachusetts General Hospital, Harvard Medical School); Michael Properzi (Brigham and Women’s Hospital, Massachusetts General Hospital, Harvard Medical School); J. Alex Becker, PhD (Brigham and Women’s Hospital, Massachusetts General Hospital, Harvard Medical School); Rachel Buckley, PhD (Brigham and Women’s Hospital, Massachusetts General Hospital, Harvard Medical School); Jorge Gutierrez (University of Southern California, Alzheimer’s Therapeutic Research Institute, Clinical Operations); Harriett Davey (University of Southern California, Alzheimer’s Therapeutic Research Institute, Clinical Operations); Melissa Ruiz (University of Southern California, Alzheimer’s Therapeutic Research Institute, Clinical Operations); Vedeline Torreon, BS (University of Southern California, Alzheimer’s Therapeutic Research Institute, Medical Safety/Clinical Monitoring); Marianne Manire, BS (University of Southern California, Alzheimer’s Therapeutic Research Institute, Medical Safety/Clinical Monitoring); Renarda Jones, MS (University of Southern California, Alzheimer’s Therapeutic Research Institute, Medical Safety/Clinical Monitoring); Isabel Francis (University of Southern California, Alzheimer’s Therapeutic Research Institute, Clinical Operations); Maria Arampatzidou, PhD (University of Southern California, Alzheimer’s Therapeutic Research Institute, Clinical Operations); Paula Cohen, BA (University of Southern California, Alzheimer’s Therapeutic Research Institute, Clinical Operations); Jennifer Salazar, MBS (University of Southern California, Alzheimer’s Therapeutic Research Institute, Clinical Operations); Gabriela Muranevici, MD, PhD (University of Southern California, Alzheimer’s Therapeutic Research Institute, Former Medical Monitor, Medical Safety/Clinical Monitoring); Tiffany Chow, MD (University of Southern California, Alzheimer’s Therapeutic Research Institute, Former Director, Clinical Monitoring, Medical Safety/Clinical Monitoring); Steve Bruno III, BA (University of Southern California, Alzheimer’s Therapeutic Research Institute, Former Lead Clinical Monitor, Medical Safety/Clinical Monitoring); Gina Garcia-Camilo, MD (University of Southern California, Alzheimer’s Therapeutic Research Institute, Former Clinical Monitor, Medical Safety/Clinical Monitoring); Adriana Bohorquez, MD (University of Southern California, Alzheimer’s Therapeutic Research Institute, Former Medical Monitor, Medical Safety/Clinical Monitoring); Alyssa Carroll (Schmitt), MS (University of Southern California, Alzheimer’s Therapeutic Research Institute, Former Clinical Monitor Manager, Medical Safety/Clinical Monitoring); Sarah Danowski, MA (University of Southern California, Alzheimer’s Therapeutic Research Institute, Former Clinical Monitor Manager, Medical Safety/Clinical Monitoring); Deborah Tobias (University of Southern California, Alzheimer’s Therapeutic Research Institute, Former Administration Director); Lindsey Earp, BA (University of Southern California, Alzheimer’s Therapeutic Research Institute, Administration); Dan Abinsay (University of Southern California, Alzheimer’s Therapeutic Research Institute, Administration); Sarah Walter, MSc (University of Southern California, Alzheimer’s Therapeutic Research Institute, Administration); Ryoko Ihara, MD, PhD (University of Southern California, Alzheimer’s Therapeutic Research Institute, Neuropsychology); Cecily Jenkins, PhD (University of Southern California, Alzheimer’s Therapeutic Research Institute, Neuropsychology); Xavier Salazar, PhD (University of Southern California, Alzheimer’s Therapeutic Research Institute, Neuropsychology); Stefanie Juliano, MA (University of Southern California, Alzheimer’s Therapeutic Research Institute, Neuropsychology); Sarah Espinoza, BA (University of Southern California, Alzheimer’s Therapeutic Research Institute, Neuropsychology); Shelley Moore, BA (University of Southern California, Alzheimer’s Therapeutic Research Institute, Recruitment & Retention, Administration); Taylor Clanton, MPH, CHES (University of Southern California, Alzheimer’s Therapeutic Research Institute, Recruitment & Retention); Agnes Lewandowski, M.Res (University of Southern California, Alzheimer’s Therapeutic Research Institute, Recruitment & Retention); Phuoc Hong, BA (University of Southern California, Alzheimer’s Therapeutic Research Institute, IT); Hongmei Qiu, MS (University of Southern California, Alzheimer’s Therapeutic Research Institute, Informatics); Jia-shing So, BS (University of Southern California, Alzheimer’s Therapeutic Research Institute, Informatics); Stefania Bruschi, MS, MBA (University of Southern California, Alzheimer’s Therapeutic Research Institute, Informatics); Kimberlee Eudy, JD (University of Southern California, Alzheimer’s Therapeutic Research Institute, Contracts); Quin Revel, JD (University of Southern California, Alzheimer’s Therapeutic Research Institute, Contracts); Michael Selsnik, BS (University of Southern California, Alzheimer’s Therapeutic Research Institute, Contracts); Olusegun Adegoke, MSc (University of Southern California, Alzheimer’s Therapeutic Research Institute, Data Management); Veasna Tan, MA (University of Southern California, Alzheimer’s Therapeutic Research Institute, Data Management); Olga Baryshnikava, MS (University of Southern California, Alzheimer’s Therapeutic Research Institute, Data Management); Iris Sim Dacio, BA (University of Southern California, Alzheimer’s Therapeutic Research Institute, Data Management); Sandhya Niranjan Jaiswal, B Pharm (University of Southern California, Alzheimer’s Therapeutic Research Institute, Data Management); Elizabeth Shaffer, BS (University of Southern California, Alzheimer’s Therapeutic Research Institute, Regulatory Affairs); Michelle Pablo, BS (University of Southern California, Alzheimer’s Therapeutic Research Institute, Regulatory Affairs); Karin Ernstrom, MS (University of Southern California, Alzheimer’s Therapeutic Research Institute, Biostatistics); Gopalan Sethuraman, PhD (University of Southern California, Alzheimer’s Therapeutic Research Institute, Biostatistics); Jiyoon Choi, MS (University of Southern California, Alzheimer’s Therapeutic Research Institute, Biostatistics); Oliver Langford, MS (University of Southern California, Alzheimer’s Therapeutic Research Institute, Biostatistics); Shunran Wang, MS (University of Southern California, Alzheimer’s Therapeutic Research Institute, Biostatistics); Andy Liu, MS (University of Southern California, Alzheimer’s Therapeutic Research Institute, Biostatistics); Barbara Bartocci, MPH (University of Southern California, Alzheimer’s Therapeutic Research Institute, Quality Assurance); Melissa Korba (University of Southern California, Alzheimer’s Therapeutic Research Institute, Quality Assurance); Sara Abdel-Latif, MBA (University of Southern California, Alzheimer’s Therapeutic Research Institute, Biomarker); Andrea Abram, MBA (Eli Lilly and Company); Bhavna Madduri, MPH (Eli Lilly and Company); Dillon Hilderbrand (Eli Lilly and Company); John Brad Holmes, MBA (Eli Lilly and Company); Julie Chandler, PhD (Eli Lilly and Company); Keith Parsons, BS, MBA (Eli Lilly and Company); Lisa Ferguson-Sells, BSc (Eli Lilly and Company); Marybeth Devine (Eli Lilly and Company); Michael Case, MS (Eli Lilly and Company); Michele Mancini, MD (Eli Lilly and Company); Michael Pontecorvo, PhD (Eli Lilly and Company); Peter Fairfield, MBA (Eli Lilly and Company); Phyllis Ferrell, MBA (Eli Lilly and Company); Satina Hall (Eli Lilly and Company); Scott Kaiser, MBA (Eli Lilly and Company); Sergey Shcherbinin, PhD (Eli Lilly and Company); Stacy Huckins (Eli Lilly and Company); Susan Warner, Pharm.D (Eli Lilly and Company); Jude Burger, MS (Eli Lilly and Company); Bret J. Borowski, RT (R) (MRI – Mayo Clinic); Petrice M. Cogswell, MD (MRI – Mayo Clinic); Cory A. Johnson (MRI – Mayo Clinic); Kejal Kantarci, MD (MRI – Mayo Clinic); Leonard C. Matoush, Jr. (MRI – Mayo Clinic); William C. Turke (MRI – Mayo Clinic); Ashritha L. Reddy (MRI – Mayo Clinic); Denise A. Reyes (MRI – Mayo Clinic); Kaely B. Thostenson, RT (R) (MRI – Mayo Clinic); Samantha M. Zuk (MRI – Mayo Clinic); Jeffrey M. Burns, MD, MS (A4 Study Ethics Committee, University of Kansas Alzheimer’s Disease Center, Member); David Sultzer, MD (A4 Study Ethics Committee, University of California, Irvine, Member); Howard Feldman, MD (Alzheimer’s Disease Cooperative Study [ADCS] – University of California, San Diego, Director (Lead)); Genny Matthews (ADCS – University of California, San Diego, Recruitment); Stephanie Parks (ADCS –University of California, San Diego, Clinical Operations); Jen Mason, MPH (ADCS – University of California, San Diego, Data Management); Jason Young, PhD (ADCS – University of California, San Diego, Informatics); Ashlee Heldreth, BA (ADCS – University of California, San Diego, Monitor); Janet Kastelan, BA (ADCS – University of California, San Diego, Monitor); Rebecca Ryan-Jones, PhD (ADCS – University of California, San Diego, Monitor); Lindsay Cotton (ADCS – University of California, San Diego, Monitor); Maria Bulger Lennox, RN (ADCS – University of California, San Diego, Monitor); Ronelyn Chavez, BA (ADCS – University of California, San Diego, Monitor); Tilman Oltersdorf, MD (ADCS – University of California, San Diego, MedSafety); Curtis Taylor, PhD (ADCS – University of California, San Diego, MedSafety); Barbara LaPlante, MA (ADCS – University of California, San Diego, Transition Team); Meghan Stirn, MBA (ADCS – University of California, San Diego, Transition Team); Joanne Brechlin, MBA, MPH (ADCS – University of California, San Diego, Transition Team); Gina Varner, MPH (ADCS – University of California, San Diego, Transition Team); Carol Evans, BA (ADCS – University of California, San Diego, U19 Grant Administration); Karim Hussein, JD (ADCS – University of California, San Diego, U19 Grant Administration); Erika Wilson (ADCS – University of California, San Diego, Finance); Ronald Thomas, PhD (ADCS – University of California, San Diego, Biostatistics); Sheila Jin, PhD (ADCS – University of California, San Diego, Biostatistics); James Barlow (ADCS – University of California, San Diego, Biomarker); Nichol Ferng, BS (ADCS Neuroimaging – University of California, San Diego); Robin Jennings, BS, MS (ADCS Neuroimaging – University of California, San Diego); Leonardino Digma, BA (ADCS Neuroimaging – University of California, San Diego); Heidi Jacobs, PhD (PET Imaging, Massachusetts General Hospital); Jorge Sepulcre, MD (PET Imaging, Massachusetts General Hospital); Justin Sanchez (PET Imaging, Massachusetts General Hospital); Matthew Scott (PET Imaging, Massachusetts General Hospital); Reneé Tschopp, MS, PMP (Invicro); Donna Miles (Invicro); Amy Frederickson, GradDipBiostats (Cogstage, Melbourne); François Windels, PhD (Cogstate, Brisbane); Bodil Hook, BBSc, PostGradDip in Psychology (Cogstage, Melbourne); Lenny Nuciforo, BS (Cogstage, New York, NY); Natalia Contreras, PhD (Cogstage, Melbourne); Patrick McCabe, BA (McCabe Message Partners); Becky Watt Knight, MA (McCabe Message Partners); Rachel Griffith, BA (McCabe Message Partners); Melissa McGue, BS, BA (McCabe Message Partners); Shannon Conti, MS (McCabe Message Partners); Amy Martin Vogt, BA (McCabe Message Partners); Marissa C. Natelson Love, MD (University of Alabama, Birmingham); P. Denise Ledlow, RN (University of Alabama, Birmingham); Amber Watkins, RN (University of Alabama, Birmingham); David S. Geldmacher, MD (University of Alabama, Birmingham); Loren Brown Ashley, RN (University of Alabama, Birmingham); Jacqueline Vaughn, RN (University of Alabama, Birmingham); William J. Burke, MD (Banner Alzheimer’s Institute); Roma Patel, MS, MBA (Banner Alzheimer’s Institute); Daniel Viramontes Apodaca (Banner Alzheimer’s Institute); Sachin Y. Pandya (Banner Alzheimer’s Institute); Anna D. Burke, MD (Banner Alzheimer’s Institute); Edward Zamrini, MD (Banner Sun Health Research Institute); Zoran Obradov, CRC (Banner Sun Health Research Institute); Christine M. Belden, PsyD (Banner Sun Health Research Institute); Carol Cline, MSW, LMSW, CSP (Banner Sun Health Research Institute, Psychometrist); Margaret Rich, CSP (Banner Sun Health Research Institute, Psychometrist); Lisa Roye, MS (Banner Sun Health Research Institute, Psychometrist); Marwan Sabbagh, MD (Banner Sun Health Research Institute, Past Investigator); Jerome Yesavage, MD (Stanford University School of Medicine, VA Aging Clinical Research Center); Steven Z. Chao, MD, PhD (Stanford University School of Medicine, VA Aging Clinical Research Center); Tamara Beale, MA (Stanford University School of Medicine, VA Aging Clinical Research Center); Jaila Coleman, BA (Stanford University School of Medicine, VA Aging Clinical Research Center); Shawn Kile, MD (Sutter Institute for Medical Research); Valentina Mikhalenko, CRC (Sutter Institute for Medical Research); Yvonne Au, LCSW, PACC (Sutter Institute for Medical Research); Mary Vaughn, RN (Sutter Institute for Medical Research); Sampreet Moneski, CRC (Sutter Institute for Medical Research); Tammy Donnell, CCRC (Sutter Institute for Medical Research); Dawn Lenakakis, CRC (Sutter Institute for Medical Research); John Gregory Duffy, MD (Syrentis Clinical Research); Lorrie Bisesi, PhD (Syrentis Clinical Research); Poonam Nina Banerjee, PhD (Syrentis Clinical Research); Maria Gonzalez, BS (Syrentis Clinical Research); Rania Bilwani, BS (Syrentis Clinical Research); John Olichney, MD (University of California, Davis Alzheimer’s Disease Center East Bay); Charles DeCarli, MD (University of California, Davis Alzheimer’s Disease Center East Bay); Hongzheng Zhang, PhD (University of California, Davis Alzheimer’s Disease Center East Bay); Antoinette Lopez, MD (University of California, Davis Alzheimer’s Disease Center East Bay); Mary McPhail-Ciufo, MD (University of California, Davis Alzheimer’s Disease Center East Bay); Adrian Preda, MD (University of California, Irvine [UCI BIC]); Andrea Weideman (University of California, Irvine [UCI BIC]); Steven Potkin, MD (University of California, Irvine [UCI BIC], Past Investigator); Melanie Tallakson, DNP (University of California, Irvine [UCI MIND]); Catherine McAdams-Ortiz, AGNP (University of California, Irvine [UCI MIND]); Beatriz Vides, MSN, RN (University of California, Irvine [UCI MIND]); Gaby T. Thai, MD (University of California, Irvine [UCI MIND]); Steven P. Tam, MD (University of California, Irvine [UCI MIND]); Maryam Beigi, MD (University of California, Los Angeles); Thao Rodriguez, NP (University of California, Los Angeles); Maya Farchi, CRC (University of California, Los Angeles); Roberto Hernandez, CRC (University of California, Los Angeles); Lorena Monserratt, PhD (University of California, Los Angeles); Lauren Garcia, CRC (University of California, Los Angeles); Celine Ossinalde, MA (University of California, Los Angeles); Douglas Galasko, MD (University of California, San Diego); Helen Vanderswag, RNC, BSN (University of California, San Diego); Laura Linares, RN (University of California, San Diego); Chi Kim, BS (University of California, San Diego); Shawnees Peacock, BS (University of California, San Diego); Adam L. Boxer, MD, PhD (University of California, San Francisco, Memory and Aging Center); Lawren Vandevrede, MD, PhD (University of California, San Francisco, Memory and Aging Center); Peter Ljubenkov, MD (University of California, San Francisco, Memory and Aging Center); Julio C. Rojas, MD, PhD (University of California, San Francisco, Memory and Aging Center); Mauricio Becerra (University of Southern California, Alzheimer’s Disease Research Center); Liberty Teodoro, RN (University of Southern California, Alzheimer’s Disease Research Center); Sonia Pawluczyk, MD (University of Southern California, Alzheimer’s Disease Research Center); Karen Dagerman, MS (University of Southern California, Alzheimer’s Disease Research Center); Lon Schneider, MD (University of Southern California, Alzheimer’s Disease Research Center); Christopher H. van Dyck, MD (Yale Alzheimer’s Disease Research Unit); Julia W. McDonald, BA (Yale Alzheimer’s Disease Research Unit); Susan P. Good, APRN (Yale Alzheimer’s Disease Research Unit); Joanna E. Harris, BA (Yale Alzheimer’s Disease Research Unit); Kara Bates, BS (Yale Alzheimer’s Disease Research Unit); Jessica Lam, BS (Yale Alzheimer’s Disease Research Unit); Raymond Scott Turner, MD, PhD (Georgetown University); Melanie Chadwick, RN, MS, NP (Georgetown University); Kathleen Johnson, RN, MSN, NP (Georgetown University); Brigid Reynolds, RN, MSN, NP (Georgetown University); Kelly McCann, BA (Georgetown University); Thomas O. Obisesan, MD, MPH (Howard University); Oyonumo E. Ntekim, MD, PhD (Howard University); Sheeba R. Nadarajah, PhD (Howard University); Sharlene Leong, MSc (Howard University); Saba Wolday, MSc (Howard University); Jillian Turner (Howard University); Mark Brody, MD (Brain Matters Research); Paayal Patel, MD (Brain Matters Research); Cynthia Stimeck, PA-C (Brain Matters Research); Neill R. Graff-Radford, MD (Mayo Clinic – Jacksonville); Christopher Homa, BS (Mayo Clinic – Jacksonville); Amanda Phillips, BS (Mayo Clinic – Jacksonville); Paul Winner, DO (Premiere Research Institute); Alfonso X. Moreno, MD (Premiere Research Institute); Esteban Olivera, MD (Synexus Clinical Research, Orlando); Jennifer West, PA (Synexus Clinical Research, Orlando); Noureen Dhanani, APRN (Synexus Clinical Research, Orlando, LEARN Study PI); Maria Amy Edridge, L-CRC (Synexus Clinical Research, Orlando); Alisa Petit, MS, CSP (Synexus Clinical Research, Orlando); Yarnick Mirjah, BS (Synexus Clinical Research, Orlando); Olivia Reilly (Synexus Clinical Research, Orlando); Angelica Okolie, MD (Synexus Clinical Research, The Villages); Uzma Khan, MD (Synexus Clinical Research, The Villages); Elma Fallejo (Synexus Clinical Research, The Villages); Amanda G. Smith, MD (USF Health Byrd Alzheimer’s Institute); Juris Jarvis, MD (USF Health Byrd Alzheimer’s Institute); Kelly Rodrigo, BA, CCRC (USF Health Byrd Alzheimer’s Institute); Ijeoma Mba, MBBS (USF Health Byrd Alzheimer’s Institute); Anna D. Sladky, CIP, CRA-USF (USF Health Byrd Alzheimer’s Institute); Patricia Lowe, CCRC, CRA-USF (USF Health Byrd Alzheimer’s Institute); Breanna Davis (USF Health Byrd Alzheimer’s Institute); Ranjan Duara, MD (Wien Center for Alzheimer’s Disease and Dementia); Maria T. Greig Custo, MD (Wien Center for Alzheimer’s Disease and Dementia); Rosemarie A. Rodriguez, PhD (Wien Center for Alzheimer’s Disease and Dementia); Julieth Formosa, PharmD (Wien Center for Alzheimer’s Disease and Dementia); Warren Barker, MA (Wien Center for Alzheimer’s Disease and Dementia); Joyce Lee, PharmD (Wien Center for Alzheimer’s Disease and Dementia); Cesar Chirinos (Wien Center for Alzheimer’s Disease and Dementia); James J. Lah, MD, PhD (Emory University); Allan I. Levey, MD, PhD (Emory University); Deborah Westover, BSN, RN (Emory University); Gail Schwartz, BSN, RN (Emory University); Lauren Mariotti, BS (Emory University); Jeffrey Ross, MD (Great Lakes Clinical Trials); Linda Rice, PhD (Great Lakes Clinical Trials); Sandra Weintraub, PhD (Northwestern University); Ian Grant, MD (Northwestern University); Brittanie Muse, MSPH, CCRC (Northwestern University); Shea Gold, MA (Northwestern University); Jelena Pejic, BS (Northwestern University); Loreece Haddad, MS (Northwestern University); Neelum T. Aggarwal, MD (Rush University Medical Center); Ajay Sood, MD, PhD (Rush University Medical Center); Kimberly Blanchard, DNP, APRN, NP-C (Rush University Medical Center); Peter Lambiotis (Rush University Medical Center); Amelia Troutman (Rush University Medical Center); Martin Farlow, MD (Indiana University); Jared Brosch, MD (Indiana University); Nancy McClaskey, RN (Indiana University); Del D. Miller, PharmD, MD (University of Iowa); Hristina K. Koleva, MD (University of Iowa); Karen Ekstam Smith, RN (University of Iowa); Laura Temple, MS (University of Iowa); Susan Schultz, MD (University of Iowa, Past Investigator); Anne Arthur, APRN (University of Kansas Medical Center Alzheimer’s Disease Center); Rachel Starr, BS, CCRP (University of Kansas Medical Center Alzheimer’s Disease Center); Nicole Mathis, MS (University of Kansas Medical Center Alzheimer’s Disease Center); Phyllis Switzer (University of Kansas Medical Center Alzheimer’s Disease Center); Gregory A. Jicha, MD, PhD (University of Kentucky); Andrea L. Shaffer, BS (University of Kentucky); Sarah Hatch, MSW (University of Kentucky); Sierra Fuhrmann, BS (University of Kentucky); Molly Harper, MSc (University of Kentucky); Kelly Parsons, MSW (University of Kentucky); Jeffrey N. Keller, PhD (Pennington Biomedical Research Center); William P. Gahan, MD (Pennington Biomedical Research Center); Robert Brouillette, MS (Pennington Biomedical Research Center); Heather Foil, MS (Pennington Biomedical Research Center); Owen Carmichael, PhD (Pennington Biomedical Research Center); Paul B. Rosenberg, MD (Johns Hopkins University); Meghan Schultz, RN, MSN (Johns Hopkins University); Samantha Schwartz (Johns Hopkins University); Samantha Horn (Johns Hopkins University); Mersania Jn Pierre (Johns Hopkins University); Robert A. Stern, PhD (Boston University School of Medicine); Jane Mwicigi, MBChB, MPH (Boston University School of Medicine); Alex Puleio, MS (Boston University School of Medicine); Jesse Mez, MD, MS (Boston University School of Medicine); Wendy Qiu, MD, PhD (Boston University School of Medicine); Eric Steinberg, MSN, RN, CS, CANP (Boston University School of Medicine); Tia Hall, BS (Brigham and Women’s Hospital); Emily Sprague, BS (Brigham and Women’s Hospital); Mariana Palou, BS (Brigham and Women’s Hospital); Martha Vander Vliet, RN (Brigham and Women’s Hospital); Jaimie Ziolkowski, MA, CCRP (University of Michigan); Judith L. Heidebrink, MD, MS (University of Michigan); Bekkie Wang (University of Michigan); David S. Knopman, MD (Mayo Clinic – Rochester); Bronwyn Briseno, RN (Mayo Clinic – Rochester); Jonathan Graff Radford, MD (Mayo Clinic – Rochester); Sara Mason, RN (Mayo Clinic – Rochester); Karen Kuntz (Mayo Clinic – Rochester); Kari Baxter (Mayo Clinic – Rochester); Randall Bateman, MD (Washington University School of Medicine); Joy Snider, MD, PhD (Washington University School of Medicine); Gregory Day, MD (Washington University School of Medicine); Nupur Ghoshal, MD, PhD (Washington University School of Medicine); Erik Musiek, MD, PhD (Washington University School of Medicine); Tammie Benzinger, MD, PhD (Washington University School of Medicine); John Morris, MD (Washington University School of Medicine); Marta Santos, BSN, RN (Washington University School of Medicine); Daniel L. Murman, MD, MS (University of Nebraska Medical Center); Haley Kampschneider, BS (University of Nebraska Medical Center); Deb Heimes, BS (University of Nebraska Medical Center); Nick Miller, BS (University of Nebraska Medical Center); David Wint, MD (Cleveland Clinic Lou Ruvo Center for Brain Health); Charles Bernick, MD (Cleveland Clinic Lou Ruvo Center for Brain Health); Michelle Torreliza (Cleveland Clinic Lou Ruvo Center for Brain Health); Simrit Saraon, NP (Cleveland Clinic Lou Ruvo Center for Brain Health); Barnett Shpritz (Cleveland Clinic Lou Ruvo Center for Brain Health); Karen L. Bell, MD (Columbia University Medical Center); Ruth Tejeda, MD, MS (Columbia University Medical Center, Past Coordinator); Chismary De La Cruz, BA (Columbia University Medical Center); Lawrence Honig, MD, PhD (Columbia University Medical Center); Betina Idna, PhD, RN (Columbia University Medical Center); Horacio A. Capote, MD (Dent Neurologic Institute); Michelle Rainka, PharmD (Dent Neurologic Institute); Traci Aladeen, PharmD (Dent Neurologic Institute); Tatiana Jimenez-Knight, MA (Dent Neurologic Institute); Healther MacNamara, BS (Dent Neurologic Institute); Mary Sano, PhD (Icahn School of Medicine at Mount Sinai); Judith Neugroschl, MD (Icahn School of Medicine at Mount Sinai); Joanne Lim (Icahn School of Medicine at Mount Sinai); Allison Ardolino (Icahn School of Medicine at Mount Sinai);Gina Garcia Camilo (Icahn School of Medicine at Mount Sinai); Amy Aloyisi, MD (Icahn School of Medicine at Mount Sinai); Melanie Shulman, MD (NYU Langone Medical Center); Anaztasia Ulysse, BA, CRC (NYU Langone Medical Center); Jamika Singleton-Garvin, CCRC (NYU Langone Medical Center); Mohammed Sheikh, BS, CCRC (NYU Langone Medical Center); Mrunaliniash Gaikwad, BS, CRC (NYU Langone Medical Center); Anton P. Porsteinsson, MD (University of Rochester); Audrey Rice, RN, ANP (University of Rochester); Susan Salem-Spencer, RN, MSN (University of Rochester); Bridget Holvey, MPH, BS (University of Rochester); Asa Widman, BA (University of Rochester); Michael Lin, MD (Weill Cornell Medical Center); Norman Relkin, MD, PhD (Weill Cornell Medical Center); Suzanne Craft, PhD (Wake Forest University School of Medicine); Abigail Heston O’Connell, MS, APRN, NP-C (Wake Forest University School of Medicine); Alexis Webb, MS (Wake Forest University School of Medicine); Bevan Hoover (Wake Forest University School of Medicine); Patricia Wittmer (Wake Forest University School of Medicine); Alan J. Lerner, MD (University Hospitals Cleveland/Case Western Reserve University); Maria Toth, RN (University Hospitals Cleveland/Case Western Reserve University); Parianne Fatica, CCRC (University Hospitals Cleveland/Case Western Reserve University); Susie Sami, MA, (University Hospitals Cleveland/Case Western Reserve University); Paula Ogrocki, PhD (University Hospitals Cleveland/Case Western Reserve University, LEARN Study PI); Marianne Sanders, RN (University Hospitals Cleveland/Case Western Reserve University); Michael Karathanos, MD (Central States Research, LLC); Christy Lisenbee, BS (Central States Research, LLC); Sarah Land, DO (Central States Research, LLC); Carmen Toegel, LPN (Central States Research, LLC); Aimee L. Pierce, MD (Oregon Health and Science University); Lisa C. Silbert, MD (Oregon Health and Science University); Jeffrey A. Kaye, MD (Oregon Health and Science University); Alexandria A. Ruhf, MS (Oregon Health and Science University); Amy B. Thomas, RN (Oregon Health and Science University); Steven Aurich (Oregon Health and Science University); G. Peter Gliebus, MD (Drexel University); Katherine Rife, BS (Drexel University); Melinda Webster, BS (Drexel University); Christine Barr, RN (Drexel University); Monica Mazurek, RN (Drexel University); Sanjeev N. Vaishnavi, MD, PhD (University of Pennsylvania) Martha Combs, BS, MS (University of Pennsylvania); Jade Uffelman, BS (University of Pennsylvania); Loren Terrill (University of Pennsylvania); Oscar Lopez, MD (University of Pittsburgh, Alzheimer’s Disease Research Center); Thomas Baumgartner, LSW, MPH (University of Pittsburgh, Alzheimer’s Disease Research Center); Sarah Goldberg, LPC (University of Pittsburgh, Alzheimer’s Disease Research Center); Donna Simpson, CRNP, MSN, MPH (University of Pittsburgh, Alzheimer’s Disease Research Center); Cary Zik, MPH (University of Pittsburgh, Alzheimer's Disease Research Center); Stephen P. Salloway, MD, MS (Butler Hospital Memory and Aging Program); Diane Monast, RN, MSN, CNS (Butler Hospital Memory and Aging Program); Vanessa Rua, RN, BSN (Butler Hospital Memory and Aging Program); Jessica Alber, PhD (Butler Hospital Memory and Aging Program); Athene K.W. Lee, PhD (Butler Hospital Memory and Aging Program); Sophia Tarro (Butler Hospital Memory and Aging Program); Brain R. Ott, MD (Rhode Island Hospital, Retired); Chuang-Kuo Wu, MD, PhD (Rhode Island Hospital); Lori A. Daiello, PharmD, ScM (Rhode Island Hospital); Jonathan D. Drake, MD (Rhode Island Hospital); Alisa Omert, RN (Rhode Island Hospital); Hannah Alaimo (Rhode Island Hospital); Jacobo Mintzer, MD (Ralph H. Johnson VA Health Care System); Olga Brawman-Mintzer, MD (Ralph H. Johnson VA Health Care System); Allison Acree, MS, CHES (Ralph H. Johnson VA Health Care System); Heather Allen, BSN, MS (Ralph H. Johnson VA Health Care System); Arthur Williams, BS, BA (Ralph H. Johnson VA Health Care System); Sydney O’Connor, MA (Baylor College of Medicine); Valory Pavlik, PhD (Baylor College of Medicine); Melissa Yu, MD, FAAN (Baylor College of Medicine); Shayla Yonce, BA (Baylor College of Medicine); Joseph C. Masdeu, MD, PhD (Nantz National Alzheimer Center, Houston Methodist); Belen Pascual, PhD (Nantz National Alzheimer Center, Houston Methodist); Micha Bangibin (Nantz National Alzheimer Center, Houston Methodist); Benjamin Batista (Nantz National Alzheimer Center, Houston Methodist); Brendan Kelley, MD (University of Texas Southwestern Medical Center); Shahera Ranjha, MS (University of Texas Southwestern Medical Center); Jana Windsor, MS (University of Texas Southwestern Medical Center); Mary Quiceno, MD (University of Texas Southwestern Medical Center, Past Investigator); Elaine Peskind, MD (Seattle Institute for Biomedical & Clinical Research); James O’Connell, MSW (Seattle Institute for Biomedical & Clinical Research); Adam McPartlin, ARNP (Seattle Institute for Biomedical & Clinical Research); Murray A. Raskind, MD, PhD (Seattle Institute for Biomedical & Clinical Research); Anita Ranta, BS (Seattle Institute for Biomedical & Clinical Research); Cynthia M. Carlsson, MD, MS (University of Wisconsin); Benjamin Farral, BS (University of Wisconsin); Kim Peterson, BS (University of Wisconsin); Sandra Harding, MS (University of Wisconsin); Aleshia Cole, RN, APNP (University of Wisconsin); Sarah Best, BSc, CCRP, MHM (Candidate) (Parkwood Institute); Rebecca Shostak, RPN (Parkwood Institute); Kayla VanderPloeg, BScN (Parkwood Institute); Elsa Mann, BScN (Parkwood Institute); Julia Truemner, BA, CCRP (Parkwood Institute); Sandra Black, OC, OOnt, MD, FRCP(C), FRSC, FAAN, FAHA, FANA (Sunnybrook Health Sciences Centre); Benjamin Lam, MD, MSc, FRCP(C) (Sunnybrook Health Sciences Centre); Chinthaka Heyn, PhD, MD, FRCP(C) (Sunnybrook Health Sciences Centre); Samantha Paul-Stotz, MN-RN, BSN (Sunnybrook Health Sciences Centre); Maryna Butenko, MSc (Sunnybrook Health Sciences Centre); Sharon Cohen, MD, FRCPC (Toronto Memory Program); C. Ian Cohen, MD, CCFP (Toronto Memory Program); Atif Shaikh, MBBS, RPN (Toronto Memory Program); Ellen Buchman, MD, CCFP (Toronto Memory Program); Barathy Tharmalingam, CRC (Toronto Memory Program); Linda Schlesinger, BA, CCRP (Toronto Memory Program); Robin Hsiung, MD, MHSc, FRCPC, FACP, FAAN (University of British Columbia, Clinic for Alzheimer Disease and Related Disorders); Ellen Kim, MSc (University of British Columbia, Clinic for Alzheimer Disease and Related Disorders); Tahlee Marian Bpsych (University of British Columbia, Clinic for Alzheimer Disease and Related Disorders); Haakon Nygaard, MD, PhD (University of British Columbia, Clinic for Alzheimer Disease and Related Disorders); Benita Mudge, BSc (University of British Columbia, Clinic for Alzheimer Disease and Related Disorders); Michele Assaly, MA (University of British Columbia, Clinic for Alzheimer Disease and Related Disorders); Colin L. Masters, MD (The University of Melbourne); Andrew Huynh, MBBS, BMedSci, Mclin Tres (The University of Melbourne); Paul Yates, MBBS, PhD, FRACP (The University of Melbourne); Georgios Zisis, RN, MSc (The University of Melbourne); Laura Marginson, RN (The University of Melbourne); Takeshi Iwatsubo, MD, PhD (The University of Tokyo, School of Medicine); Atushi Iwata, MD, PhD (The University of Tokyo, School of Medicine); Kazushi Suzuki, MD, PhD (The University of Tokyo, School of Medicine); Yoshiki Niimi MD, PhD (The University of Tokyo, School of Medicine); Ken-ichiro Sato, MD, PhD (The University of Tokyo, School of Medicine).

## DELCODE study group

**Others collaborators of the current project**

| **Name** | **Affiliations** |
| --- | --- |
| **Claudia Bartels** | Department of Psychiatry and Psychotherapy, University Medical Center Goettingen, University of Goettingen, Von-Siebold-Str. 5, 37075 Goettingen |
| **Klaus Fliessbach** | 1. German Center for Neurodegenerative Diseases (DZNE), Bonn, Venusberg-Campus 1, 53127 Bonn, Germany  2. Department for Cognitive Disorders and Old Age Psychiatry, University Hospital Bonn, Bonn, Germany |
| **Silka Dawn Freiesleben** | 1. German Center for Neurodegenerative Diseases (DZNE), Berlin, Germany  2. Charité – Universitätsmedizin Berlin, corporate member of Freie Universität Berlin and Humboldt-Universität zu Berlin-Institute of Psychiatry and Psychotherapy |
| **Ingo Frommann** | 1. German Center for Neurodegenerative Diseases (DZNE), Bonn, Venusberg-Campus 1, 53127 Bonn, Germany  2. Department for Cognitive Disorders and Old Age Psychiatry, University Hospital Bonn, Bonn, Germany |
| **Wenzel Glanz** | German Center for Neurodegenerative Diseases (DZNE), Magdeburg, Germany |
| **Julian Hellmann-Regen** | 1. German Center for Neurodegenerative Diseases (DZNE), Berlin, Germany  2. Charité Universitätsmedizin Berlin, Department of Psychiatry and Neurosciences, Campus Benjamin Franklin  3. German Center for Mental Health (DZPG), partner site Berlin |
| **Daniel Janowitz** | Institute for Stroke and Dementia Research (ISD), University Hospital, LMU Munich, Feodor-Lynen-Strasse 17, 81377 Munich, Germany |
| **Ingo Kilimann** | 1. German Center for Neurodegenerative Diseases (DZNE), Rostock, Germany  2. Department of Psychosomatic Medicine, Rostock University Medical Center, Gehlsheimer Str. 20, 18147 Rostock |
| **Lukas Preis** | Charité – Universitätsmedizin Berlin, corporate member of Freie Universität Berlin and Humboldt-Universität zu Berlin-Institute of Psychiatry and Psychotherapy |
| **Boris Rauchmann** | 1. Department of Psychiatry and Psychotherapy, University Hospital, LMU Munich, Munich, Germany  2. Sheffield Institute for Translational Neuroscience (SITraN), University of Sheffield, Sheffield, UK  3. Department of Neuroradiology, University Hospital LMU, Munich, Germany |
| **Sandra Roeske** | German Center for Neurodegenerative Diseases (DZNE), Bonn, Venusberg-Campus 1, 53127 Bonn, Germany |
| **Ayda**  **Rostamzadeh** | Department of Psychiatry, University of Cologne, Medical Faculty, Kerpener Strasse 62, 50924 Cologne, Germany |
| **Nina Roy-Kluth** | German Center for Neurodegenerative Diseases (DZNE), Bonn, Venusberg-Campus 1, 53127 Bonn, Germany |
| **Sebastian Sodenkamp** | German Center for Neurodegenerative Diseases (DZNE), Tübingen, Germany |
| **Eike Spruth** | 1. German Center for Neurodegenerative Diseases (DZNE), Berlin, Germany  2. Department of Psychiatry and Psychotherapy, Charité, Charitéplatz 1, 10117 Berlin, Germany |

**Other members of the DELCODE study group**

Slawek Altenstein, Holger Amthauer, Abdelmajid Bader, Juan Carlos Baldermann, Miriam Barkhoff, Henning Boecker, Martina Buchmann, Lena Burrow, Arda Can Cetindag, Nicoleta Carmen Cosma, Lisa Coloma Andrews, Marcel Daamen, Sylvia De Jonge, Peter Dechent, Dominik Diesing, Martin Dichgans, Laura Dobisch, Alexander Drzezga, Martin Dyrba, Marie Ehrlich, Tanja Engels, Esrin Ersözlü, Birgit Ertl-Wagner, Jennifer Faber, Peter Falkai, Frederik Fenski, Christiana Franke, Daniella Frimmer, Manuel Fuentes, Natalie Garcia Angarita, Thomas Görlitz, Doreen Grieger- Klose, Marcus Grobe- Einsler, Selim Üstün Gürsel, Niels Hansen, Katja Hardenacke, Deike Hartmann, Lina Hassoun, Dietmar Hauser, Tanja Heger, Guido Hennes, Gabi Herrmann, Petra Hinderer, Sina Hirschel, Ildiko Horvath, Brigitte Huber, Nicole Hujer, Enise Irem Incesoy, Heike Janecek-Meyer, Lorraine Jost, Christian Kainz, Pascal Kalbhen, Okka Kimmich, Xenia Kobeleva, Barbara Kofler, Max Kreuser, Elke Kuder-Buletta, Carolin Kurz, CatharinaLange, Chris Lappe, Esther Lau, Katja Lindner, Andrea Lohse, Hannah Lützerath, Franziska Maier, Anja Martikke, Cornelia McCormick, Dix Meiberth, Eva Meisenzahl-Lechner, Herlind Megges, Coraline Metzger, Lisa Miebach, Carolin Miklitz, Claudia Müller, Matthias Munk, Christian Mychajliw, Sabine Nuhn, Demet Oender, Henrike Pfaff, Ilona Pfahlert, Lena Rausch, Axel Rominger, Nasim Roshan Ghiasi, Christin Ruß, Petr Sabik, Lena Sannemann, Christine Schneider, Ann-Katrin Schild, Jennifer Schmid, Monika Schmidt, Björn Schott, Heike Schulz, Franziska Schulze, Sarah Schwarzenboeck, Anna Seegerer, Surjo Soekadar, Susanne Sorgalla, Oliver Speck, Melina Stark, Julia Stephan, Simone Stockter, Patricia Sulzer, ManuelaThelen, Theresia Trunk, Maike Tscheuschler, Franziska Uhle, Julia Utecht, Ina Vogt, Marc-Andre Weber, Steffen Wolfsgruber, Renat Yakupov, Heike Zech, Philip Zeyen, Gabriel Ziegler, Adelgunde Zollver
